# Design, synthesis and anticancer activity studies of 3-(coumarin-3-yl)-acrolein derivatives: Evidenced by integrating network pharmacology and *vitro* assay

**DOI:** 10.3389/fphar.2023.1141121

**Published:** 2023-03-23

**Authors:** Lexian Chen, Qianqian Lv, Jianghong Cai, Jiajie Liang, Ziyan Liang, Jiahui Lin, Ying Xiao, Ruiyao Chen, Zhiling Zhang, Yue Hong, Hong Ji

**Affiliations:** ^1^ Guangzhou Municipal and Guangdong Provincial Key Laboratory of Molecular Target and Clinical Pharmacology, The NMPA and State Key Laboratory of Respiratory Disease, School of Pharmaceutical Sciences and the Fifth Affiliated Hospital, Guangzhou Medical University, Guangzhou, China; ^2^ State Key Laboratory of Quality Research in Chinese Medicine, School of Pharmacy, Macau University of Science and Technology, Taipa, China

**Keywords:** coumarin, acrolein, synthesis, antitumor activity, network pharmacology, PI3K/AKT pathway

## Abstract

Coumarin derivatives have diverse structures and show various significant biological activities. Aiming to develop more potent coumarin derivatives for cancer treatment, a series of coumarin acrolein hybrids were designed and synthesized by using molecular hybridization approach, and investigated for their antiproliferative activity against A549, KB, Hela and MCF-7 cancer cells as well as HUVEC and LO2 human normal cells. The results indicated that most of the synthesized compounds displayed remarkable inhibitory activity towards cancer cells but low cytotoxicity on normal cells. Among all the compounds, **5d** and **6e** were the most promising compounds against different cancer cell lines, especially for A549 and KB cells. The preliminary action mechanism studies suggested that compound **6e**, the representative compound, was capable of dose-dependently suppressing migration, invasion and inducing significant apoptosis. Furthermore, the combined results of network pharmacology and validation experiments revealed that compound **6e** induced mitochondria dependent apoptosis *via* the PI3K/AKT-mediated Bcl-2 signaling pathway. In summary, our study indicated compound **6e** could inhibit cell proliferation, migration, invasion and promote cell apoptosis through inhibition of PI3K/AKT signaling pathway in human oral epidermoid carcinoma cells. These findings demonstrated the potential of 3-(coumarin-3-yl)-acrolein derivatives as novel anticancer chemotherapeutic candidates, providing ideas for further development of drugs for clinical use.

## 1 Introduction

**FIGURE 1:**
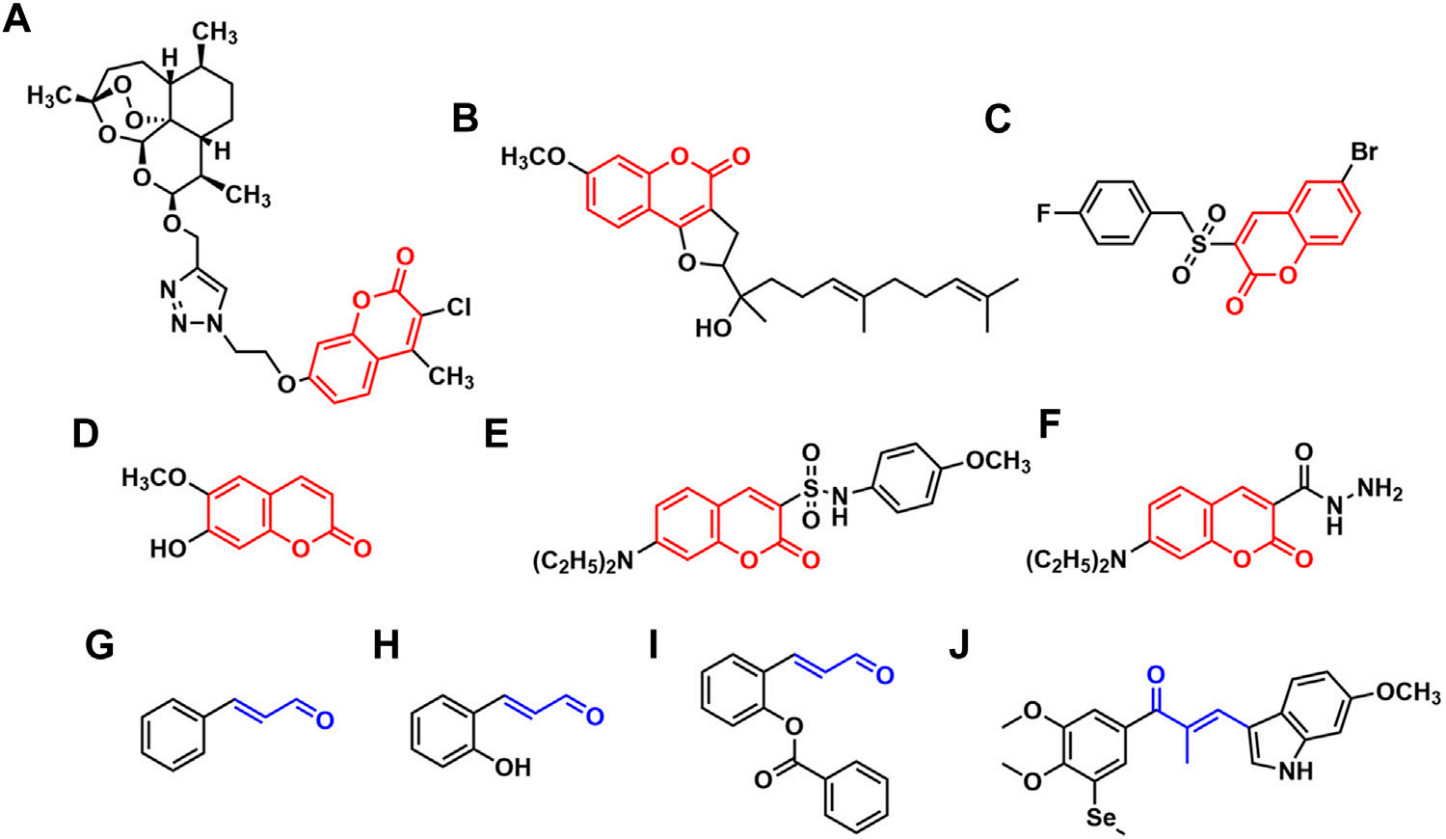
The reported coumarin derivatives **(A–F)** and the derivatives containing acrolein moiety **(G–J)**.

Coumarin derivatives are the heterocyclic compounds that contain the structure of 1,2-benzopyrone, widespread in nature as potent secondary metabolites with a variety of excellent pharmacological activities such as anti-inflammatory (Grover and Jachak, 2015), antioxidation (Singh et al., 2020), antibacterial (Charmforoshan et al., 2022), antithrombosis (Sashidhara et al., 2012), anti-HIV (Xu et al., 2021) and antitumor (Wu et al., 2020). Because of their outstanding antitumor activity and relatively low side effects (Cai et al., 2013; Ahmed et al., 2021; de la Cruz-Concepción et al., 2021; Chu et al., 2022; Song et al., 2022), coumarin derivatives have attracted tremendous research interest. It was reported that the coumarin derivatives connected with dihydroartemisinic (Figure 1A) exhibited potent cytotoxicity against human colon and breast cancer cells through inhibition of carbonic anhydrase IX (Yu et al., 2018). Ferulin C (Figure 1B), a natural sesquiterpene coumarin isolated from *Ferula ferulaeoides*, was found to show cytotoxicity against breast cancer cells. Its mechanism of promoting autophagy by inhibiting PI3K/AKT/mTOR signaling pathway was revealed (Yao et al., 2020). Wang et al. (2019) synthesized a series of benzylsulfone coumarin derivatives and identified compound C (Figure 1C) as a promising PI3K inhibitor, which displayed good antitumor activities against Hela cells by retarding cell migration. Meanwhile, low toxicity and side effects have been constantly found in a lot of coumarin derivatives as medicinal candidates. Scopoletin (Figure 1D) was reported as a potential angiogenesis inhibitor with no effect on normal cell (Cai et al., 2013). There was no obvious toxicity observed in the coumarin-indole derivatives developed by Song *et al.* as tubulin polymerization inhibitors (Song et al., 2022). Hou’s group synthesized a series of carbohydrate-based coumarin derivatives, which showed high selectivity for carbonic anhydrase IX subtype and potent anti-tumor activity, and also exhibited low hERG cardiac toxicity and acute toxicity (Chu et al., 2022). In recent years, our group has been dedicated to the synthesis and development of new coumarin derivatives as anticancer agents. We reported the derivatives attached a sulfonamide moiety at C-3 position of coumarin nucleus (Figure 1E), and revealed that their mechanism of inducing apoptosis was probably associated with increasing reactive oxygen species (ROS) levels and upregulating caspase-3 expression (Zhang et al., 2021a). In addition, the coumarin-3-hydrazide derivatives (Figure 1F) were developed as new lactate transport inhibitors with low toxicity to normal cells, which induced intracellular lactate accumulation and inhibited lactate uptake (Ji et al., 2021). As mentioned above, coumarin scaffold especially the 3-substituted coumarin derivatives played an important role in drug design and research.

Acrolein moiety is an important unit often found in cytotoxic compounds. It is a highly reactive α, β-unsaturated carbonyl serving as a Michael acceptor which has the ability of reacting with glutathione, DNA, some enzymes or receptors as electrophiles, thus disrupts the function of cells (Kehrer and Biswal, 2000; Kern and Kehrer, 2002; Feng et al., 2006; Tang et al., 2011; Wang et al., 2012). It has been reported that the lead compounds containing acrolein moiety possess diverse therapeutically relevant pharmacological functions (Gugliucci, 2008; Badawy and Rabea, 2013; Zhu et al., 2017; Dhara and Tripathi, 2020), which are likely to be potential antitumor agents. Cinnamaldehyde (Figure 1G) and its derivatives characterized by the presence of acrolein moiety have become promising candidates for the development of anticancer drugs and received extensive research attention (Lee et al., 2007; Kwon et al., 2012). Their mechanisms of antitumor actions such as apoptosis induction, cell cycle arrestment and production of oxidative stress have been revealed (Hong et al., 2016). Lee *et al.* reported that 2-hydroxylcinnamaldehyde (Figure 1H) induced apoptosis in colon cancer cells through extracellular signal regulated kinase (ERK)-dependent NF-κB inactivation (Lee et al., 2005). Ka et al. demonstrated that 2-hydroxylcinnamaldehyde induced a rapid decrease in intracellular levels of antioxidant glutathione and protein thiols, contributing to ROS release, and thus resulted in growth inhibition in human promyelocytic leukemia cells (Ka et al., 2003). Jeong *et al.* found that 2-benzoyloxycinnamaldehyde (Figure 1I) induced cell cycle arrest at G2/M phase by increasing the level of cyclin B1 expression and decreasing the level of cyclin E expression on MCF-7 cells (Jeong et al., 2003). Meanwhile, acrolein moiety was also extensively used as a highly active linker in the design of antitumor agents. Yan’s group designed and synthesized a series of chalcone derivatives via the combination of indole and 3,4-dimethoxy-5-(methylselanyl)phenyl group by acrolein linker, among which the newly obtained compound J (Figure 1J) served as a dual-targeting inhibitor of tubulin and thioredoxin reductases, exhibiting superior anti-proliferative activities towards various human cancer cells with IC_50_ values of 8–35 nM (Yan et al., 2022).

In view of the appealing structural scaffold of coumarin derivatives and the favorable antitumor activity of acrolein moiety, we designed and synthesized a series of 3-(coumarin-3-yl)-acrolein derivatives based on molecular hybridization strategy in continuation of our studies on coumarin derivatives. Their antiproliferative activity against four cancer cell lines and two human normal cell lines was evaluated, and the antitumor effects and potential mechanism of the most potent compound were further investigated.

## 2 Results

### 2.1 Chemistry

**SCHEME 1:**
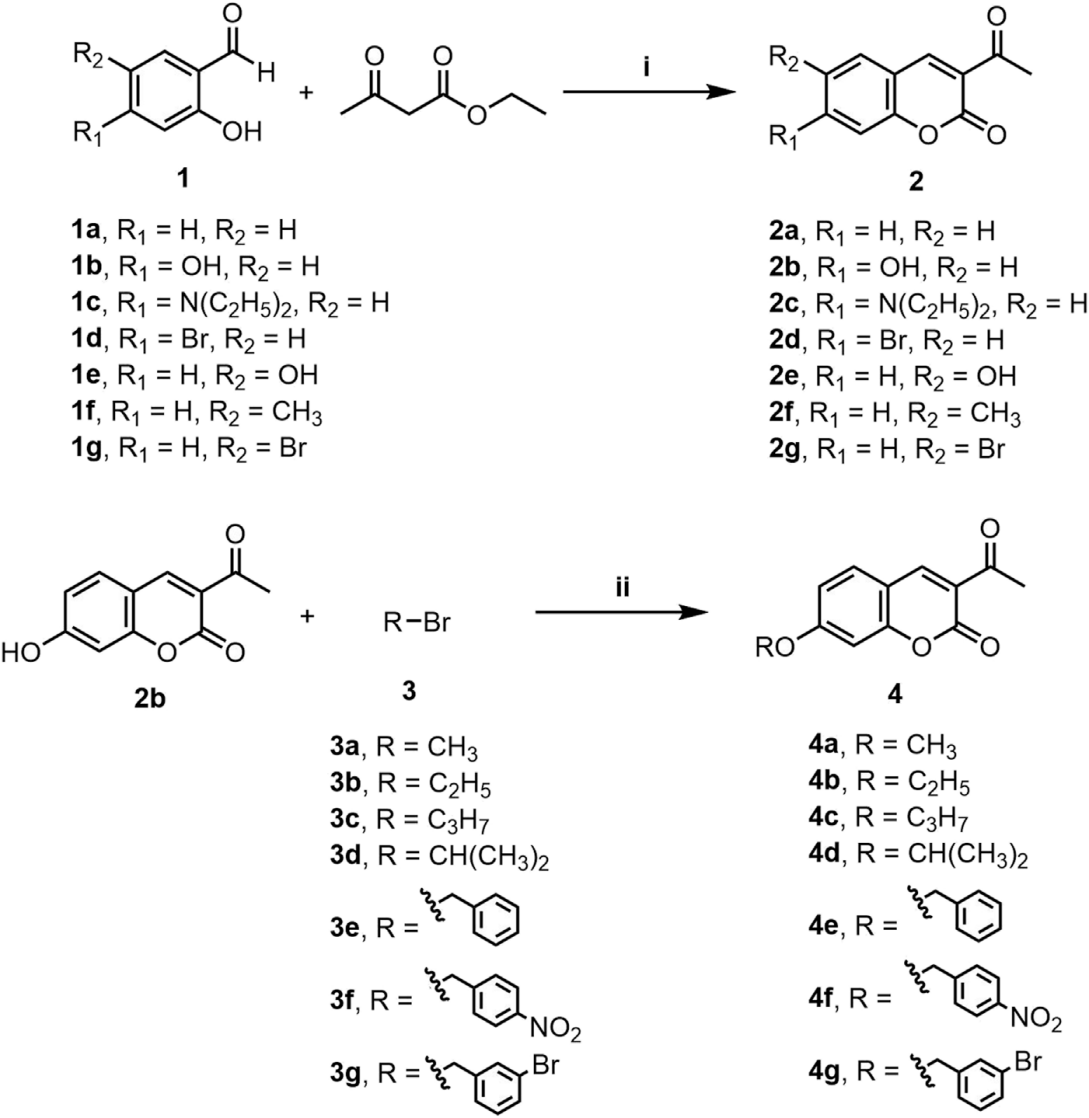
Synthesis of compounds **2a**-**g** and **4a**-**g**. Reagents and conditions: **(i)** Piperidine, C_2_H_5_OH, rt, 0.5–2 h **(ii)** K_2_CO_3_, DMF, N_2_, rt, 16–20 h.

**SCHEME 2:**
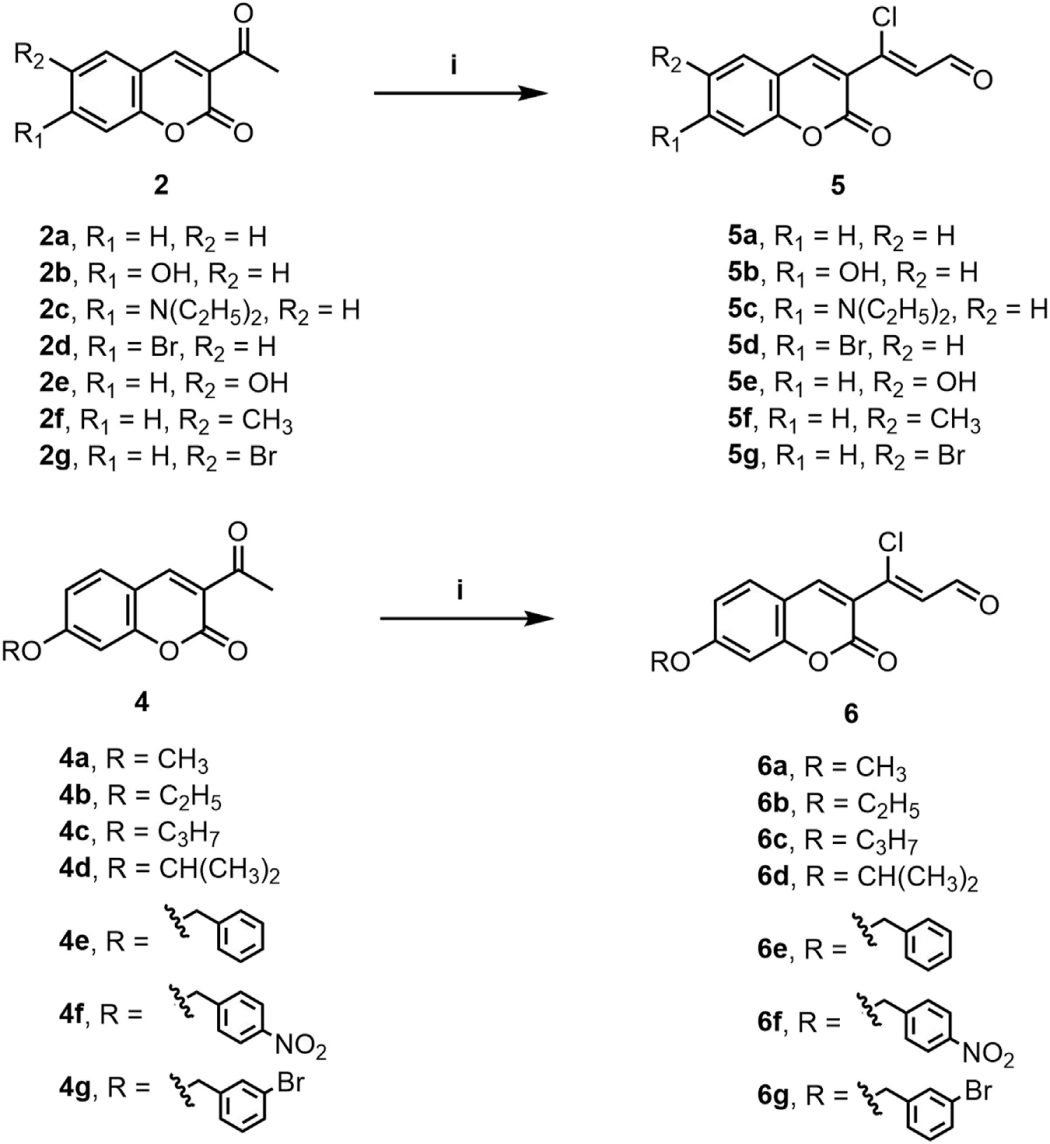
Synthesis of compounds **5a**-**g** and **6a**-**g**. Reagents and conditions: **(i)** POCl_3_, DMF, rt to 60°C–70°C, 5 h.

A series of 3-(coumarin-3-yl)-acrolein derivatives **5a**-**g** and **6a**-**g** were synthesized as described in Schemes 1, 2. The Knoevenagel condensation of substituted salicylic aldehydes **1a**-**g** with ethyl acetoacetate in anhydrous ethanol in the presence of piperidine gave the 3-acetylcoumarins **2a**-**g**. The alkylation of 7-hydroxy-3-acetylcoumarin **2b** with bromides **3a**-**g** in anhydrous *N,N*-dimethylformamide (DMF) at room temperature provided compound **4a**-**g** respectively. Finally, the target 3-(coumarin-3-yl)-acrolein derivatives **5a**-**g** and **6a**-**g** were obtained by the Vilsmeier-Haack-Arnold reaction of the 3-acetylcoumarins (**2a**-**g** or **4a**-**g**) with phosphorus oxychloride (POCl_3_) and DMF. The structures of all the target compounds were confirmed by HRMS, IR and NMR. The spectra can be found in the Supplementary Material.

### 2.2 *In vitro* antiproliferative activity

All the synthesized compounds were screened for *in vitro* antiproliferative activity against four human cancer cell lines A549 (human lung cancer cell line), KB (human oral epidermoid carcinoma cell line), Hela (human cervical carcinoma cell line), MCF-7 (human breast cancer cell line) and two human normal cell lines HUVEC (human umbilical vein endothelial cell) and LO2 (human normal hepatocytes) through the 3-(4,5-dimethyl-2-thiazolyl)-2,5-diphenyl-2-H-tetrazolium bromide (MTT) assay. 5-Fluorouracil (5-FU) was used as positive control and the results are presented in Table 1. It was observed that the majority of the synthesized compounds displayed potent anticancer activity on these cancer cell lines and better inhibition against KB and A549 cell lines. Meanwhile, most of the compounds exhibited low cytotoxicity towards human normal cells, demonstrating their good selectivity for cancer cells.

**TABLE 1:** Antiproliferative activities of compounds **5a**-**g** and **6a**-**g** against different cancer cell lines and human normal cell lines.

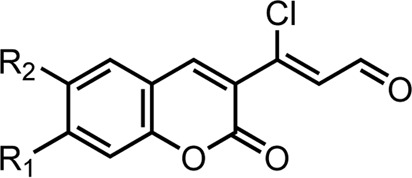								

Compd	R_1_	R_2_	IC_50_ (μM)					
			A549	KB	Hela	MCF-7	HUVEC	LO2
**5a**	H	H	7.97 ± 0.01	1.30 ± 0.02	1.01 ± 0.28	3.55 ± 0.17	22.78 ± 0.35	40.34 ± 0.02
**5b**	OH	H	>100	>100	>100	6.74 ± 0.64	>100	>100
**6a**	OCH_3_	H	2.78 ± 0.01	3.41 ± 0.40	>100	12.38 ± 0.49	10.09 ± 0.07	61.04 ± 0.03
**6b**	OC_2_H_5_	H	11.34 ± 0.07	>100	2.91 ± 0.17	23.29 ± 0.20	>100	>100
**6c**	OC_3_H_7_	H	6.28 ± 0.04	4.54 ± 0.11	>100	1.73 ± 0.01	53.92 ± 0.46	43.52 ± 0.03
**6d**	OCH(CH_3_)_2_	H	5.61 ± 0.01	4.28 ± 0.02	22.7 ± 0.21	51.63 ± 0.13	31.92 ± 0.07	33.27 ± 0.02
**6e**	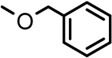	H	7.40 ± 0.20	0.39 ± 0.07	1.82 ± 0.22	14.82 ± 0.28	25.26 ± 0.20	50.13 ± 0.03
**6f**	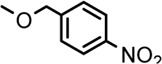	H	>100	>100	>100	>100	>100	>100
**6g**	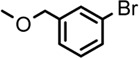	H	>100	>100	>100	5.13 ± 0.07	>100	>100
**5c**	N(C_2_H_5_)_2_	H	7.51 ± 0.02	3.15 ± 0.09	4.85 ± 0.19	18.99 ± 0.18	6.42 ± 0.51	52.65 ± 0.07
**5d**	Br	H	0.70 ± 0.05	1.12 ± 0.03	1.36 ± 0.43	4.23 ± 0.15	10.52 ± 0.02	42.97 ± 0.04
**5e**	H	OH	6.67 ± 0.01	3.23 ± 0.30	>100	8.71 ± 0.31	34.84 ± 0.11	14.11 ± 0.04
**5f**	H	CH_3_	4.57 ± 0.01	2.22 ± 0.04	3.82 ± 0.11	20.06 ± 0.32	27.38 ± 0.24	25.01 ± 0.03
**5g**	H	Br	2.45 ± 0.01	0.87 ± 0.01	13.40 ± 0.22	9.25 ± 0.10	10.90 ± 0.02	66.36 ± 0.09
**5-FU**			7.41 ± 0.09	5.75 ± 2.23	1.37 ± 1.72	19.20 ± 3.29	4.08 ± 0.06	10.68 ± 0.03

3-(coumarin-3-yl)-acrolein derivative **5a** was initially prepared and screened, which showed significant antiproliferative activity comparable to or even stronger than 5-FU against the four cancer cell lines. The influence of the substituents at the C-6 or C-7 position of coumarin nucleus on the activity was then explored. The introduction of 7-hydroxyl group led to compound **5b**, which had no antiproliferative activity on the cancer cells lines except MCF-7. However, when the hydroxyl group was replaced by an alkoxy group such as methoxy, ethoxy, *n*-propoxy or isopropoxy group (**6a**-**d**), the activity was enhanced, and the carbon number of alkoxyl groups showed no significant effect on the antitumor activity. It was excited to find that compound **6e** with benzyloxy group at the C-7 position exhibited potent activity against the four cancer cell lines, especially KB cells, with an IC_50_ of 0.39 ± 0.07 μM, whose antiproliferative activity was 3-fold higher than compound **5a** and 15-fold higher than 5-FU. However, nitro group or bromine substitution on the benzene ring of benzyloxy group resulted in the loss of activity except compound **6g** on MCF-7 cells. In addition, *N,N*-diethylamino group was also introduced to afford compound **5c**, which showed comparable activity to compound **5a** against A549 cells but lower than compound **5a** against the other three cell lines. Interestingly, when the hydrogen at C-7 position of compound **5a** was substituted with bromine, the resulting compound **5d** was 10-fold more potent than **5a** and 5-FU against A549, and had activity equivalent to or greater than that of compound **5a** and 5-FU against KB, Hela and MCF-7 cells. The modification on the C-6 position of the coumarin nucleus also made a great difference to the antiproliferative activity. Hydroxyl, methyl or bromine substituted derivatives **5e**, **5f** and **5g** displayed stronger anticancer activity than 5-FU and **5a** against A549 cells, but a more attenuated activity than **5a** against Hela and MCF-7 cells. It was found that the three derivatives had nearly equivalent activity to compound **5a** against KB cells. For the human normal cell lines HUVEC and LO2, most of the compounds exhibited low antiproliferative activity and all the compounds showed lower cytotoxicity than that of 5-FU. Ultimately, the most potent compound **5d** and **6e** were obtained with IC_50_ values of 0.70 ± 0.05 μM and 0.39 ± 0.07 μM against A549 and KB cancer cell lines, respectively. Compared with the human normal cells, compound **6e** displayed outstanding selectivity to KB cells. The IC_50_ value of compound **6e** against the KB cells was 65-fold lower than that of HUVECs and 129-fold lower than that of LO2 cells, revealing the low cytotoxicity and safety of compound **6e**. Thus, Compound **6e** was chosen for the further study on KB cell lines.

### 2.3 Compound 6e suppressed KB cell migration in wound healing assay and transwell assay

**FIGURE 2:**
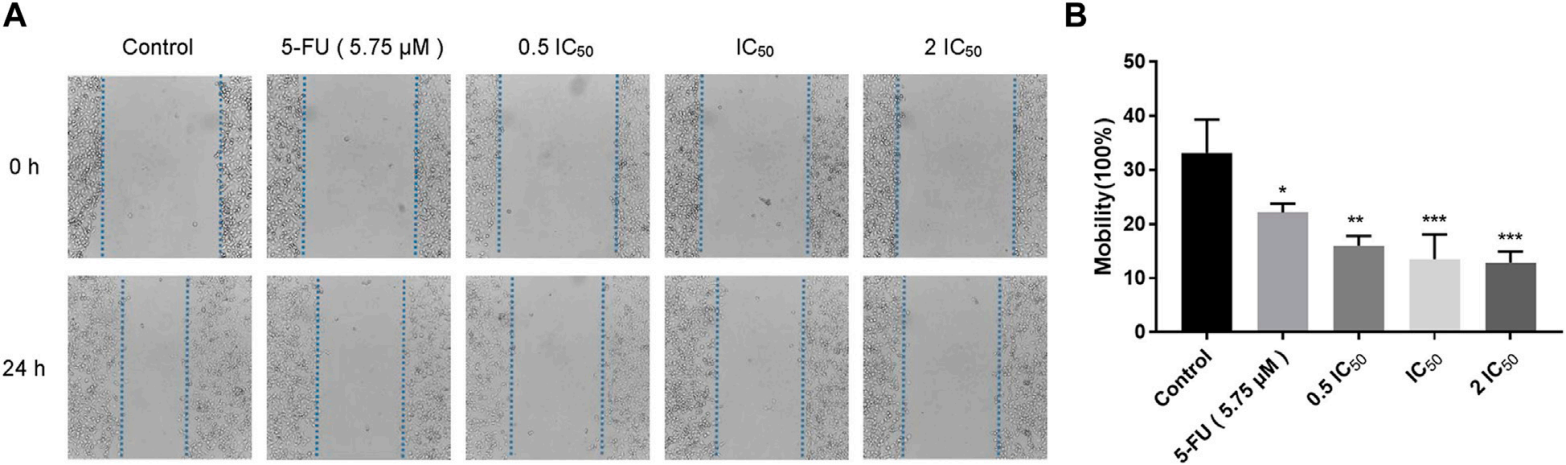
Compound **6e** inhibited wound healing of KB cells. **(A)** The wound was captured at different time points (0 h and 24 h). **(B)** Quantification of the cell scrape results (*n* > 3). *** (*p* < 0.001), ** (*p* < 0.01), * (*p* < 0.05), ns (not significant).

**FIGURE 3:**
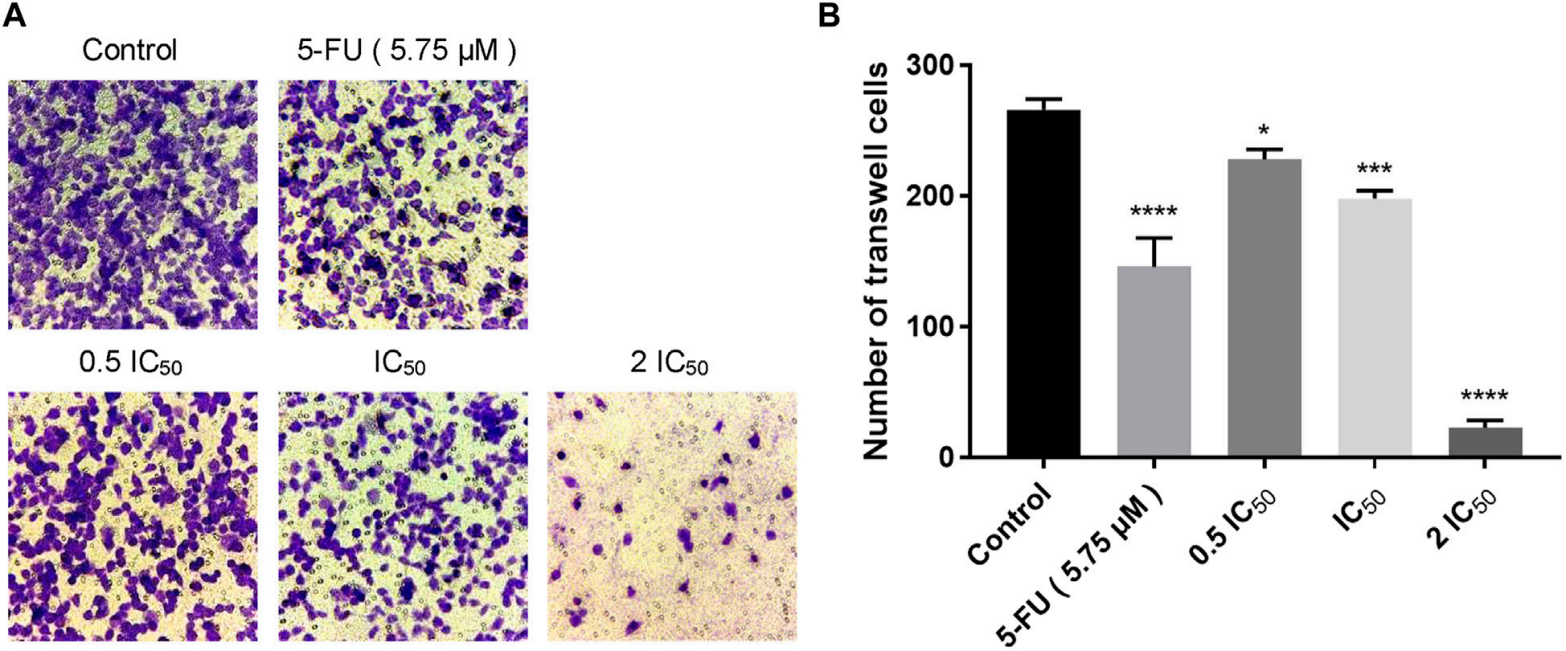
Compound **6e** inhibited space migration of KB cells. **(A)** Microscopic images of spatial migration of KB cells treated with compound **6e**. **(B)** Quantification of the cell space migration results (*n* > 3). **** (*p* < 0.0001), *** (*p* < 0.001), ** (*p* < 0.01), * (*p* < 0.05), ns (not significant).

Wound healing assay and transwell assay were performed to investigate the effect of compound **6e** on the migratory capability of KB cells. Wound healing assay showed that the scratched gaps of KB cells treated with compound **6e** in different concentrations were wider than that of the control group and 5-FU group after 24 h (Figure 2). The result of transwell migration assay also revealed that the treatment of compound **6e** significantly attenuated the migratory ability of KB cells in a concentration-dependent manner (Figure 3). At the concentration of 2 IC_50_, the number of migration cells was significantly reduced by approximately 93% compared with the control group.

### 2.4 Compound 6e inhibited invasion of KB cells

**FIGURE 4:**
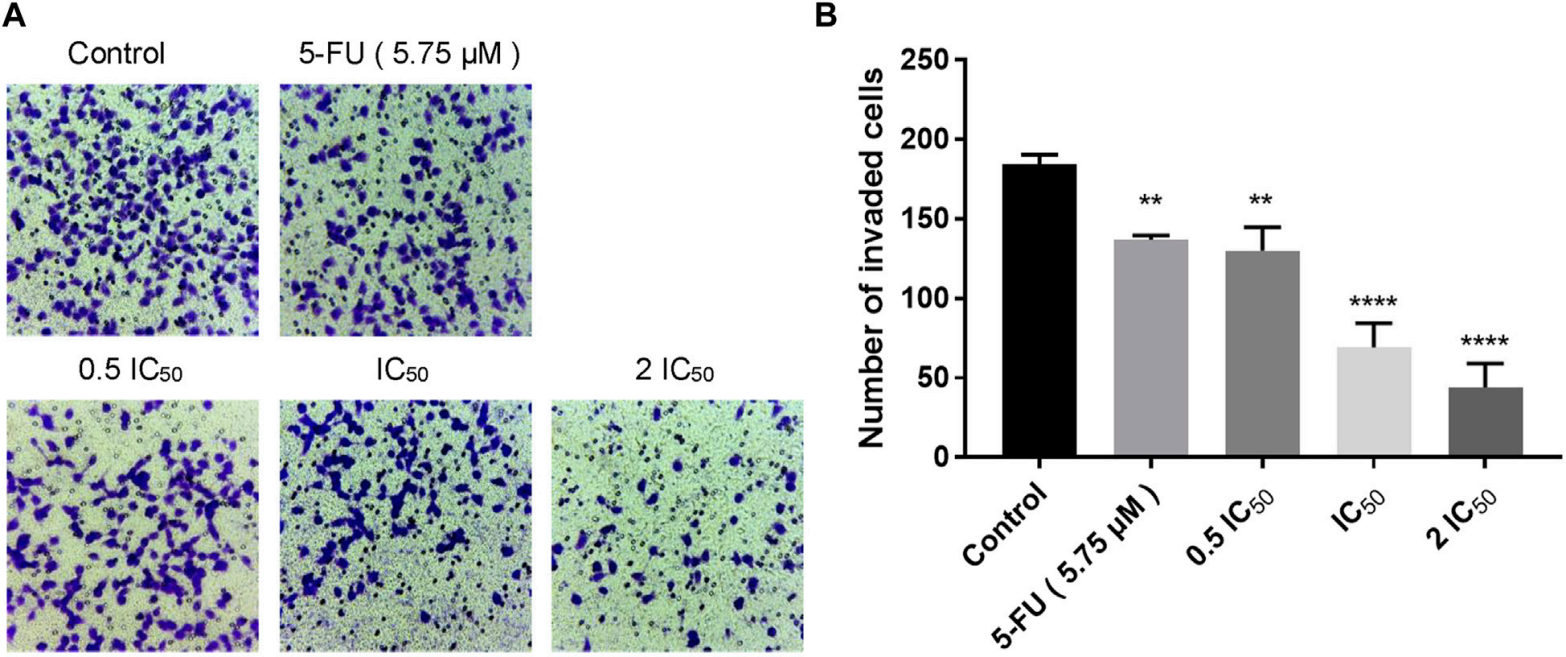
Compound **6e** inhibited invasion of KB cells. **(A)** Microscope images of the KB cells that invaded through the matrigel after treatment of compound **6e**. **(B)** Quantification of the cell invasion results (*n* > 3). **** (*p* < 0.0001), *** (*p* < 0.001), ** (*p* < 0.01), * (*p* < 0.05), ns (not significant).

Transwell invasion assay was carried out to evaluate the effect of compound **6e** on KB cell invasion. The invasion of KB cells was significantly inhibited by compound **6e** in a dose-dependent way. At the highest dose tested (2 IC_50_), compound **6e** reduced KB cell invasion by more than 64% (Figure 4).

### 2.5 Compound 6e induced apoptosis of KB cells

**FIGURE 5:**
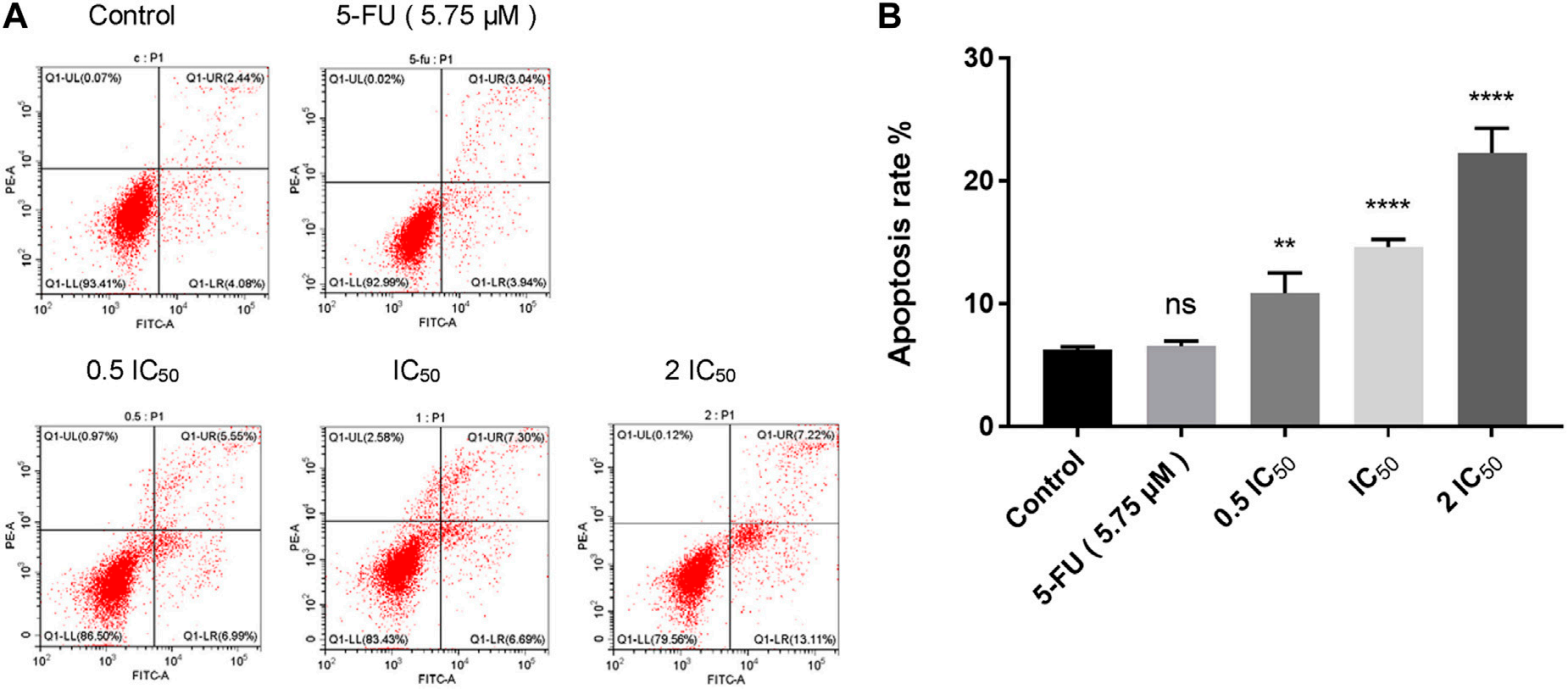
Compound **6e** induced apoptosis of KB cells. **(A)** Dot plot depicting KB cells treated with compound **6e**. **(B)** Quantification of the cell apoptosis results (*n =* 3). **** (*p* < 0.0001), *** (*p* < 0.001), ** (*p* < 0.01), * (*p* < 0.05), ns (not significant).

To investigate whether compound **6e** could induce cell apoptosis, KB cells were treated with compound **6e** in different concentrations for 48 h and the Annexin V-FITC/PI dual staining assay was carried out flow cytometry. As shown in Figure 5, with the increase of the concentrations of compound **6e**, the apoptotic rate in the early stage increased from 4.08% to 6.99%, 6.69% and 13.11%, respectively, and the late apoptotic rate was also significantly higher in compound groups (5.55%, 7.30% and 7.22%) than that of control group (2.44%). The results showed that the total apoptosis rate was increased obviously from 6.52% in control group to 12.54%, 13.99% and 20.33% with the increase of concentration of compound **6e**, which indicated that compound **6e** efficiently promoted the apoptosis of KB cells dose-dependently.

### 2.6 Targets prediction and network construction

Network pharmacology as a novel comprehensive analysis tool based on large databases has been extensively applied in drug research. It focuses on the whole system of potential interactions of drug-target-disease to construct multi-layer networks, which can be applied to decipher the mechanism of drugs action in a holistic view. Several studies suggested that network pharmacology might be helpful to promote the efficiency of drug discovery and development (Pei et al., 2022). In our study, network pharmacology methods were used to explore the potential mechanism of 3-(coumarin-3-yl)-acrolein derivatives including predicting core targets, constructing the target network and performing GO and KEGG pathway enrichment analyses. Finally, the underlying molecular mechanisms were further verified through *in vitro* experiments.

#### 2.6.1 Prediction of potential targets of compound 6e on four types of cancer

**FIGURE 6:**
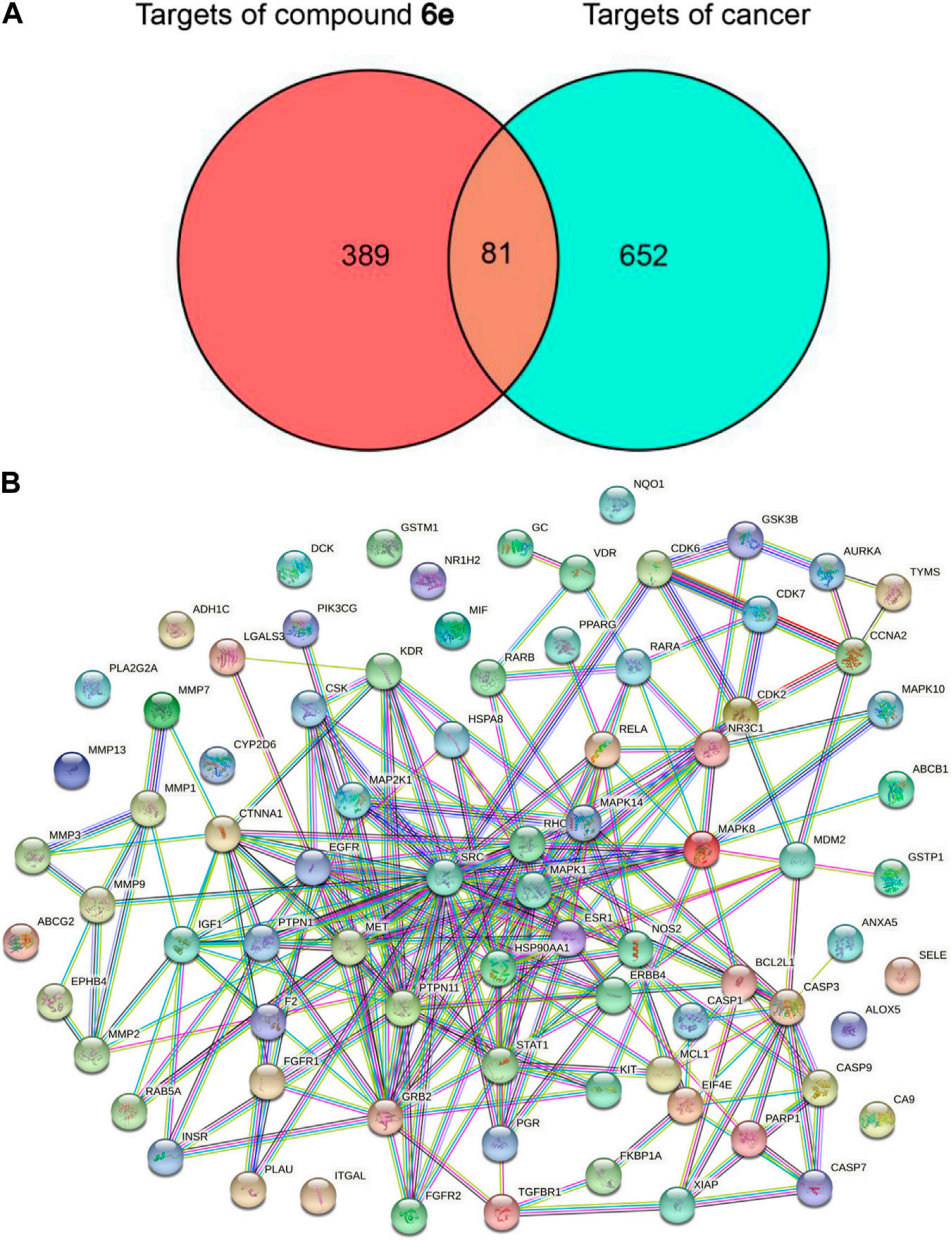
Network construction and PPI network core screening. **(A)** Venn diagram of intersection targets of compound **6e** and four types of cancer. **(B)** PPI network of the overlapping targets of predicted compound **6e** targets and cancer targets obtained from Venn analysis.

The targets associated with the four tested cancer cells were searched from the GeneCards respectively, and 733 potential targets were obtained by overlapping the four sets after eliminating duplicates (Supplementary Figure S1). A total of 470 targets of compound **6e** were collected with the use of databases of PharmMapper, Swiss Target Prediction and TargetNeT. Finally, there were 81 overlapping targets between 733 potential cancer-related targets and 470 potential targets of compound **6e**, which were recognized as the predicted targets of compound **6e** for treating oral epidermoid carcinoma (Figure 6A).

#### 2.6.2 Construction and analysis of protein-protein interaction (PPI) network

The information of the total 81 predicted targets was imported into the String database to obtain the data of PPI and the visualized PPI network. As shown in Figure 6B, the interaction network contained 67 targets (nodes) and 230 relationships (edges). SRC, MAPK1, PTPN11, GRB2 and EGFR as the targets ranked in the top five were regarded as the potential core targets (Supplementary Table S1).

#### 2.6.3 Gene ontology (GO) and kyoto encyclopedia of genes and genomes (KEGG) pathways enrichment analysis

**FIGURE 7:**
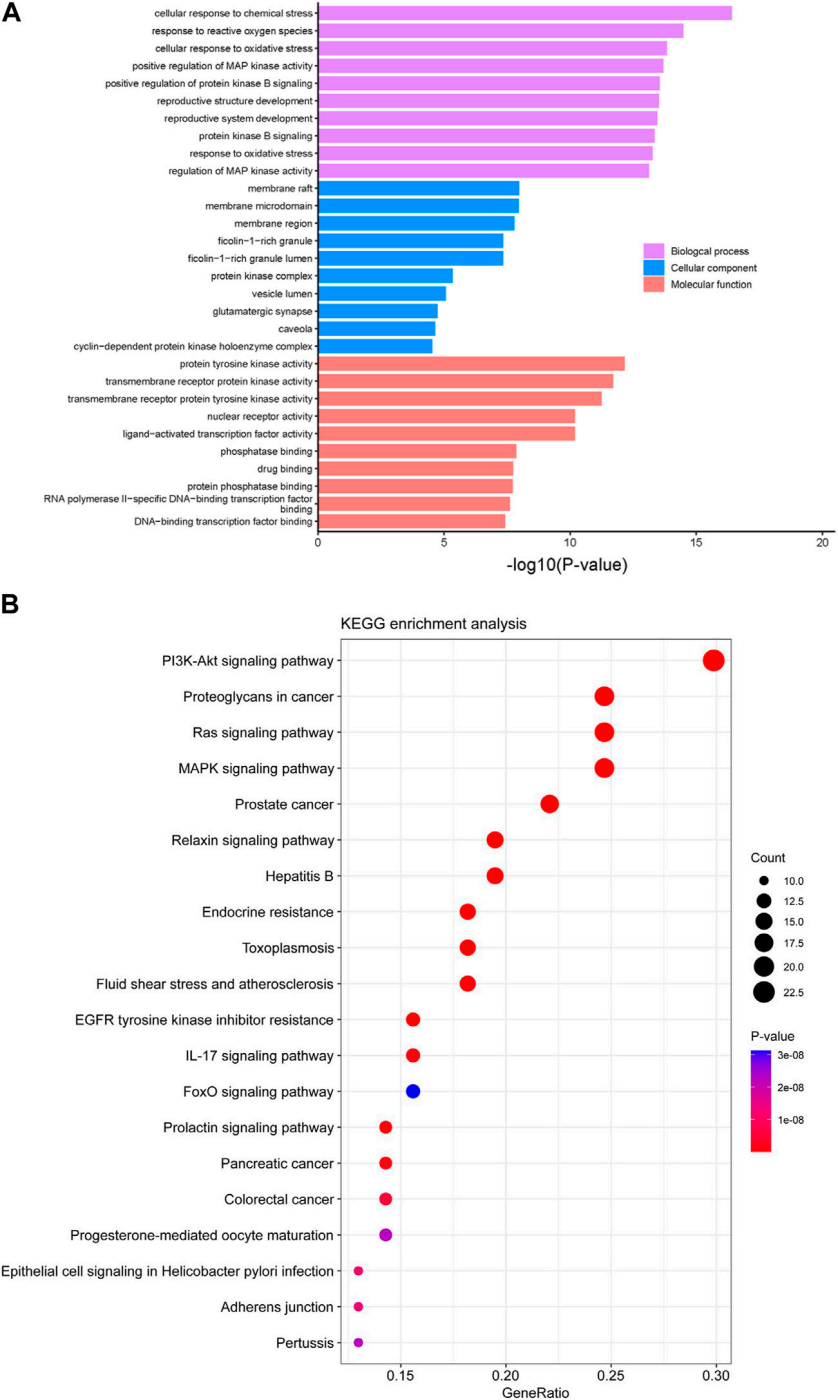
GO and KEGG pathways enrichment analysis. **(A)** GO enrichment analysis of the 81 predicted targets of compound **6e** in the treatment of oral epidermoid carcinoma. **(B)** Top 20 of KEGG pathway enrichment analysis of the 81 predicted targets of compound **6e** in the treatment of oral epidermoid carcinoma.

To realize both the characteristics and functions of these 81 predicted targets, GO enrichment and KEGG pathway enrichment analysis were performed, and the GO terms and KEGG pathways with *p*-value <0.01 were significantly enriched. The top 10 enriched GO terms in biological process, cellular component and molecular function were shown in Figure 7A, among which the response to reactive oxygen species, oxidative stress and protein kinase B (AKT) signaling had significant correlations with oral epidermoid carcinoma. The top 20 related signaling pathways were obtained by KEGG enrichment analysis (Figure 7B), among which PI3K-AKT signaling pathway was the most prominent one. It has been reported that the PI3K-AKT signaling pathway plays a pivotal role in regulating metabolism, proliferation, cell survival, growth and angiogenesis (Li et al., 2014; Wu et al., 2022). In recent years, it has been shown that the PI3K/AKT signaling pathway involved in the above and other processes, are frequently disturbed in many human cancers (Kowshik et al., 2019).

The results of PPI, GO and KEGG pathway enrichments suggested that compound **6e** inhibited cancer cell migration, invasion and induced apoptosis probably through the PI3K/AKT pathway.

### 2.7 Compound 6e reduced mitochondrial membrane potential (MMP) in KB cells

**FIGURE 8:**
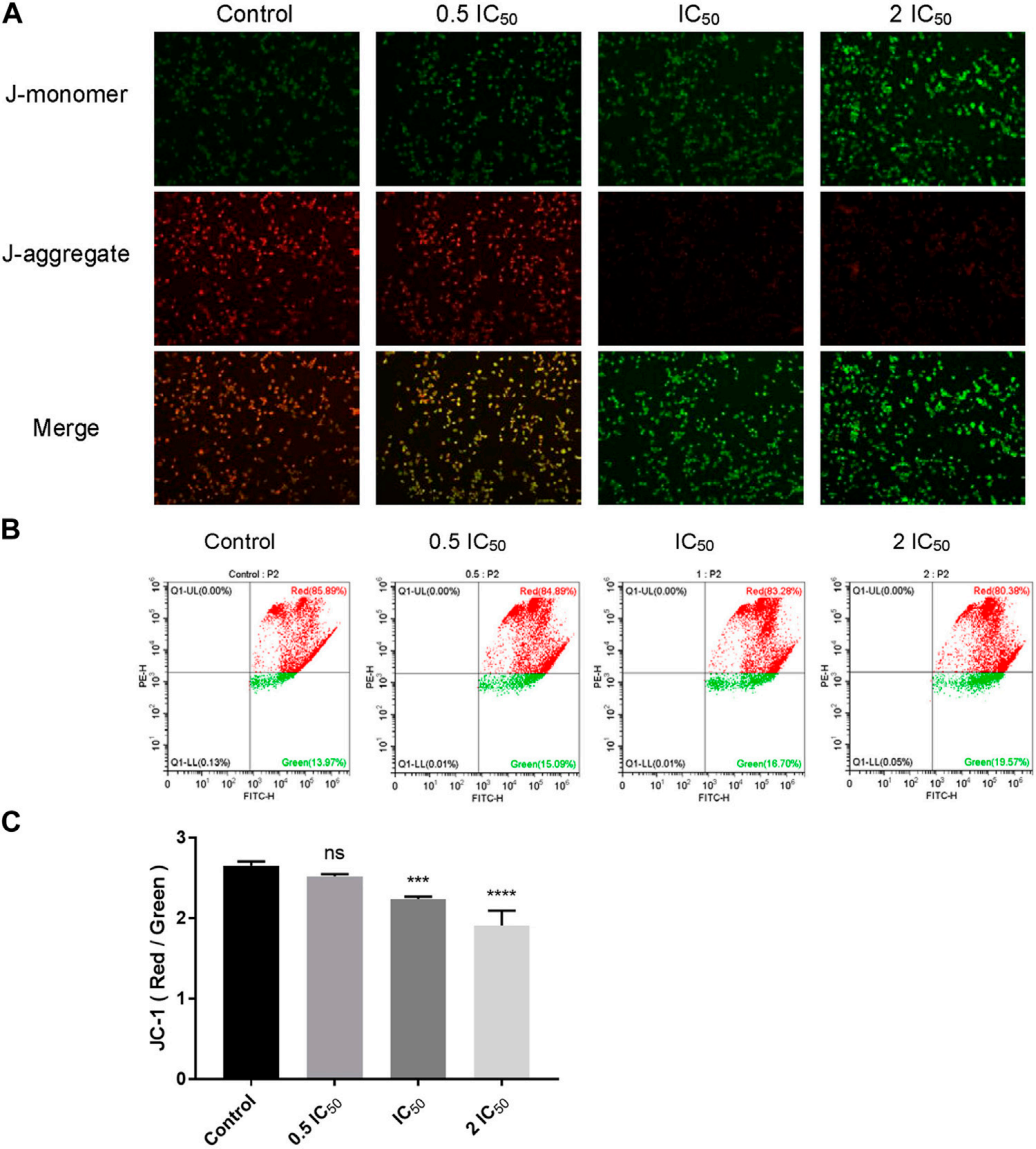
Compound **6e** reduced MMP in KB cells. **(A)** Fluorescence images of KB cells treated with compound **6e**. **(B)** The MMP of KB cells treated with compound **6e** was determined by flow cytometry. **(C)** Quantification of the decreased MMP (*n* = 3). **** (*p* < 0.0001), *** (*p* < 0.001), ** (*p* < 0.01), * (*p* < 0.05), ns (not significant).

According to the predicted results of network pharmacology, compound **6e** might stimulate the oxidative stress response of cancer cells. Oxidative stress reflects an imbalance between excessive production of reactive free radical and deficits in antioxidant biosystem (Zhong et al., 2018). Mitochondria are the main reactors that produce reactive free radicals in cells, and MMP is a key indicator of mitochondrial activity. Excessive oxidative stress can cause a decrease in MMP, which leads to the damage of mitochondrial. It is well-known that mitochondrial damage is a major trigger for the induction of cell apoptosis and necrosis (Kharroubi et al., 2017; Meng et al., 2021). Therefore, JC-1 staining was used to investigate the effect of compound **6e** on the MMP (ΔΨm) of KB cells. In normal cells, JC-1 can form aggregates in healthy mitochondria, emitting red fluorescence, while in apoptotic cells, JC-1 will stay in monomers that emit green fluorescence, implying a low value of ΔΨm. Therefore, mitochondrial depolarization is manifested by a decrease in the ratio of red to green fluorescence intensity. As the concentration of compound **6e** increased, the number of KB cells with red fluorescence decreased in a concentration-dependent manner (Figures 8A, B), and the results also showed a significant decrease in the JC-1 ratio (Figure 8C). These results indicated that compound **6e** reduced the MMP and induced apoptosis in KB cells, which was consistent with the above predicted results.

### 2.8 Western blot analysis

**FIGURE 9:**
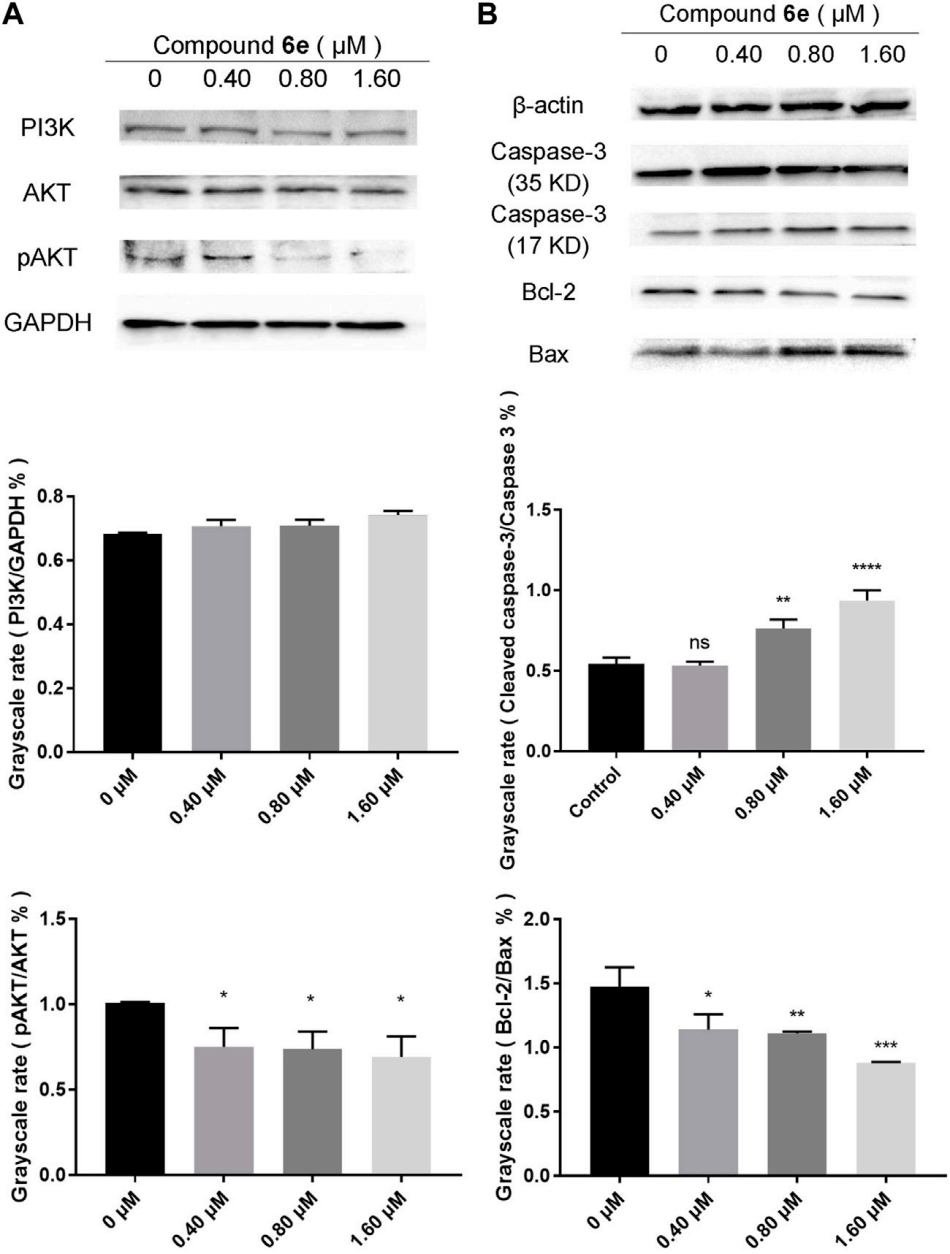
Western blot analysis of related proteins of KB cells treated with compound **6e**. **(A)** The expression levels of the PI3K, AKT and pAKT in KB cells treated with compound **6e**. **(B)** The expression levels of caspase-3 (35 KD, 17 KD), Bcl-2 and Bax in KB cells treated with compound **6e**. Quantification of the protein levels (*n* = 3). **** (*p* < 0.0001), *** (*p* < 0.001), ** (*p* < 0.01), * (*p* < 0.05), ns (not significant).

Based on the results of network pharmacology, the key proteins related to PI3K/AKT pathway were detected by Western blotting to explore the antitumor mechanism of compound **6e**. As shown in Figure 9A, with the increased concentration of compound **6e**, the pAKT/AKT ratios were remarkably decreased, which indicated that compound **6e** could downregulate the level of AKT phosphorylation. However, the relative PI3K level was not significantly different among the control and treatment groups. Such an inconsistency might be due to a compensatory increase in PI3K level after the reduction of AKT phosphorylation level. Additionally, the active-caspase 3 protein, anti-apoptosis protein Bcl-2 and pro-apoptosis protein Bax were further analyzed. Caspase-3 normally exists in the cytoplasm as zymogen. During the early stage of apoptosis, caspase-3 (32 KD) is cleaved to the active 17-kD caspase-3 fragment, which is responsible for morphological and biochemical changes in apoptosis. Bcl-2 and Bax are the cell survival proteins located on the mitochondrial membrane and capable of controlling the MMP (ΔΨm) and the activation of caspase cascade. The results showed that compound **6e** upregulated the expression of the cleaved 17-kD caspase-3 in KB cells at the concentration of 1.60 μM. The ratio of cleaved caspase-3 to caspase-3 was also increased with the treatment of compound **6e** at the concentration of 0.80 μM, indicating the activation level of caspase-3 was increased. Compared with the control group, the expression of Bcl-2 was visibly decreased and that of Bax was upregulated in the groups treated with compound **6e** at the low concentration of 0.40 μM, leading to a decrease in the proportion of Bcl-2 to Bax (Figure 9B). The results indicated that compound **6e** triggered MMP mediated apoptosis of KB cells via PI3K/AKT signaling pathway, which was consistent with the results deduced from network pharmacology analyses.

## 3 Discussion

In this study, we designed and synthesized a series of novel 3-(coumarin-3-yl)-acrolein hybrids **5a**-**g** and **6a**-**g** for further development of anti-tumor drugs. Then we evaluated for their antiproliferative activity against A549, KB, Hela and MCF-7 cancer cell lines as well as HUVEC and LO2 normal cell lines. Compound **5d** and **6e** were identified as the most potent compounds with the IC_50_ values of 0.70 ± 0.05–4.23 ± 0.15 μM and 0.39 ± 0.07–14.82 ± 0.28 μM against the tested cancer cell lines, respectively. Meanwhile, their good selectivity to cancer cells and low cytotoxicity to normal cells were also observed. Due to the highest antiproliferative activity against KB cells and low cytotoxicity to normal human cells, compound **6e** was selected as a representative for subsequent *in vitro* anti-tumor studies, and its underlying anti-tumor mechanism was further discovered.

Since directed migration or invasion of tumor cells into adjacent tissues is identified as one of the hallmarks of cancer (Gerashchenko et al., 2019), migratory and invasive ability of the tumor cells *in vitro* are usually regarded as significant evaluation indicators for the anti-tumor studies (Justus et al., 2014). Wounding healing assay and transwell assay are commonly used to study the migratory response of cancer cells to drug (Wu et al., 2018; Zhang et al., 2021b; Wang et al., 2021). In this study, we used the two assays to evaluate the anti-migratory effect of compound **6e** on KB cells. The former detected the planar movement ability of monolayer cells after external stimulation, while the latter detected spatial migration ability of cells. Transwell invasion assay was also carried out to evaluate the anti-invasion effect of compound **6e**. The results revealed that compound **6e** significantly inhibited the migration and invasion of KB cells in a dose dependent manner (Figures 2, 3, 4).

Apoptosis, a form of programmed cell death, is executed by several processes including DNA fragmentation, reduction of cellular size, disable function of the mitochondria, plasma membrane blebbing, and the production of apoptotic bodies (Tomek et al., 2012). Apoptosis is considered the primary type of cell death, which is induced through relevant signaling pathways (Singh and Lim, 2022). The analysis of Annexin V-FITC/PI dual staining assay proved that compound **6e** induced apoptosis of KB cells in a dose dependent manner. Compared with the untreated group, the percentages of early apoptosis, late apoptosis and total apoptosis increased by 13.11%, 7.22% and 20.33% when KB cells were treated with 2 IC_50_ of compound **6e** for 48 h (Figure 5). Whereas, its underlying mechanism was needed further explored.

Recently, network pharmacology has emerged as a powerful tool to characterize the action mechanisms of undeveloped or complicated derivatives in detail. It has been widely used in mechanism studies of various derivatives for treatment of cancer (Guo et al., 2019; Huang et al., 2021; Zhang et al., 2022). In the present study, network pharmacology-based prediction showed that PI3K-AKT signaling pathway was the top signaling pathway involved in the underlying mechanisms of compound **6e**. The result served as a reference for our subsequent further study (Figures 6, 7). Therefore, we focused on the PI3K-AKT signaling pathway to explore the mechanism underlying the effects of compound **6e** on KB cells. PI3K/AKT pathway plays an important role in a variety of biological processes, such as cell growth, proliferation, differentiation and apoptosis (Noorolyai et al., 2019; Xie et al., 2019; Dong et al., 2020; Li et al., 2021). Western blotting demonstrated that compound **6e** significantly reduced the pAKT/AKT ratio (Figure 9A), down-regulating the level of AKT phosphorylation. These results suggested that compound **6e** might inhibit cell proliferation, migration, invasion and promote apoptosis of KB cells by inhibiting the PI3K-AKT pathway.

Meanwhile, the predicted result from network pharmacology was verified which compound **6e** might stimulate the oxidative stress response of KB cells. Mitochondria are the main reactors that produce reactive free radicals. The mitochondria-dependent apoptotic pathway is regulated by pro- and anti-apoptotic members of the Bcl-2 protein family as well as caspase family (Guerra et al., 2011; Wang et al., 2013). According to Figure 8, compound **6e** decreased the MMP of KB cells. In addition, the expression levels of mitochondria-related apoptosis proteins (Caspase-3, Bcl-2 and Bax) were also changed significantly by the intervention of compound **6e**. Compound **6e** decreased Bcl-2/Bax ratio and increased the level of active-caspase 3, indicating that compound **6e** induced caspase-3-dependent apoptosis in KB cells, as evidenced by Western blot (Figure 9B).

**FIGURE 10:**
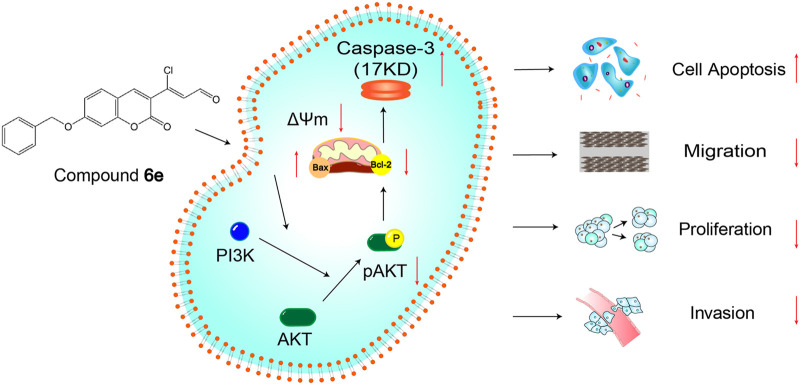
Possible mechanism of the antitumor effects of compound **6e**.

In summary, compound **6e** suppressed cell proliferation, migration, invasion and induced cell apoptosis through inhibition of PI3K/AKT signaling pathway in KB cells (Figure 10). Its mechanism on mitochondria-mediated apoptosis via PI3K-AKT signaling pathway was revealed. These findings demonstrated that compound **6e** was likely to be a promising antitumor agent, providing a possible direction for the clinical treatment of oral epidermoid carcinoma.

## 4 Materials and methods

### 4.1 Chemistry

All of the solvents and reagents are commercially available and were purchased from Accela ChemBio Co., Ltd. (Shanghai, China) without further purification unless otherwise specified. All the reactions were monitored by thin-layer chromatography (TLC) on precoated silica gel GF254 plates (Qingdao Haiyang, Qingdao, Shandong Province, China) and visualized with UV light (254 nm and 365 nm). All melting points were taken on an X-6 microscope melting point instrument (Bjfuka, Beijing, China) which was uncorrected. Infrared (IR) spectra were obtained by a FTIR (Fourier Transform Infrared) spectrometer (Nicolet 6700, United States) and the data were reported in wave numbers (cm^-1^). ^1^H and ^13^C NMR spectra were recorded on 400 MHz JNM-ECZ400S/L1 spectrometers (Varian INOVA, America) with tetramethylsilane (TMS) as an internal reference. The multiplicities were designated by the following abbreviations: s = singlet, d = doublet, t = triplet, q = quartet, m = multiplet, brs = broad singlet. Coupling constant (*J*) is expressed in Hertz (Hz). The detailed procedures and characterizations have been exhibited below. Other additional figures and tables were shown in Supplementary Material.

#### 4.1.1 Synthesis of compounds 2a-g

To a stirred solution of different substituted salicylaldehyde (**1a**-**g**) (7.0 mmol) and ethyl acetoacetate (0.89 mL, 7.0 mmol) in ethanol (20 mL), the catalytic amount of piperidine (0.1 mL) was added dropwise. The reaction mixture was stirred at room temperature for 0.5–2 h and the progress of the reaction was monitored by TLC. After completion of the reaction, the reaction mixture was diluted with deionized water (20 mL) and extracted with ethyl acetate (20 mL × 3). Finally, the combined organic layer was dried over anhydrous Na_2_SO_4_, filtered and concentrated under reduced pressure to give the compounds **2a**-**g** with yields of 48.5%–94.0%. All the synthetic characterization data of compounds **2a**-**g** are outlined in Supplementary Material.

#### 4.1.2 Synthesis of compounds 4a-g

To a stirred solution of compound **2b** (204.2 mg, 1.0 mmol) and K_2_CO_3_ (165.8 mg, 1.2 mmol) in DMF (6 mL), alkyl or benzyl bromines (**3a**-**g**) (1.4 mmol) were added dropwise under an atmosphere of nitrogen at room temperature. The reaction mixture was stirred for 16–20 h and the progress of the reaction was monitored by TLC. After completion of the reaction, the reaction mixture was diluted with deionized water (10 mL) and the obtained precipitate was filtered off. Upon filtering, the isolated solid was washed with deionized water and dried to afford **4a**-**g** as yellow solids (yield: 52.5%–98.5%). All the synthetic characterization data of compounds **4a**-**g** are outlined in Supplementary Material.

#### 4.1.3 General procedures for the preparation of 5a-g

To a stirred solution of phosphorus oxychloride (0.1 mL, 1.1 mmol) in DMF (5 mL), the solution of compounds **2a**-**g** (1.0 mmol) in DMF (2 mL) was added dropwise, and the reaction mixture was stirred at 60°C for 5 h. After completion of the reaction (monitered through TLC), the detailed treatments of purification for compounds **5a-g** followed.

##### 4.1.3.1 3-Chloro-3-(2-oxo-2*H*-chromen-3-yl)acrylaldehyde (5a)

After completion of reaction, the reaction mixture was diluted with deionized water (4 mL) and the precipitated yellow solid was filtered off and recrystallized from ethanol as pure yellow powder. Yield: 30.0%. M. p. 167°C–169 °C. IR (KBr) ν_max_ (cm^-1^) 3058, 1714, 1655, 1594. ^1^H-NMR (400 MHz, DMSO-*d*_*6*_) δ 10.19 (d, *J* = 6.8 Hz, 1H), 8.85 (s, 1H), 7.99 (dd, *J* = 7.8, 1.7 Hz, 1H), 7.74–7.80 (m, 1H), 7.45–7.51 (m, 2H), 7.42 (d, *J* = 6.8 Hz, 1H). ^13^C NMR (100 MHz, CDCl_3_) δ 192.10, 157.40, 153.85, 144.98, 143.32, 134.40, 129.59, 129.33, 125.36, 121.93, 118.45, 116.72. HRMS-ESI: *m/z* Calcd for C_12_H_8_ClO_3_ [M + H]^+^: 235.0162; Found: 235.0163.

##### 4.1.3.2 3-Chloro-3-(7-hydroxy-2-oxo-2H-chromen-3-yl)acrylaldehyde (5b)

After completion of reaction, the reaction mixture was diluted with saturated brine (10 mL), and then extracted with ethyl acetate (20 mL × 3). The aqueous phase was left overnight, the precipitated yellow solid was filtered off and washed with deionized water. Then the solid was dried under vacuum to give pure yellow powder. Yield: 34.3%. M. p. 204°C–205 °C. IR (KBr) ν_max_ (cm^-1^) 3296, 3064, 1717, 1656, 1597. ^1^H-NMR (400 MHz, DMSO-*d*_*6*_) δ 11.57 (s, 1H), 10.16 (ddd, *J* = 6.9, 2.0, 1.0 Hz, 1H), 8.78 (s, 1H), 7.83 (d, *J* = 8.7 Hz, 1H), 7.42 (d, *J* = 6.9 Hz, 1H), 6.97 (d, *J* = 8.6 Hz, 1H), 6.88 (s, 1H). ^13^C NMR (100 MHz, DMSO-*d*_*6*_) δ 192.13, 164.20, 157.50, 155.73, 146.32, 144.61, 132.22, 126.61, 115.73, 114.48, 111.24, 101.68. HRMS-ESI: *m/z* Calcd for C_12_H_8_ClO_4_ [M + H]^+^: 251.0111; Found: 251.0102.

##### 4.1.3.3 3-Chloro-3-(7-(diethylamino)-2-oxo-2*H*-chromen-3-yl)acrylaldehyde (5c)

After completion of reaction, the reaction mixture was diluted with saturated brine (10 mL), and then extracted with ethyl acetate (20 mL × 3). The organic phase was dried over anhydrous Na_2_SO_4_, filtered and concentrated under reduced pressure to give orange red solid. The crude product was purified by silica gel column chromatography (Petroleum ether: ethyl acetate = 1:1) and recrystallized from ethyl acetate to obtain the pure yellow powder. Yield: 78.5%. M. p. 206°C–208 °C. IR (KBr) ν_max_ (cm^-1^) 3066, 1725, 1658, 1595, 1082. ^1^H-NMR (400 MHz, CDCl_3_) δ 10.27 (d, *J* = 6.9 Hz, 1H), 8.37 (s, 1H), 7.67 (d, *J* = 6.9 Hz, 1H), 7.39 (d, *J* = 8.9 Hz, 1H), 6.64 (dd, *J* = 9.0, 2.5 Hz, 1H), 6.47 (d, *J* = 2.3 Hz, 1H), 3.46 (d, *J* = 7.2 Hz, 4H), 1.24 (d, *J* = 7.1 Hz, 6H). ^13^C NMR (100 MHz, DMSO-*d*_*6*_) δ 192.63, 158.56, 157.00, 152.90, 145.49, 145.03, 131.14, 126.20, 113.00, 110.10, 108.48, 96.57, 45.31 (2C), 12.58 (2C). HRMS-ESI: *m/z* Calcd for C_16_H_17_ClNO_3_ [M + H]^+^: 306.0897; Found: 306.0901.

##### 4.1.3.4 3-(7-Bromo-2-oxo-2H-chromen-3-yl)-3-chloroacrylaldehyde (5d)

After stirring at 60°C for 5 h, the reaction mixture was cooled to the room temperature. Sodium acetate (400.0 mg) and deionized water (0.8 mL) were added and the reaction mixture was stirred for 5 h. Then 5% aqueous solution of Na_2_CO_3_ was added to the reaction mixture to adjust the pH to 7–8. The precipitated yellow solid was filtered off, washed with deionized water and dried under vacuum to give pure yellow powder. Yield: 36.4%. M. p. 182°C–183 °C. IR (KBr) ν_max_ (cm^-1^) 3070, 1725, 1661, 1598. ^1^H-NMR (400 MHz, DMSO-*d*_*6*_) δ 10.18 (ddd, *J* = 6.8, 3.0, 1.6 Hz, 1H), 8.84 (d, *J* = 2.2 Hz, 1H), 7.91–7.94 (m, 1H), 7.85 (q, *J* = 2.0 Hz, 1H), 7.66 (ddt, *J* = 6.4, 3.8, 1.7 Hz, 1H), 7.41 (m, 1H). ^13^C NMR (100 MHz, DMSO-*d*_*6*_) δ 192.05, 156.62, 153.51, 145.04, 143.46, 131.58, 128.35, 128.30, 127.50, 121.50, 119.09, 117.72. HRMS-ESI: *m/z* Calcd for C_12_H_7_BrClO_3_ [M + H]^+^: 312.9267; Found: 312.9258.

##### 4.1.3.5 3-Chloro-3-(6-hydroxy-2-oxo-2H-chromen-3-yl)acrylaldehyde (5e)

After stirring at 60 °C for 5 h, the reaction mixture was cooled to the room temperature. Sodium acetate (400.0 mg) and deionized water (0.8 mL) were added and the reaction mixture was stirred for 5 h. After completion of the reaction, the reaction mixture was diluted with saturated brine (10 mL), and then extracted with ethyl acetate (20 mL × 3). The organic phase was dried over anhydrous Na_2_SO_4_, filtered and concentrated under reduced pressure to give an orange solid. The crude product was purified by silica gel column chromatography (Methylene chloride: methanol = 100:1) to obtain pure orange powder. Yield: 45.2%. M. p. 250°C–252°C. IR (KBr) ν_max_ (cm^-1^) 3197, 3072, 1743, 1716, 1648. ^1^H-NMR (400 MHz, DMSO-*d*_*6*_) δ 10.17 (d, *J* = 6.8 Hz, 1H), 9.96 (s, 1H), 8.75 (s, 1H), 7.41 (d, *J* = 6.8 Hz, 1H), 7.33 (d, *J* = 9.0 Hz, 1H), 7.26 (d, *J* = 2.8 Hz, 1H), 7.17 (dd, *J* = 8.9, 2.7 Hz, 1H). ^13^C NMR (100 MHz, DMSO-*d*_*6*_) δ 192.10, 157.31, 154.21, 146.80, 145.75, 143.97, 128.15, 122.79, 121.19, 118.93, 116.99, 113.71. HRMS-ESI: *m/z* Calcd for C_12_H_8_ClO_4_ [M + H]^+^: 251.0111; Found: 251.0116.

##### 4.1.3.6 3-Chloro-3-(6-methyl-2-oxo-2H-chromen-3-yl)acrylaldehyde (5f)

After completion of reaction, the reaction mixture was diluted with deionized water (4 mL). The precipitated yellow solid was filtered off and washed with deionized water. The solid was recrystallized from methylene chloride to give pure yellow powder. Yield: 63.0%. M. p. 179°C–181°C. IR (KBr) ν_max_ (cm^-1^) 3074, 2870, 1712, 1669, 1617. ^1^H-NMR (400 MHz, CDCl_3_) δ 10.28 (d, *J* = 6.7 Hz, 1H), 8.45 (s, 1H), 7.67 (d, *J* = 6.6 Hz, 1H), 7.43 (m, 2H), 7.23 (d, *J* = 3.3 Hz, 1H), 2.42 (s, 3H). ^13^C NMR (100 MHz, CDCl_3_) δ 192.13, 157.59, 152.04, 145.01, 143.51, 135.59, 135.22, 129.21, 129.17, 121.67, 118.20, 116.40, 20.88. HRMS-ESI: *m/z* Calcd for C_13_H_10_ClO_3_ [M + H]^+^: 249.0318; Found: 249.0325.

##### 4.1.3.7 3-(6-Bromo-2-oxo-2H-chromen-3-yl)-3-chloroacrylaldehyde (5g)

After completion of reaction, the reaction mixture was diluted with deionized water (4 mL). The precipitated green solid was filtered off and washed with deionized water. The crude product was dried and purified by silica gel column chromatography (Methylene chloride: methanol = 100:1) to obtain pure yellow powder. Yield: 40.4%. M. p. 199°C–201°C. IR (KBr) ν_max_ (cm^-1^) 3077, 1722, 1667, 1576. ^1^H-NMR (400 MHz, CDCl_3_) δ 10.27 (d, *J* = 6.6 Hz, 1H), 8.41 (s, 1H), 7.78 (d, *J* = 2.4 Hz, 1H), 7.71 (dd, *J* = 8.8, 2.2 Hz, 1H), 7.65 (d, *J* = 6.5 Hz, 1H), 7.23 (d, *J* = 4.0 Hz, 1H). ^13^C NMR (100 MHz, CDCl_3_) δ 191.88, 156.74, 152.60, 143.41, 142.66, 136.99, 131.63, 129.83, 123.03, 119.88, 118.43, 117.91. HRMS-ESI: *m/z* Calcd for C_12_H_7_BrClO_3_ [M + H]^+^: 312.9267; Found: 312.9260.

#### 4.1.4 General procedures for the preparation of 6a-g

To a stirred solution of phosphorus oxychloride (0.1 mL, 1.1 mmol) in DMF (5 mL), the solution of compounds **4a**-**g** (1.0 mmol) in DMF (2 mL) was added dropwise, and the reaction mixture was stirred at 60°C–70°C for 5 h. After completion of the reaction, the reaction mixture was diluted with saturated brine (10 mL), and then extracted with ethyl acetate (20 mL × 3). The aqueous phase was left overnight, the precipitated yellow solid was filtered off and washed with deionized water. Then the solid was dried to give crude product and the product was purified by silica gel column chromatography (Dichloromethane: Petroleum ether = 5:1) to afford the pure yellow powder **6a**-**g**.

##### 4.1.4.1 3-Chloro-3-(7-methoxy-2-oxo-2H-chromen-3-yl)acrylaldehyde (6a)

Yellow solid. Yield: 34.0%. M. p. 202°C–203°C. IR (KBr) ν_max_ (cm^-1^) 3081, 2923, 1713, 1663, 1617. ^1^H-NMR (400 MHz, DMSO-*d*_*6*_) δ 10.17 (d, *J* = 6.8 Hz, 1H), 8.83 (s, 1H), 7.92 (d, *J* = 8.7 Hz, 1H), 7.43 (d, *J* = 6.8 Hz, 1H), 7.11 (m, 1H), 7.06 (dd, *J* = 8.7, 1.8 Hz, 1H), 3.91 (s, 3H). ^13^C NMR (100 MHz, CDCl_3_) δ 192.31, 165.22, 157.72, 156.13, 145.05, 144.01, 130.81, 128.25, 117.96, 114.20, 112.29, 100.30, 56.23. HRMS-ESI: *m/z* Calcd for C_13_H_10_ClO_4_ [M + H]^+^: 265.0268; Found: 265.0263.

##### 4.1.4.2 3-Chloro-3-(7-ethoxy-2-oxo-2H-chromen-3-yl)acrylaldehyde (6b)

Yellow solid. Yield: 27.5%. M. p. 195°C–196°C. IR (KBr) ν_max_ (cm^-1^) 3046, 2938, 2873, 1721, 1665, 1615. ^1^H-NMR (400 MHz, DMSO-*d*_*6*_) δ 10.17 (d, *J* = 6.8 Hz, 1H), 8.82 (s, 1H), 7.90 (d, *J* = 8.7 Hz, 1H), 7.43 (d, *J* = 6.8 Hz, 1H), 7.08 (m, 1H), 7.04 (dd, *J* = 8.6, 1.8 Hz, 1H), 4.19 (q, *J* = 6.8 Hz, 2H), 1.37 (t, *J* = 6.9 Hz, 3H). ^13^C NMR (100 MHz, DMSO-*d*_*6*_) δ 190.34, 163.36, 156.10, 149.78, 146.50, 132.51, 130.94, 126.98, 118.29, 113.59, 111.60, 100.97, 64.38, 14.34. HRMS-ESI: *m/z* Calcd for C_14_H_12_ClO_4_ [M + H]^+^: 279.0424; Found: 279.0417.

##### 4.1.4.3 3-Chloro-3-(2-oxo-7-propoxy-2H-chromen-3-yl)acrylaldehyde (6c)

Yellow solid. Yield: 51.2%. M. p. 150°C–152 °C. IR (KBr) ν_max_ (cm^-1^) 3069, 2969, 2880, 1725, 1662, 1615. ^1^H-NMR (400 MHz, CDCl_3_) δ 10.29 (d, *J* = 6.8 Hz, 1H), 8.47 (s, 1H), 7.68 (d, *J* = 6.7 Hz, 1H), 7.54 (d, *J* = 8.7 Hz, 1H), 6.92 (dd, *J* = 8.7, 2.3 Hz, 1H), 6.82 (d, *J* = 2.3 Hz, 1H), 4.02 (t, *J* = 6.5 Hz, 2H), 1.87 (m, 2H), 1.07 (t, *J* = 7.4 Hz, 4H). ^13^C NMR (100 MHz, CDCl_3_) δ 192.32, 164.84, 157.78, 156.13, 145.11, 144.10, 130.77, 128.13, 117.67, 114.53, 112.11, 100.70, 70.62, 22.38, 10.53. HRMS-ESI: *m/z* Calcd for C_15_H_14_ClO_4_ [M + H]^+^: 293.0581; Found: 293.0586.

##### 4.1.4.4 3-Chloro-3-(7-isopropoxy-2-oxo-2H-chromen-3-yl)acrylaldehyde (6d)

Yellow solid. Yield: 35.2%. M. p. 149°C–151°C. IR (KBr) ν_max_ (cm^-1^) 3050, 2978, 2936, 2848, 1716, 1664, 1618. ^1^H-NMR (400 MHz, CDCl_3_) δ 10.29 (d, *J* = 6.8 Hz, 1H), 8.47 (s, 1H), 7.68 (d, *J* = 6.5 Hz, 1H), 7.53 (d, *J* = 8.7 Hz, 1H), 6.88 (dd, *J* = 8.7, 2.3 Hz, 1H), 6.81 (d, *J* = 2.3 Hz, 1H), 4.65 (m, 1H), 1.40 (d, *J* = 6.0 Hz, 6H). ^13^C NMR (100 MHz, CDCl_3_) δ 192.33, 163.77, 157.82, 156.18, 145.10, 144.14, 130.85, 128.09, 117.56, 115.21, 111.93, 101.45, 71.45, 21.87 (2C). HRMS-ESI: *m/z* Calcd for C_15_H_14_ClO_4_ [M + H]^+^: 293.0581; Found: 293.0583.

##### 4.1.4.5 3-(7-(Benzyloxy)-2-oxo-2H-chromen-3-yl)-3-chloroacrylaldehyde (6e)

General procedures for the stock preparation: to a solution of phosphorus oxychloride (7.2 mL, 77.0 mmol) in DMF (30 mL), the solution of compound **4e** (20.6 g, 70.0 mmol) in DMF (12 mL) was added dropwise, and the reaction mixture was stirred at 70°C for 5 h. After completion of the reaction, the reaction mixture was diluted with saturated brine (50 mL), and then extracted with ethyl acetate (100 mL × 3). The aqueous phase was left overnight, the precipitated yellow solid was filtered off and washed with deionized water. Then the solid was dried to give crude product and the product was purified by silica gel column chromatography (Dichloromethane: Petroleum ether = 5:1) to afford the pure yellow powder **6e**. Yield: 29.2%. M. p. 223°C–225°C. IR (KBr) ν_max_ (cm^-1^) 3063, 2855, 1720, 1667, 1615. ^1^H-NMR (400 MHz, CDCl_3_) δ 10.29 (d, *J* = 6.7 Hz, 1H), 8.47 (s, 1H), 7.66 (d, *J* = 6.8 Hz, 1H), 7.55 (d, *J* = 8.7 Hz, 1H), 7.42 (m, 4H), 7.38 (m, 1H), 7.00 (dd, *J* = 8.7, 2.5 Hz, 1H), 6.90 (d, *J* = 2.4 Hz, 1H), 5.17 (s, 2H). ^13^C NMR (100 MHz, CDCl_3_) δ 192.24, 164.19, 157.68, 155.99, 144.96, 143.93, 135.34, 130.83, 128.99 (2C), 128.74, 128.29, 127.69 (2C), 118.09, 114.76, 112.45, 101.34, 70.99. HRMS-ESI: *m/z* Calcd for C_19_H_14_ClO_4_ [M + H]^+^: 341.0581; Found: 341.0588.

##### 4.1.4.6 3-Chloro-3-(7-((4-nitrobenzyl)oxy)-2-oxo-2H-chromen-3-yl)acrylaldehyde (6f)

Yellow solid. Yield: 75.2%. M. p. 250°C–252 °C. IR (KBr) ν_max_ (cm^-1^) 3072, 2923, 2850, 1721, 1669, 1620.34, 1517, 1375. ^1^H-NMR (400 MHz, DMSO-*d*_*6*_) δ 10.17 (dd, *J* = 6.8, 1.7 Hz, 1H), 8.84 (s, 1H), 8.28 (d, *J* = 8.6 Hz, 2H), 7.95 (d, *J* = 8.7 Hz, 1H), 7.76 (d, *J* = 8.5 Hz, 2H), 7.42 (d, *J* = 6.8 Hz, 1H), 7.21 (s, 1H), 7.17 (dd, *J* = 8.7, 1.9 Hz, 1H), 5.46 (s, 2H). ^13^C NMR (100 MHz, CDCl_3_) δ 188.22, 162.97, 156.49, 148.09, 145.32, 142.65, 133.01, 131.05, 130.51, 129.89, 127.93 (2C), 124.24 (2C), 114.37, 112.18, 108.58, 101.95, 69.46. HRMS-ESI: *m/z* Calcd for C_19_H_13_ClNO_6_ [M + H]^+^: 386.0431; Found: 386.0424.

##### 4.1.4.7 3-(7-((3-Bromobenzyl)oxy)-2-oxo-2H-chromen-3-yl)-3-chloroacrylaldehyde (6g)

Yellow solid. Yield: 65.9%. M. p. 217°C–219 °C. IR (KBr) ν_max_ (cm^-1^) 3053, 2868, 1718, 1663, 1616. ^1^H-NMR (400 MHz, CDCl_3_) δ 10.30 (d, *J* = 6.7 Hz, 1H), 8.49 (s, 1H), 7.68 (d, *J* = 6.7 Hz, 1H), 7.58 (d, *J* = 13.4 Hz, 2H), 7.50 (d, *J* = 7.9 Hz, 1H), 7.36 (d, *J* = 7.8 Hz, 1H), 7.30 (d, *J* = 7.9 Hz, 1H), 7.00 (d, *J* = 8.1 Hz, 1H), 6.89 (d, *J* = 8.5 Hz, 1H), 5.14 (s, 2H). ^13^C NMR (100 MHz, DMSO-*d*_*6*_) δ 192.17, 163.49, 161.35, 155.50, 154.50, 146.01, 140.25, 138.80, 131.81, 130.86, 130.63, 127.12, 127.01, 121.82, 117.35, 114.26, 112.54, 101.08, 69.22. HRMS-ESI: *m/z* Calcd for C_19_H_13_BrClO_4_ [M + H]^+^: 418.9686; Found: 418.9680.

### 4.2 Biology

#### 4.2.1 Cell culture

Non-small-cell lung cancer (A549), oral epidermoid carcinoma (KB), cervical cancer (Hela) and breast cancer (MCF-7) cells were purchased from the American Type Culture Collection (ATCC, America). Human umbilical vein endothelial cell (HUVEC) and human normal hepatocytes (LO2) cells were provided by Guangzhou Medical University (Guangzhou, China). A549 and KB cells were cultured in RMPI-1640 medium (Gibco, America). Hela, MCF-7 and LO2 cells were cultured in DMEM medium (Gibco, America). HUVEC cells were cultured in Ham’s F-12K medium (Gibco, America). All the mediums were supplemented with 10% fetal bovine serum (FBS) (BI, State of Israel), 100 U/mL penicillin and 100 μg/mL streptomycin (Gibco, America), and all the cells were incubated in a sterile incubator at 37°C in an environment of 5% CO_2_. The tested compounds were dissolved in dimethyl sulfoxide (DMSO) at a concentration of 10 mM, and then diluted into different concentration of solutions by culture medium. The final concentration of DMSO was <0.01% (v/v) in all drug treatment groups to avoid the toxicity in the biological system. Untreated cells were used as a blank control and 5-fluorouracil treated cells were used as a positive control.

#### 4.2.2 Antiproliferative assay

The incubated tumor cells were planted into 96-well plates (4×10^3^ cells/well). After 24 h, the cells were adherent to the wall and treated with the compound **6e** in different concentrations (0.78, 1.56, 3.12, 6.25, 12.50, 25.00, and 50.00 μM) for 72 h. Afterwards, the medium was supplemented with 10 µL MTT solution. After 4 h, the culture medium was removed and 100 µL DMSO was added. The absorbance was determined at the wavelength of 540 nm and 655 nm using a microplate reader (BioTek Epoch, America). The results were collected from three independent experiments. The IC_50_ was then calculated by using GraphPad Prism Software (version 7.0) and non-linear regression (curve fit).

#### 4.2.3 Scratch wound healing assay

*In vitro* wound-healing assay, KB cells (5 × 10^5^ cells/well) were seeded into 6-well plates and incubated at 37 °C in humidified atmosphere with 5% CO_2_ for 12 h. As the cells were adherent to the wall and the degree of fusion reached more than 90%, cell layers were gently scratched with a 200 μL pipette tip. After washing with Phosphate Buffered Saline (PBS), the cells were incubated with compound **6e** in different concentrations (0.5 IC_50_, IC_50_, 2 IC_50_) for 24 h. Images of cells were photographed with the inverted fluorescence microscope (Leica DMi8, Germany) at 0 h and 24 h. The areas that were not covered by cells in the scratch wound were quantified by using ImageJ. Then the values obtained in each group were expressed by the percentage of wound closure of cells in the control group. The experiments were performed in triplicate.

#### 4.2.4 Transwell migration and invasion assay

Initially, KB cells (5×10^4^ cells/well) were suspended in 200 μL of the FBS-free medium. Each upper chamber contained the vehicle and various concentrations of compound **6e** (0.5 IC_50_, IC_50_, 2 IC_50_). Each lower chamber was filled with 600 μL of medium containing 10% FBS. After incubation at 37°C for 48 h, the cells upon the transwell membrane were removed with a cotton tip. The cells trapped on the bottom side of the membrane were fixed with methanol for 1 h and stained with crystal violet solution for 2 h. Subsequently, the chambers thoroughly washed with PBS. The migrated cells were then imaged with the inverted fluorescence microscope (Leica DMi8, Germany) and counted with ImageJ software for three independent fields randomly.

For invasion assay, the transwells (8 μm pore size, Corning Incorporated) were precoated with 40 μL Matrigel for 2 h at 37°C to achieve solidification. KB cells were harvested and resuspended in serum-free medium, treated with different concentrations of compound **6e** (0.5 IC_50_, IC_50_, 2 IC_50_), and added into the upper wells of the transwell chamber at density of 1×10^5^ cells/200 μL. Then 600 μL of RPMI-1640 containing 10% FBS was added into each lower chamber. After 48 h, the invaded cells in the lower champers were fixed with paraformaldehyde for 1 h and stained with crystal violet solution for 2 h. The non-invading cells in the upper chambers of the transwell plates were scraped away by cotton swabs. Next, the chambers were washed with PBS. The invaded cells were then imaged with the inverted fluorescence microscope (Leica DMi8, Germany) and counted with ImageJ software for three independent fields randomly.

#### 4.2.5 Cell apoptosis assays

KB cells (5×10^5^ cells/well) were seeded into six-well plates. After 24 h, the cells were adherent to the wall and treated with different concentrations of compound **6e** (0.5 IC_50_, IC_50_, 2 IC_50_) for 48 h. Afterwards, cells were collected, washed twice with cold PBS. Then the treated cells were resuspended with 400 μL of 1X Annexin V binding buffer and mixed with 5 μL FITC and 5 μL PI (Annexin V-FITC Apoptosis Analysis Kit, Tianjing), and incubated at room temperature in the dark for 5 min. After the incubation, the samples were immediately analyzed by a Beckman Flow Cytometer.

#### 4.2.6 Targets prediction and network construction

The gene targets of cancers (human oral epidermoid carcinoma, cervical cancer, non-small cell lung cancer and breast cancer) were collected from GeneCards (http://www.genecards.org/). The potential targets of compound **6e** were obtained from PharmMapper (http://lilab-ecust.cn/pharmmapper/index.html), Target Net server (http://targetnet.scbdd.com/home/index/) and Swiss Target Prediction (http://www.swisstargetprediction.ch/). The last one is an online target prediction tool based on two-dimensional/three-dimensional structure similarity. UniProt knowledge database (https://www.uniprot.org/) was used to transform the targets and select *Homo sapiens* as the target species. Finally, the Draw Venn Diagram (http://bioinformatics.psb.ugent.be/Webtools/Venn/) was used to analyze the intersection of compound **6e** and cancers. The potential targets of oral cancer were identified by the Genetic Association Database (https://geneticassociationdb.nih.gov/), which is a database of genetic association data from complex diseases and disorders. Oral epidermoid carcinoma was imported as a keyword and the disease targets associated with the keyword were provided in the database.

In order to explore the relationship between the related targets of 3-(coumarin-3-yl)-acrolein and oral epidermoid carcinoma disease, protein-protein interaction (PPI) was analyzed by the Database of Interacting Proteins (DIPTM), Biological General Repository for Interaction Datasets (BioGRID), Human Protein Reference Database (HPRD), IntAct Molecular Interaction Database (IntAct), Molecular INTeraction database (MINT), and biomolecular interaction network database (BIND) with the use of the plug-in Bisogenet of Cytoscape 3.7.1 software. The PPI networks between the putative targets of 3-(coumarin-3-yl)-acrolein and the related targets of oral cancers were established and visualized by the plug-in Bisogenet of Cytoscape 3.7.1 software.

#### 4.2.7 Evaluation of MMP

MMP was determined using the JC-1 mitochondria staining Kit (Aladdin, China). KB cells were seeded into six-well plates at 6×10^5^ cells/well. After 24 h, the cells were adherent to the wall and treated with different concentrations of compound **6e** (0.5 IC_50_, IC_50_, 2 IC_50_) for 48 h. Treated KB cells were harvested and incubated with a 1X JC-1 staining solution for 30 min in the dark under 5% CO_2_ atmosphere at 37°C. Thereafter, cells were washed with 1X JC-1 staining buffer for 2 times, resuspended in 1X JC-1 staining buffer and evenly divided into two parts. On one hand, JC-1 red and green fluorescence intensities were measured with Varioskan™ LUX Multimode Microplate Reader (Thermo Fisher Scientific, America) under the fluorescence microscope. On the other hand, the other samples were tested by flow cytometry (Beckman, America). Finally, the ratio of the JC-1 with red fluorescence (aggregate form) to JC-1 with green fluorescence (monomer form) in the groups with treatment of **6e** in different concentrations was calculated.

#### 4.2.8 Western blot analysis

A total of 4 × 10^5^ KB cells were seeded into 6-well and grown for overnight. Then the cells adherent to the wall were treated with compound **6e** in different concentrations (0, 0.40, 0.80, 1.60 μM) for 24 h. After that, cells were collected, SDS lysate, 100X protease inhibitor and 50X phosphorylation inhibitor were added in a certain proportion to lyse the cells. The protein content was determined by BCA reagent (Beyotime, China) according to the standard curve. Total protein was separated with 20% SDS polyacrylamide gels for 1 h at 120 V, transferred to PVDF membranes, and blocked with rapid blocking solution for 1 hour at room temperature. After washing with 1X TTBS, PVDF membrane was incubated with the primary antibodies (1:500–1:2000) at 4°C overnight and wash for 3 times, followed by the incubation with rabbit antibody (1:2000) for 2 h at normal temperature and the wash for 3 times. Finally, the protein bands were visualized with the ECL kit and photographed using the gel imaging system (Bio-Rad, America).

#### 4.2.9 Statistical analysis

The results were represented as the mean ± SD. Variances between two groups were analyzed by One-Way ANOVA followed by *post hoc* Tukey’s test. GraphPad prism 7.0. was used to completed the data analysis. Values with a *p*-value less than 0.05 are considered as significant and statistical significance is represented as **** (*p* < 0.0001), *** (*p* < 0.001), ** (*p* < 0.01), * (*p* < 0.05), ns (not significant).

## Data Availability

The original contributions presented in the study are included in the article/Supplementary Materials, further inquiries can be directed to the corresponding author.
