# Supplementary material for: Design, synthesis and anticancer activity studies of 3-(coumarin-3-yl)-acrolein derivatives: Evidenced by integrating network pharmacology and vitro assay
Source: Front Pharmacol. 2023 Mar 23;14:1141121. doi: 10.3389/fphar.2023.1141121 (PMC10076643; doi:10.3389/fphar.2023.1141121)
Supplement: Supplementary file 1 [file DataSheet1.PDF]

# Contents

1 Spectrum Data of Intermediates ........................................ 1

1.1 Spectrum Data of Compound 2a-g ........................................ 1

1.2 Spectrum Data of Compound 4a-g ........................................ 1

2 NMR Spectra Figures of Compounds ........................................ 3

2.1 Figure S1-S2. NMR Spectra of Compound 2a ........................................ 3

2.2 Figure S3-S4. NMR Spectra of Compound 2b ........................................ 4

2.3 Figure S5-S6. NMR Spectra of Compound 2c ........................................ 5

2.4 Figure S7-S8. NMR Spectra of Compound 2d ........................................ 6

2.5 Figure S9-S10. NMR Spectra of Compound 2e ........................................ 7

2.6 Figure S11-S12. NMR Spectra of Compound 2f ........................................ 8

2.7 Figure S13-S14. NMR Spectra of Compound 2g ........................................ 9

2.8 Figure S15-S16. NMR Spectra of Compound 4a ........................................ 10

2.9 Figure S17-S18. NMR Spectra of Compound 4b ........................................ 11

2.10 Figure S19-S20. NMR Spectra of Compound 4c ........................................ 12

2.11 Figure S21-S22. NMR Spectra of Compound 4d ........................................ 13

2.12 Figure S23-S24. NMR Spectra of Compound 4e ........................................ 14

2.13 Figure S25-S26. NMR Spectra of Compound 4f ........................................ 15

2.14 Figure S27-S28. NMR Spectra of Compound 4g ........................................ 16

2.15 Figure S29-S30. NMR Spectra of Compound 5a ........................................ 17

2.16 Figure S31-S32. NMR Spectra of Compound 5b ........................................ 18

2.17 Figure S33-S34. NMR Spectra of Compound 5c ........................................ 19

2.18 Figure S35-S36. NMR Spectra of Compound 5d ........................................ 20

2.19 Figure S37-S38. NMR Spectra of Compound 5e ........................................ 21

2.20 Figure S39-S40. NMR Spectra of Compound 5f ........ 22

2.21 Figure S41-S42. NMR Spectra of Compound 5g ........ 23

2.22 Figure S43-S44. NMR Spectra of Compound 6a ........ 24

2.23 Figure S45-S46. NMR Spectra of Compound 6b ........ 25

2.24 Figure S47-S48. NMR Spectra of Compound 6c ........ 26

2.25 Figure S49-S50. NMR Spectra of Compound 6d ........ 27

2.26 Figure S51-S52. NMR Spectra of Compound 6e ........ 28

2.27 Figure S53-S54. NMR Spectra of Compound 6f ........ 29

2.28 Figure S55-S56. NMR Spectra of Compound 6g ........ 30

3 Network Construction and Targets Prediction ........ 31

# *Supplementary Material*

## 1 Spectrum Data of Intermediates

### 1.1 Spectrum Data of Compound 2a-g

**3-Acetyl-2*H*-chromen-2-one** (**2a**). White solid, yield 91.1 %. $^{1}$H-NMR (400 MHz, $CDCl_3$) δ 8.51 (s, 1H), 7.65 (ddd, *J* = 7.3, 4.2, 2.6 Hz, 2H), 7.38 – 7.32 (m, 2H), 2.73 (s, 3H). $^{13}$C NMR (100 MHz, $CDCl_3$) δ 195.64, 159.37, 155.45, 147.61, 134.53, 130.36, 125.11, 124.64, 118.38, 116.82, 30.69.

**3-Acetyl-7-hydroxy-2*H*-chromen-2-one** (**2b**). Yellowish white solid, yield 94.0%. $^{1}$H-NMR (400 MHz, DMSO-$d_6$) δ 8.40 (s, 1H), 7.55 (d, *J* = 8.8 Hz, 1H), 6.54 (dd, *J* = 8.8, 2.1 Hz, 1H), 6.34 (d, *J* = 1.9 Hz, 1H), 2.49 (s, 3H). $^{13}$C NMR (100 MHz, DMSO-$d_6$) δ 194.21, 171.55, 160.01, 158.70, 147.17, 132.54, 117.39, 114.12, 108.52, 102.24, 30.12.

**3-Acetyl-7-(diethylamino)-2*H*-chromen-2-one** (**2c**). Yellow solid, yield 92.0 %. $^{1}$H-NMR (400 MHz, $CDCl_3$) δ 8.43 (s, 1H), 7.39 (d, *J* = 9.0 Hz, 1H), 6.61 (dd, *J* = 9.0, 2.5 Hz, 1H), 6.46 (d, *J* = 2.5 Hz, 1H), 3.45 (q, *J* = 7.2 Hz, 4H), 2.68 (s, 3H), 1.24 (t, *J* = 7.2 Hz, 6H). $^{13}$C NMR (100 MHz, $CDCl_3$) δ 195.88, 161.01, 158.86, 153.12, 148.00, 132.02, 116.18, 109.96, 108.26, 96.66, 45.26 (2C), 30.74, 12.55 (2C).

**3-Acetyl-7-bromo-2*H*-chromen-2-one** (**2d**). Yellow solid, yield 56.2 %. $^{1}$H-NMR (400 MHz, $CDCl_3$) δ 8.46 (d, *J* = 3.9 Hz, 1H), 7.56 (s, 1H), 7.49 (m, 2H), 2.71 (d, *J* = 3.7 Hz, 3H). $^{13}$C NMR (100 MHz, $CDCl_3$) δ 195.24, 158.66, 155.48, 146.88, 131.13, 129.13, 128.74, 124.58, 120.19, 117.25, 30.68.

**3-Acetyl-6-hydroxy-2*H*-chromen-2-one** (**2e**). Yellow solid, yield 90.1 %. $^{1}$H-NMR (400 MHz, DMSO-$d_6$) δ 9.91 (s, 1H), 8.55 (s, 1H), 7.29 (d, *J* = 9.0 Hz, 1H), 7.22 (d, *J* = 3.0 Hz, 1H), 7.15 (dd, *J* = 8.8, 2.9 Hz, 1H), 2.56 (s, 3H). $^{13}$C NMR (100 MHz, DMSO-$d_6$) δ 195.40, 158.76, 154.06, 148.09, 147.10, 124.43, 122.80, 118.71, 117.09, 114.21, 30.16.

**3-Acetyl-6-methyl-2*H*-chromen-2-one** (**2f**). Yellow solid, yield 48.5 %. $^{1}$H-NMR (400 MHz, $CDCl_3$) δ 8.29 (s, 1H), 7.29 (dd, *J* = 8.3, 2.1 Hz, 1H), 7.26 (s, 1H), 7.10 (d, *J* = 8.3 Hz, 1H), 2.56 (s, 3H), 2.26 (s, 3H). $^{13}$C NMR (100 MHz, $CDCl_3$) δ 195.78, 159.58, 153.58, 147.64, 135.74, 134.93, 129.91, 124.36, 118.07, 116.47, 30.71, 20.80.

**3-Acetyl-6-bromo-2*H*-chromen-2-one** (**2g**). Yellow solid, yield 66.6 %. $^{1}$H-NMR (400 MHz, $CDCl_3$) δ 8.39 (s, 1H), 7.76 (d, *J* = 2.2 Hz, 1H), 7.71 (dd, *J* = 8.7, 2.3 Hz, 1H), 7.24 (d, *J* = 1.7 Hz, 1H), 2.70 (s, 3H). $^{13}$C NMR (100 MHz, $CDCl_3$) δ 195.24, 158.73, 154.21, 146.20, 137.21, 132.34, 125.51, 119.84, 118.58, 117.68, 30.72.

### 1.2 Spectrum Data of Compound 4a-g

**3-Acetyl-7-methoxy-2*H*-chromen-2-one** (**4a**). Yellow solid, yield: 78.8 %. $^{1}$H-NMR (400 MHz, $CDCl_3$) δ 8.50 (d, *J* = 3.8 Hz, 1H), 7.55 (dd, *J* = 8.6, 3.7 Hz, 1H), 6.90 (d, *J* = 8.5 Hz, 1H), 6.83

(s, 1H), 3.92 (d, $J$ = 3.8 Hz, 3H), 2.71 (d, $J$ = 3.7 Hz, 3H). $^{13}$C NMR (100 MHz, $CDCl_3$) δ 195.65, 165.38, 159.86, 157.90, 147.93, 131.62, 120.73, 114.00, 112.14, 100.38, 56.16, 30.71.

**3-Acetyl-7-ethoxy-2*H*-chromen-2-one** (**4b**). Yellow powder, yield: 80.1 %. $^{1}$H-NMR (400 MHz, $CDCl_3$) δ 8.50 (d, $J$ = 3.6 Hz, 1H), 7.54 (dd, $J$ = 8.7, 3.6 Hz, 1H), 6.88 (dt, $J$ = 8.7, 3.0 Hz, 1H), 6.80 (d, $J$ = 2.7 Hz, 1H), 4.13 (tt, $J$ = 7.3, 3.4 Hz, 2H), 2.71 (d, $J$ = 3.8 Hz, 3H), 1.48 (td, $J$ = 6.8, 3.6 Hz, 3H). $^{13}$C NMR (100 MHz, $CDCl_3$) δ 195.69, 164.83, 159.95, 157.92, 147.99, 131.61, 120.55, 114.34, 111.99, 100.78, 64.70, 30.72, 14.58.

**3-Acetyl-7-propoxy-2*H*-chromen-2-one** (**4c**). Yellow powder, yield: 87.7 %. $^{1}$H-NMR (400 MHz, $CDCl_3$) δ 8.48 (s, 1H), 7.52 (d, $J$ = 8.7 Hz, 1H), 6.88 (dd, $J$ = 8.7, 2.6 Hz, 1H), 6.80 (d, $J$ = 2.4 Hz, 1H), 4.00 (t, $J$ = 6.5 Hz, 2H), 2.69 (s, 3H), 1.85 (h, $J$ = 7.2 Hz, 2H), 1.05 (t, $J$ = 7.4 Hz, 3H). $^{13}$C NMR (100 MHz, $CDCl_3$) δ 195.75, 165.05, 160.00, 157.95, 148.05, 131.60, 120.50, 114.40, 111.97, 100.80, 70.56, 30.76, 22.37, 10.54.

**3-Acetyl-7-isopropoxy-2*H*-chromen-2-one** (**4d**). Yellow powder, yield: 65.2 %. $^{1}$H-NMR (400 MHz, $CDCl_3$) δ 8.49 (s, 1H), 7.52 (d, $J$ = 8.7 Hz, 1H), 6.85 (dd, $J$ = 8.7, 2.3 Hz, 1H), 6.79 (d, $J$ = 2.4 Hz, 1H), 4.65 (p, $J$ = 6.0 Hz, 1H), 2.70 (s, 3H), 1.39 (d, $J$ = 6.2 Hz, 6H). $^{13}$C NMR (100 MHz, $CDCl_3$) δ 195.75, 163.97, 160.03, 157.99, 148.04, 131.70, 120.36, 115.06, 111.79, 101.53, 71.37, 30.75, 21.85 (2C).

**3-Acetyl-7-(benzyloxy)-2*H*-chromen-2-one** (**4e**). Yellow solid, yield: 98.5 %. $^{1}$H-NMR (400 MHz, DMSO-$d_6$) δ 8.64 (s, 1H), 7.88 (d, $J$ = 8.8 Hz, 1H), 7.49 (d, $J$ = 8.5 Hz, 2H), 7.44 – 7.34 (m, 3H), 7.15 (d, $J$ = 2.3 Hz, 1H), 7.09 (dd, $J$ = 8.7, 2.4 Hz, 1H), 5.26 (s, 2H), 2.56 (s, 3H). $^{13}$C NMR (100 MHz, $CDCl_3$) δ 195.64, 164.40, 159.85, 157.79, 147.87, 135.38, 131.66, 128.97 (2C), 128.72, 127.68 (2C), 120.89, 114.59, 112.33, 101.45, 70.93, 30.72.

**3-Acetyl-7-((4-nitrobenzyl) oxy)-2*H*-chromen-2-one** (**4f**). Yellow powder, yield: 52.5 %. $^{1}$H-NMR (400 MHz, $CDCl_3$) δ 8.51 (s, 1H), 8.29 (d, $J$ = 8.7 Hz, 2H), 7.61 (dd, $J$ = 12.0, 8.5 Hz, 3H), 6.99 (dd, $J$ = 8.8, 2.3 Hz, 1H), 6.88 (d, $J$ = 2.3 Hz, 1H), 5.27 (s, 2H), 2.70 (s, 3H). $^{13}$C NMR (100 MHz, $CDCl_3$) 195.58, 165.95, 159.65, 150.25, 147.73, 142.73, 137.65, 131.91, 127.93 (2C), 124.23 (2C), 114.32, 112.79, 109.16, 101.47, 69.42, 30.75.

**3-Acetyl-7-((3-bromobenzyl) oxy)-2*H*-chromen-2-one** (**4g**). Yellow powder, yield: 95.7 %. $^{1}$H-NMR (400 MHz, $CDCl_3$) δ 8.49 (s, 1H), 7.59 (s, 1H), 7.56 (d, $J$ = 8.7 Hz, 1H), 7.50 (d, $J$ = 7.7 Hz, 1H), 7.35 (d, $J$ = 7.8 Hz, 1H), 7.29 (d, $J$ = 7.8 Hz, 1H), 6.97 (dd, $J$ = 8.7, 2.3 Hz, 1H), 6.87 (d, $J$ = 2.3 Hz, 1H), 5.13 (s, 2H), 2.70 (s, 3H). $^{13}$C NMR (100 MHz, $CDCl_3$) δ 195.62, 163.97, 159.78, 157.75, 147.82, 137.68, 131.79 (2C), 130.54 (2C), 126.06, 123.06, 121.11, 114.48, 112.54, 101.42, 69.91, 30.73.

## 2 NMR Spectra Figures of Compounds

### 2.1 Figure S1-S2. NMR Spectra of Compound 2a

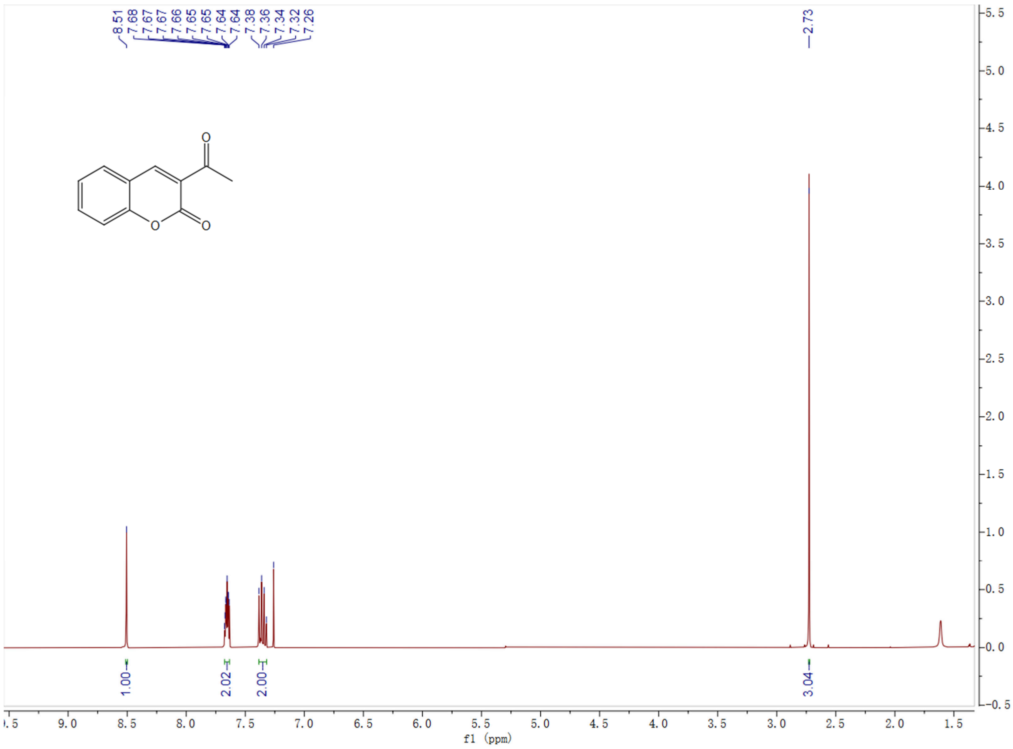

**Figure S1.** $^1$H NMR Spectrum (400 MHz, $CDCl_3$) of Compound **2a**

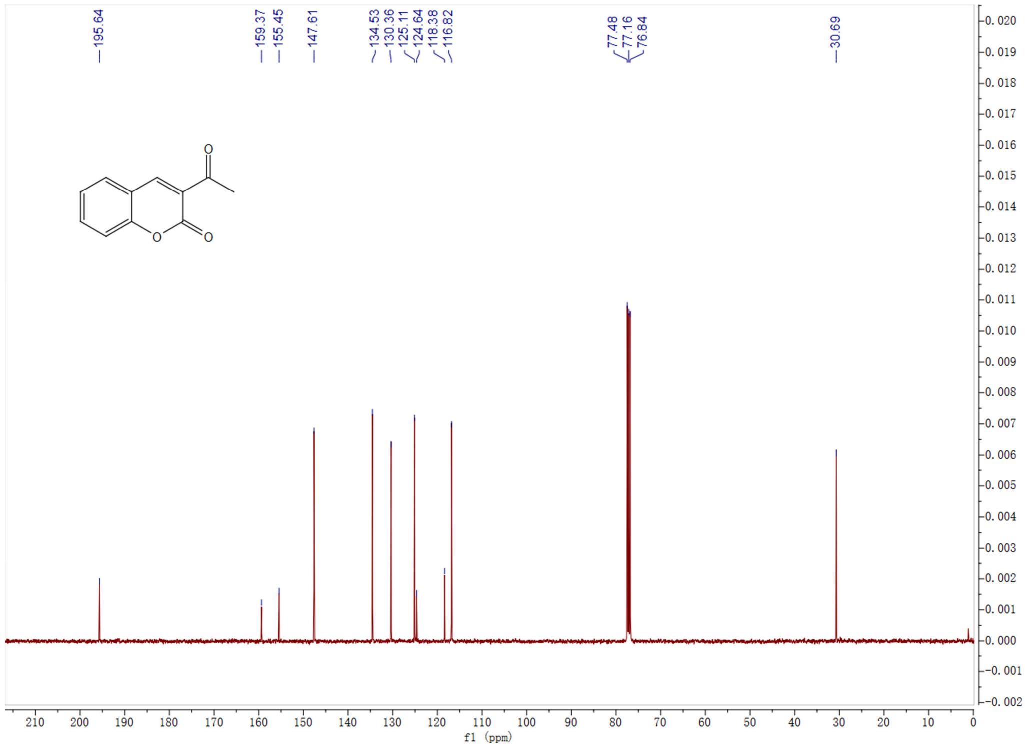

**Figure S2.** $^{13}$C NMR Spectrum (100 MHz, $CDCl_3$) of Compound **2a**

## 2.2 Figure S3-S4. NMR Spectra of Compound 2b

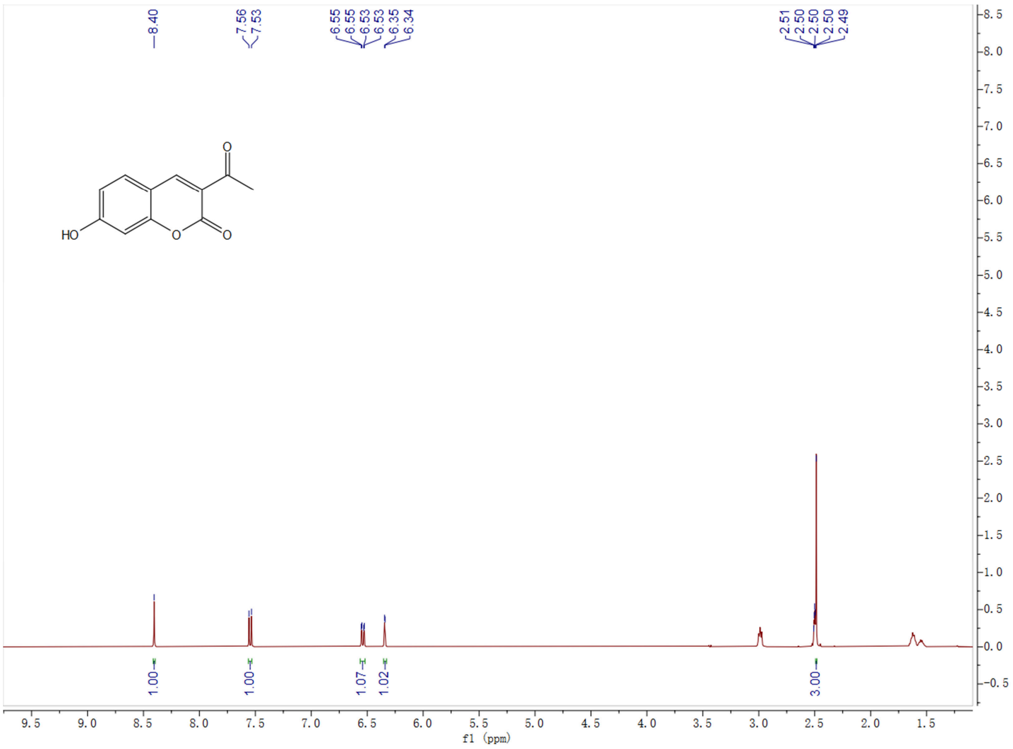

**Figure S3.** $^{1}H$ NMR Spectrum (400 MHz, DMSO-$d_6$) of Compound **2b**

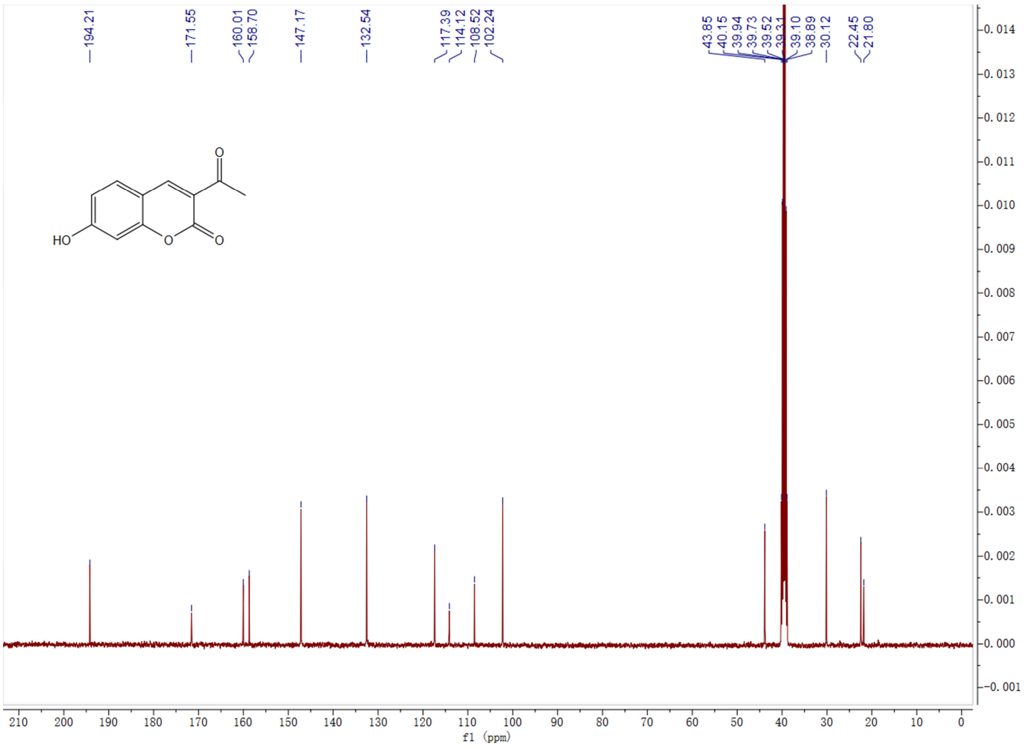

**Figure S4.** $^{13}C$ NMR Spectrum (100 MHz, $CDCl_3$) of Compound **2b**

## 2.3 Figure S5-S6. NMR Spectra of Compound 2c

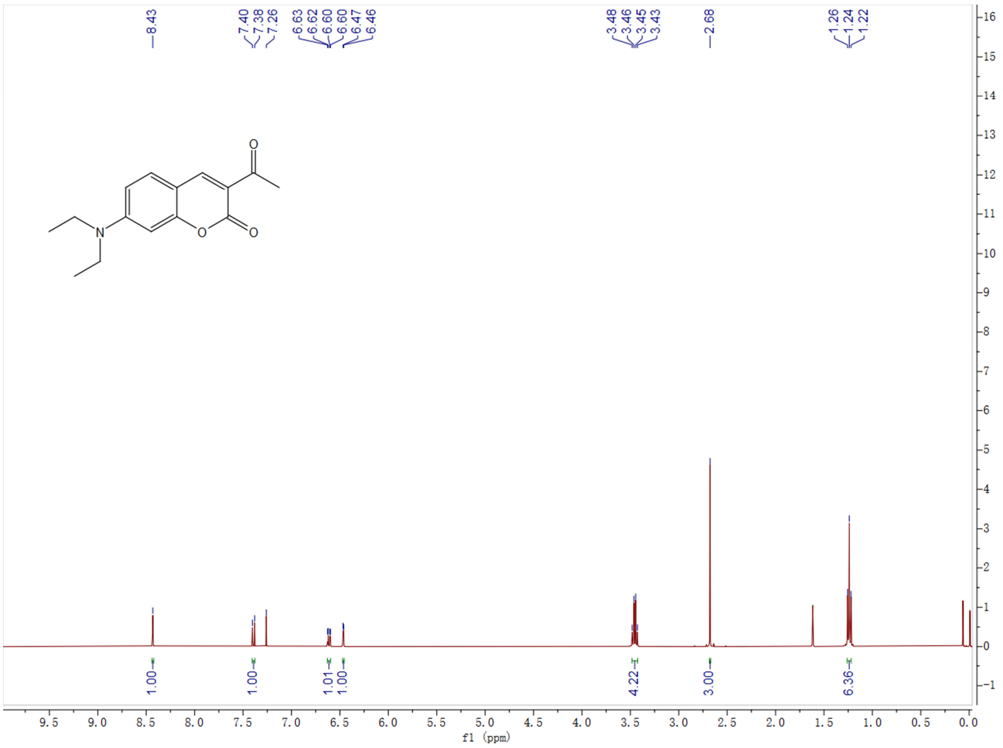


**Figure S5.** $^1$H NMR Spectrum (400 MHz, $CDCl_3$) of Compound **2c**

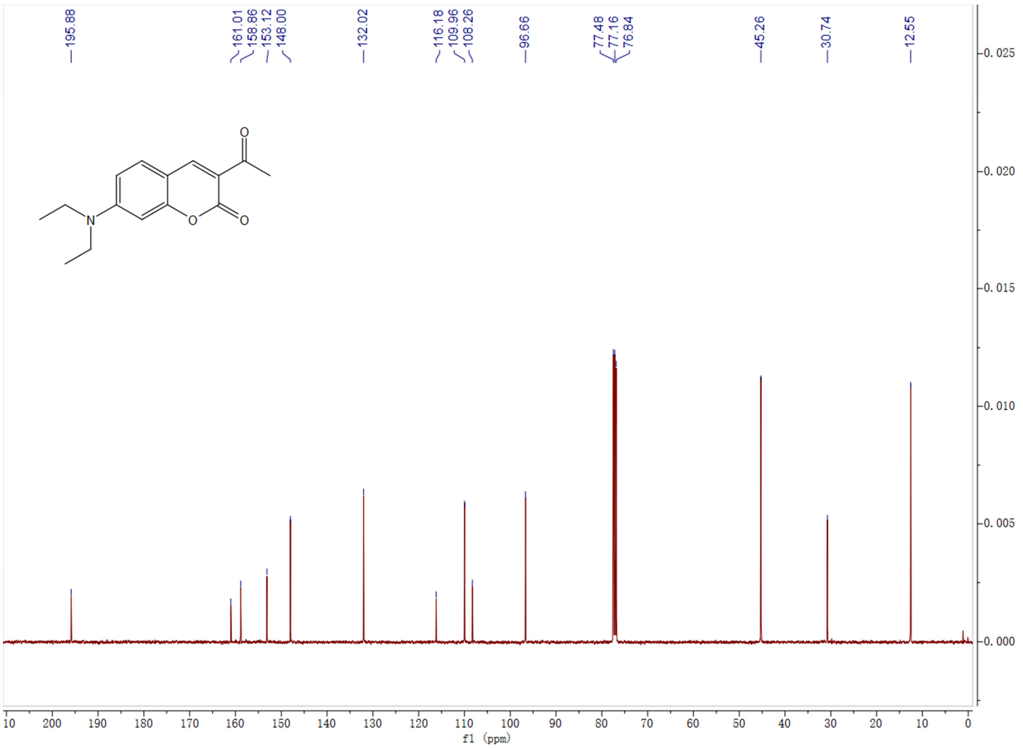


**Figure S6.** $^{13}$C NMR Spectrum (100 MHz, $CDCl_3$) of Compound **2c**

## 2.4 Figure S7-S8. NMR Spectra of Compound 2d

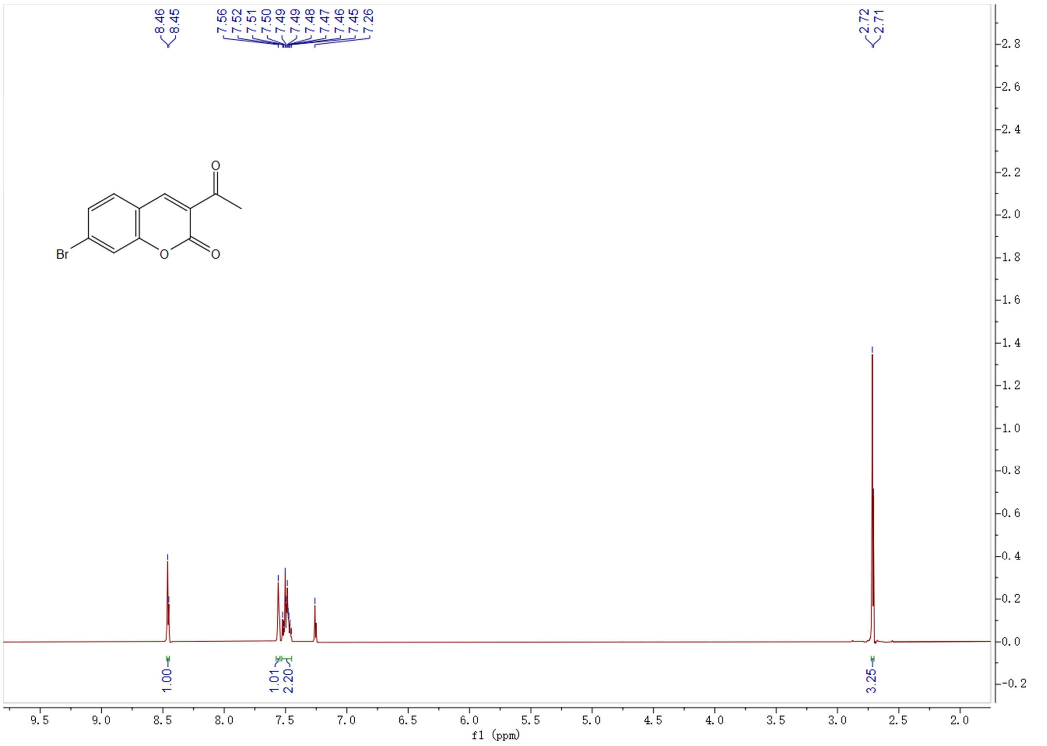

**Figure S7.** $^{1}H$ NMR Spectrum (400 MHz, $CDCl_3$) of Compound **2d**

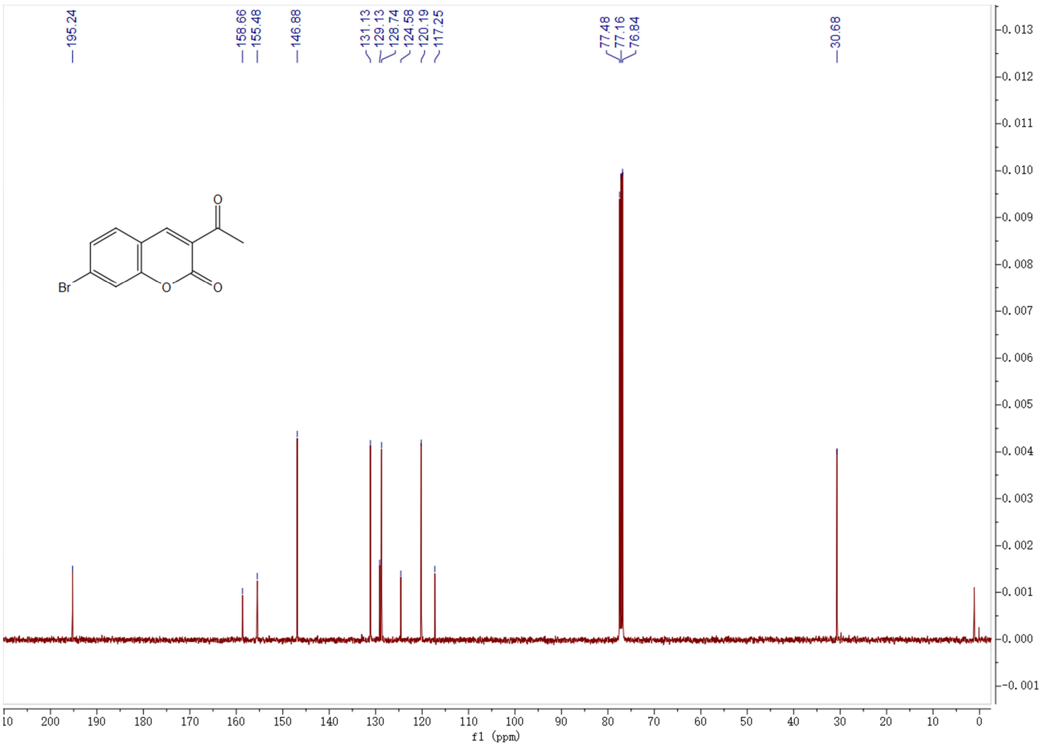

**Figure S8.** $^{13}C$ NMR Spectrum (100 MHz, $CDCl_3$) of Compound **2d**

## 2.5 Figure S9-S10. NMR Spectra of Compound 2e

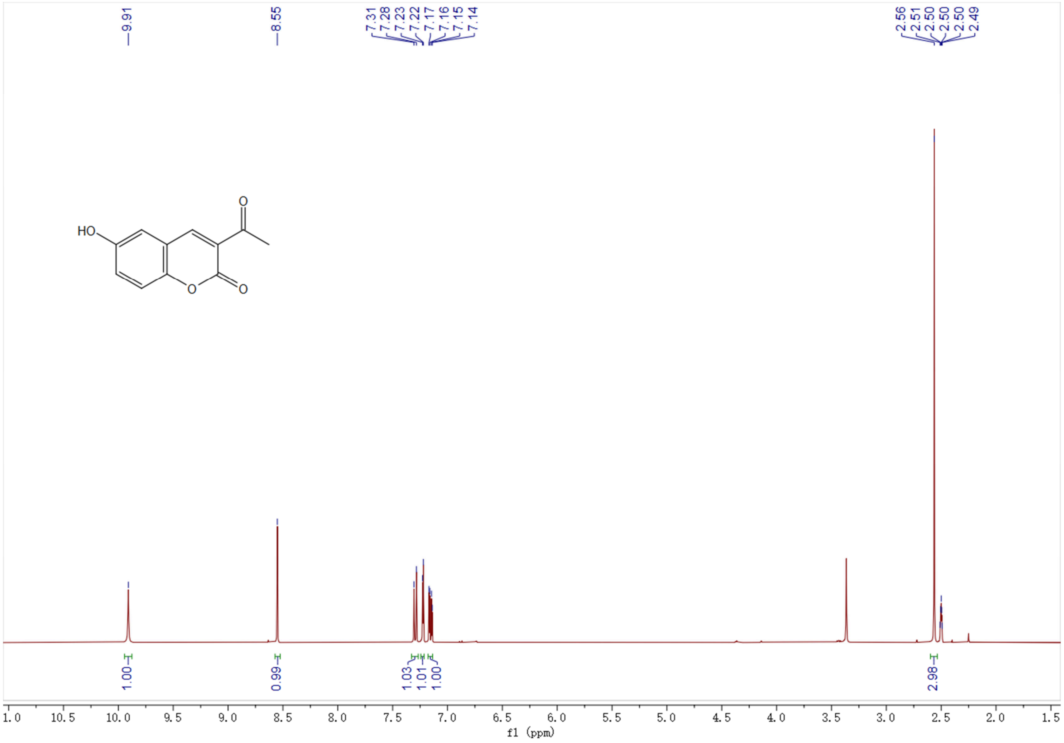


**Figure S9.** $^{1}$H NMR Spectrum (400 MHz, DMSO-$d_6$) of Compound **2e**

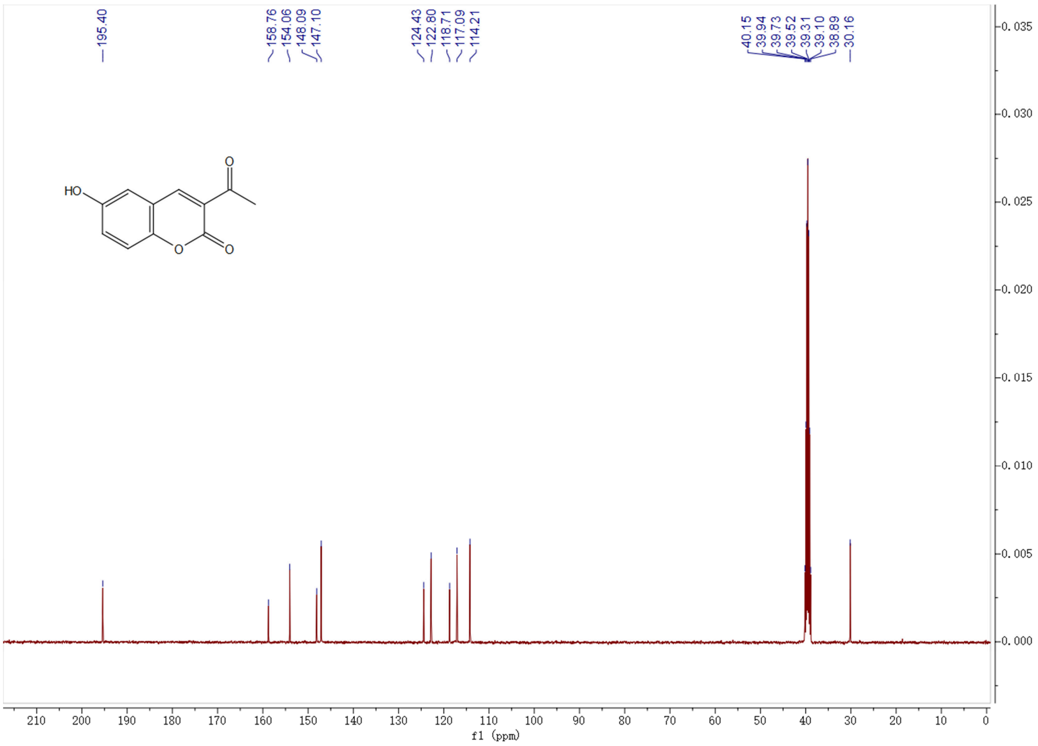


**Figure S10.** $^{13}$C NMR Spectrum (100 MHz, DMSO-$d_6$) of Compound **2e**

## 2.6 Figure S11-S12. NMR Spectra of Compound 2f

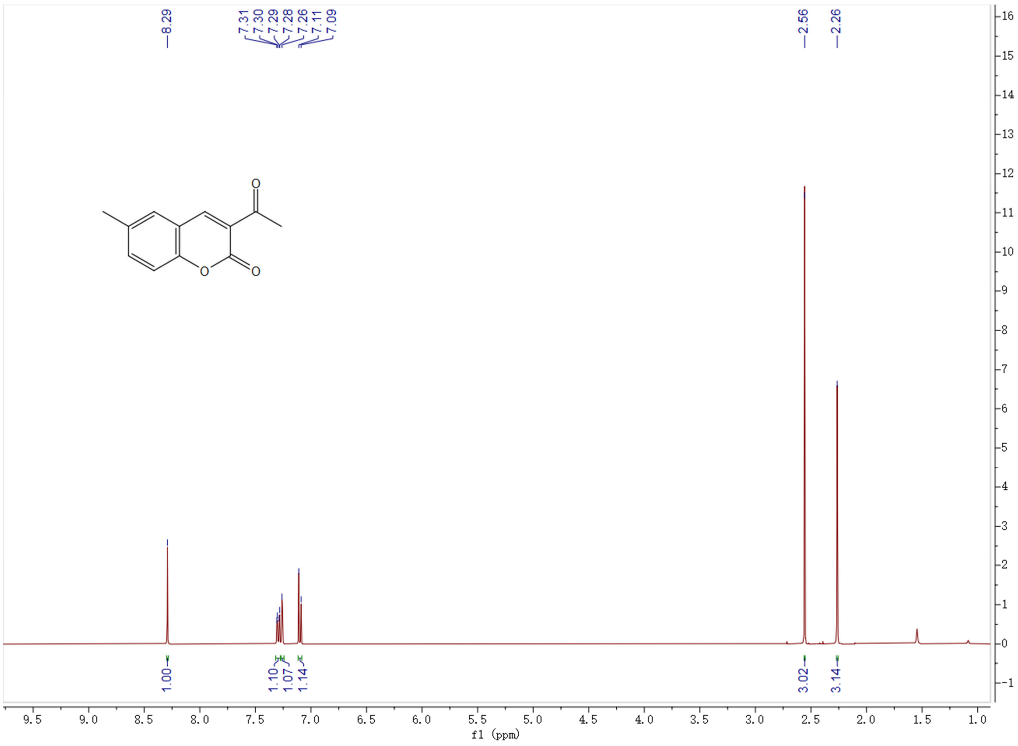


**Figure S11.** $^{1}$H NMR Spectrum (400 MHz, $CDCl_3$) of Compound **2f**

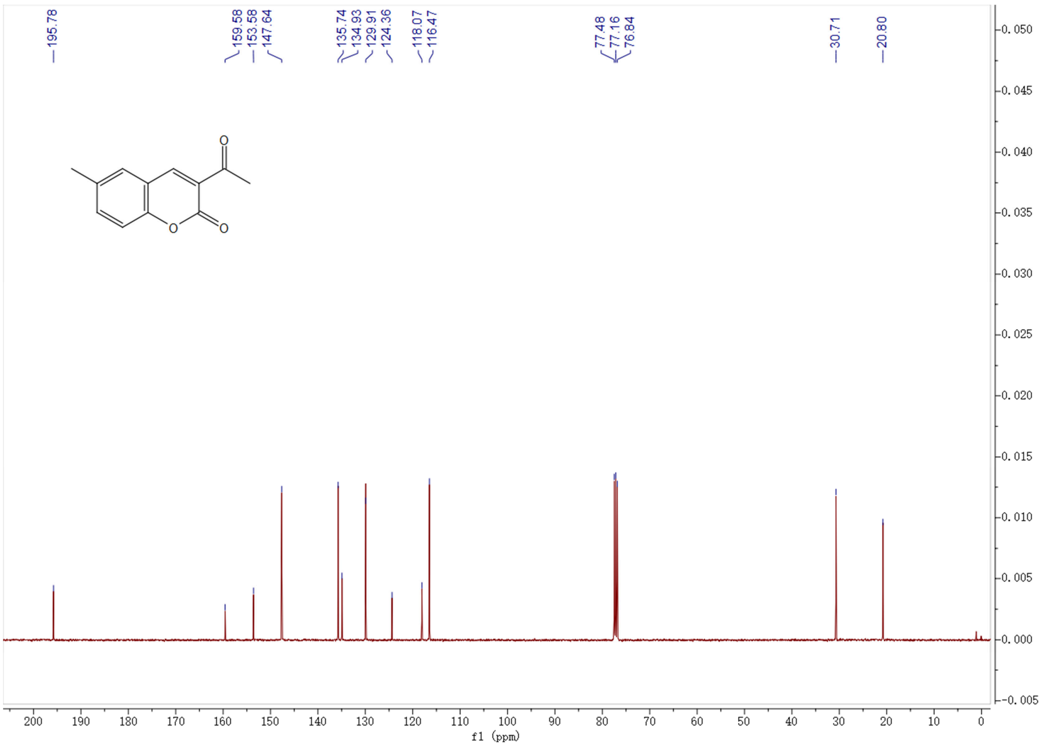


**Figure S12.** $^{13}$C NMR Spectrum (100 MHz, $CDCl_3$) of Compound **2f**

## 2.7 Figure S13-S14. NMR Spectra of Compound 2g

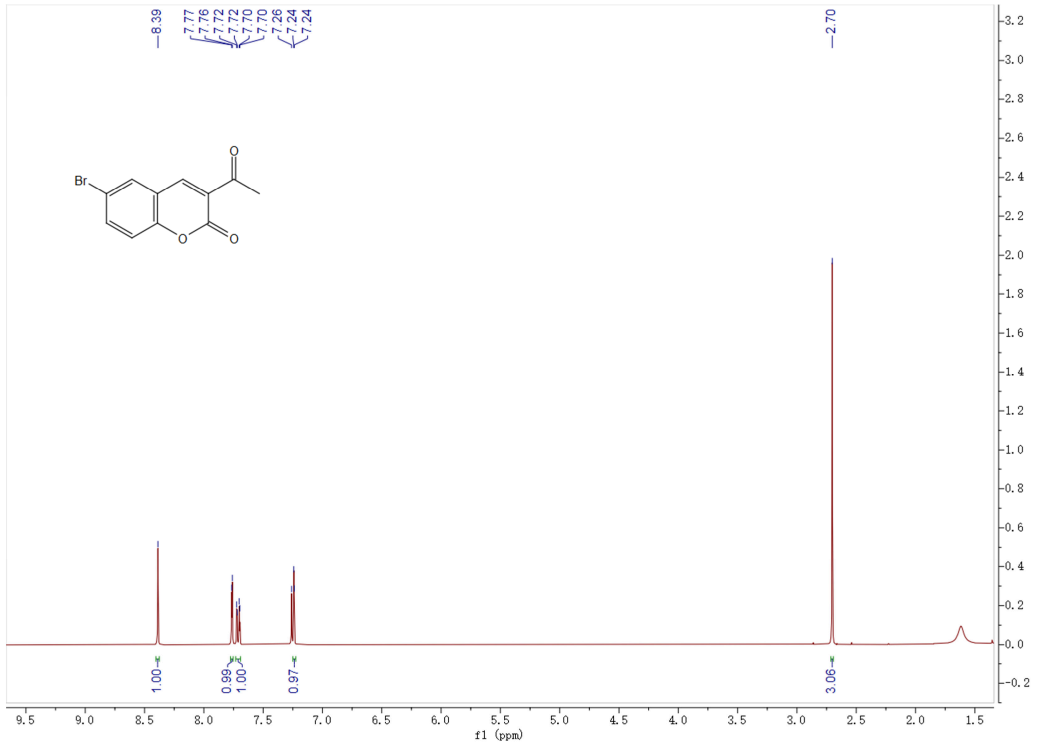

**Figure S13.** $^{1}H$ NMR Spectrum (400 MHz, $CDCl_3$) of Compound **2g**

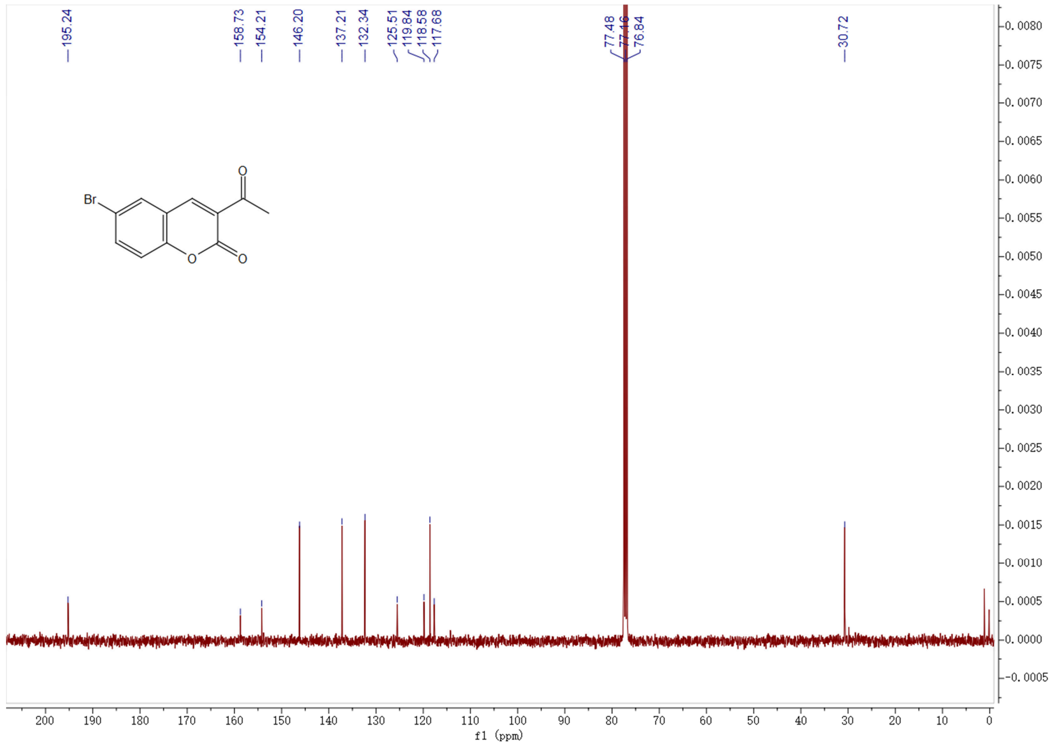

**Figure S14.** $^{13}C$ NMR Spectrum (100 MHz, $CDCl_3$) of Compound **2g**

## 2.8 Figure S15-S16. NMR Spectra of Compound 4a

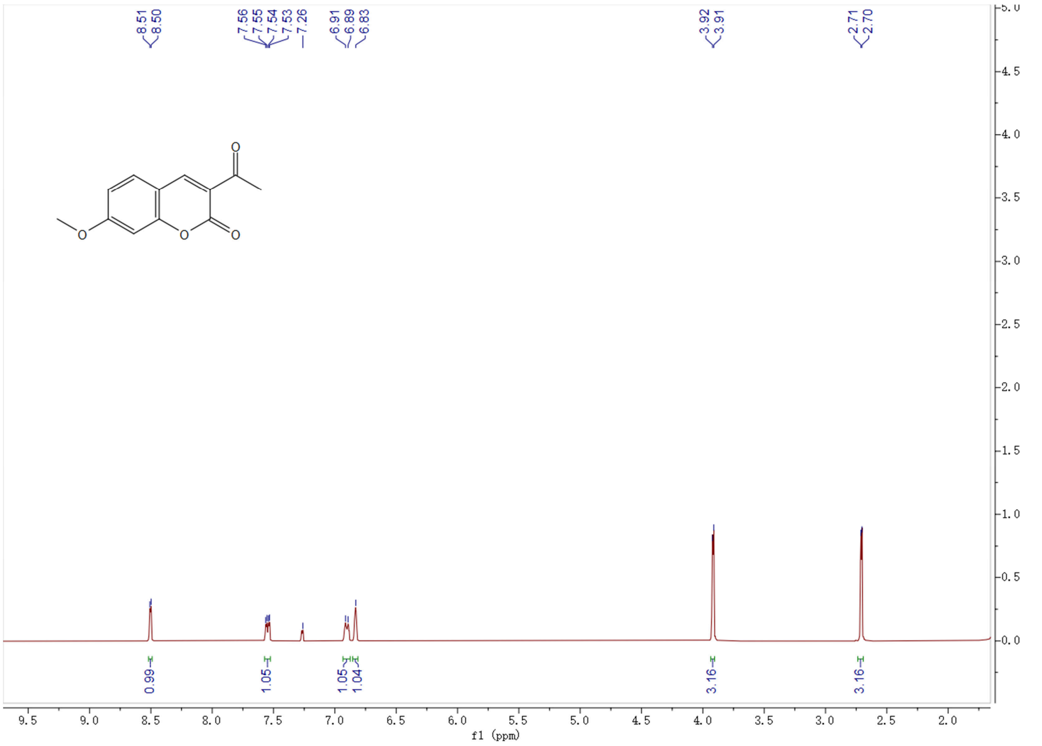


**Figure S15.** $^{1}$H NMR Spectrum (400 MHz, $CDCl_3$) of Compound **4a**

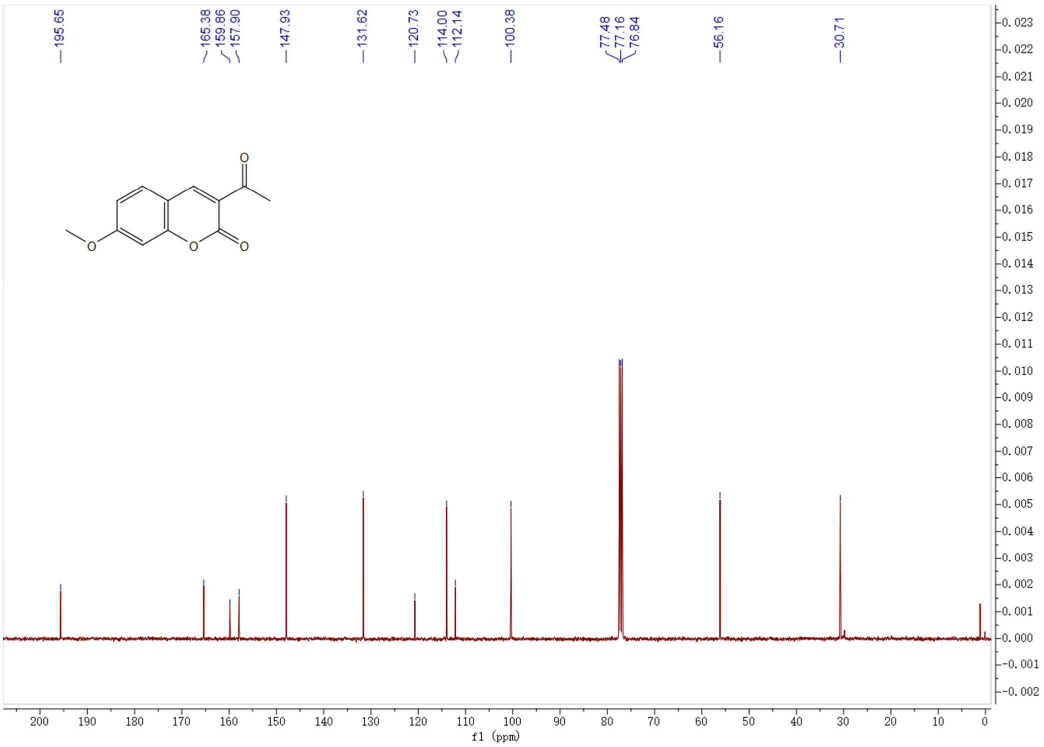


**Figure S16.** $^{13}$C NMR Spectrum (100 MHz, $CDCl_3$) of Compound **4a**

## 2.9 Figure S17-S18. NMR Spectra of Compound 4b

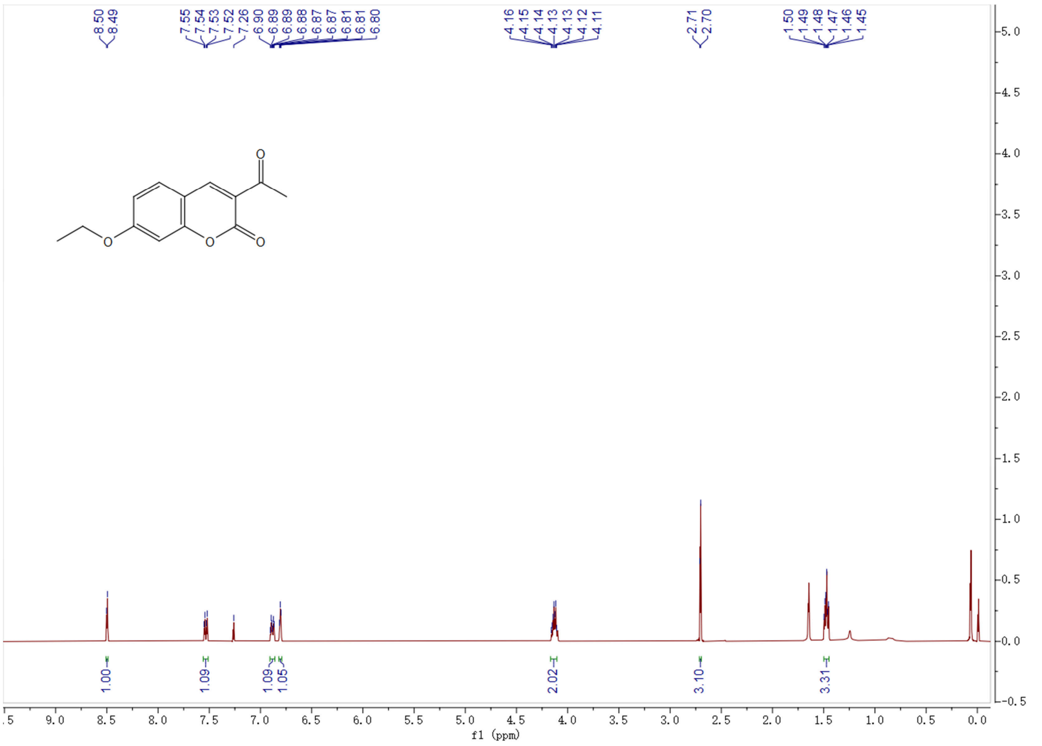

**Figure S17.** $^{1}H$ NMR Spectrum (400 MHz, $CDCl_3$) of Compound **4b**

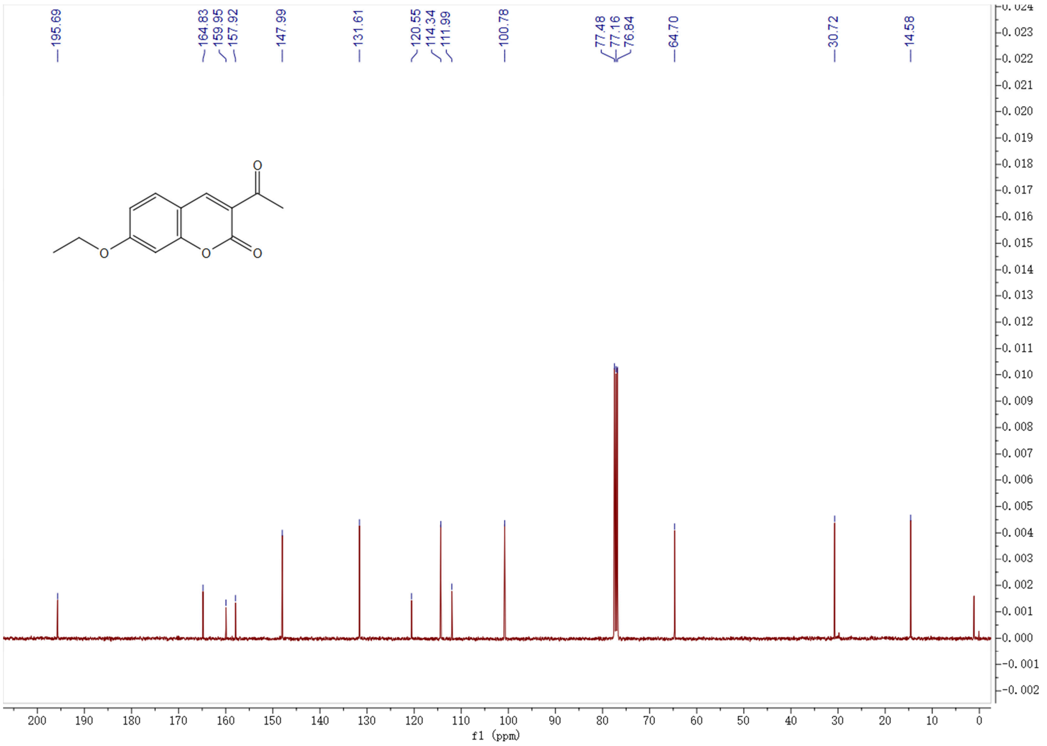

**Figure S18.** $^{13}C$ NMR Spectrum (100 MHz, $CDCl_3$) of Compound **4b**

## 2.10 Figure S19-S20. NMR Spectra of Compound 4c

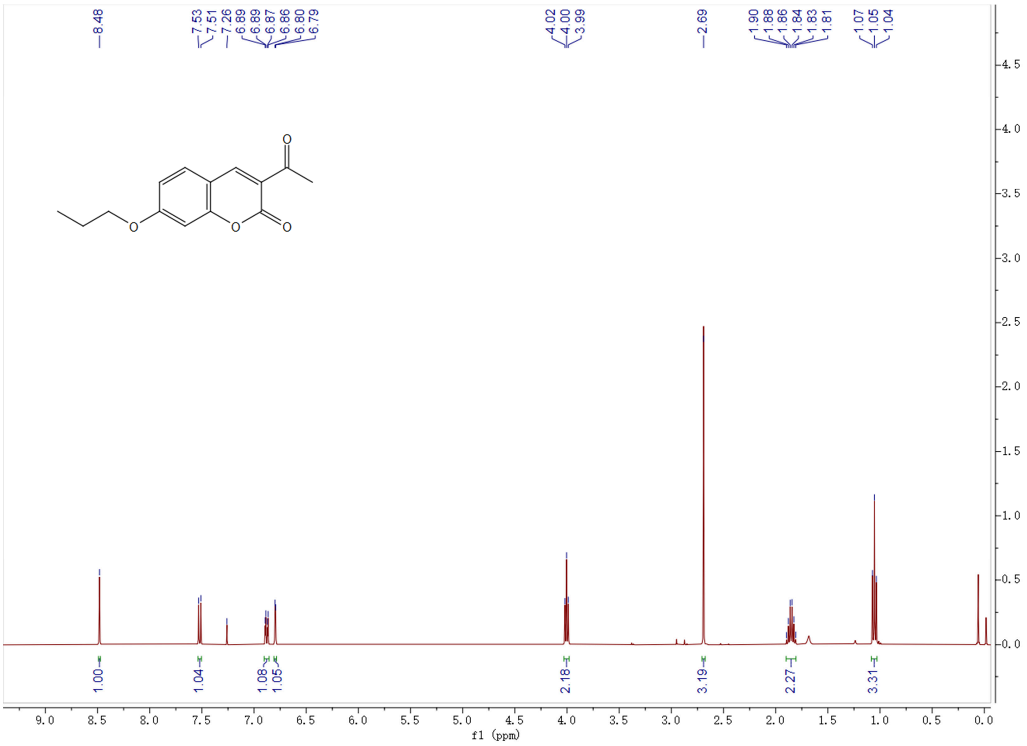

**Figure S19.** $^{1}H$ NMR Spectrum (400 MHz, $CDCl_3$) of Compound **4c**

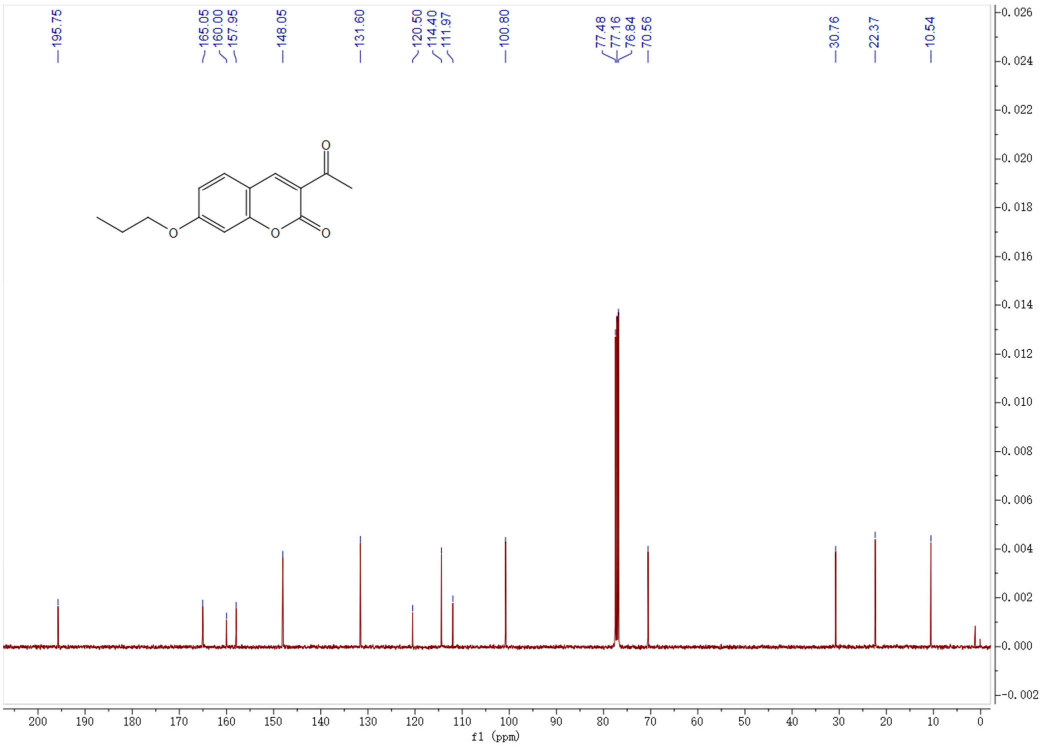

**Figure S20.** $^{13}C$ NMR Spectrum (100 MHz, $CDCl_3$) of Compound **4c**

## 2.11 Figure S21-S22. NMR Spectra of Compound 4d

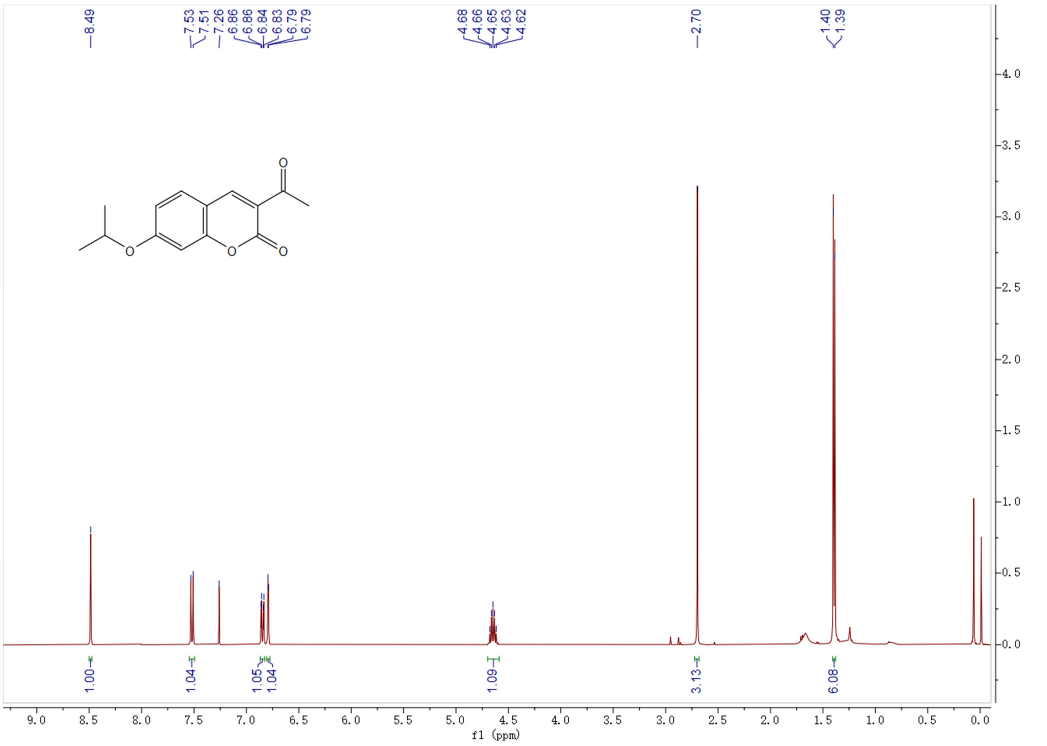

**Figure S21.** $^{1}$H NMR Spectrum (400 MHz, $CDCl_3$) of Compound **4d**

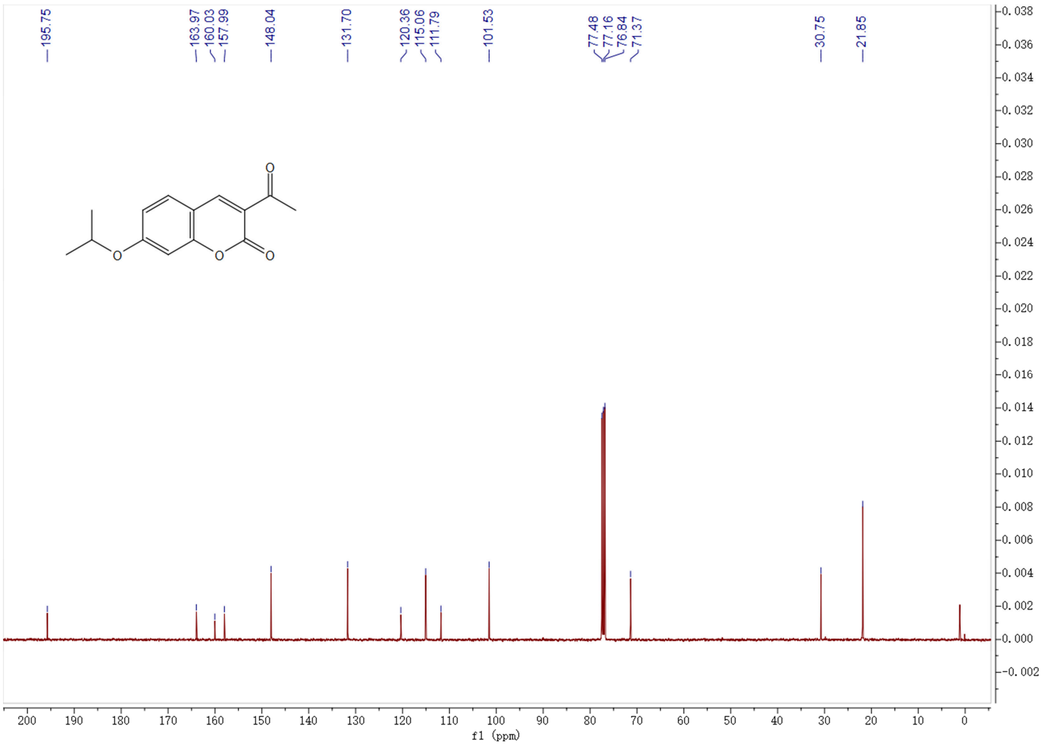

**Figure S22.** $^{13}$C NMR Spectrum (100 MHz, $CDCl_3$) of Compound **4d**

## 2.12 Figure S23-S24. NMR Spectra of Compound 4e

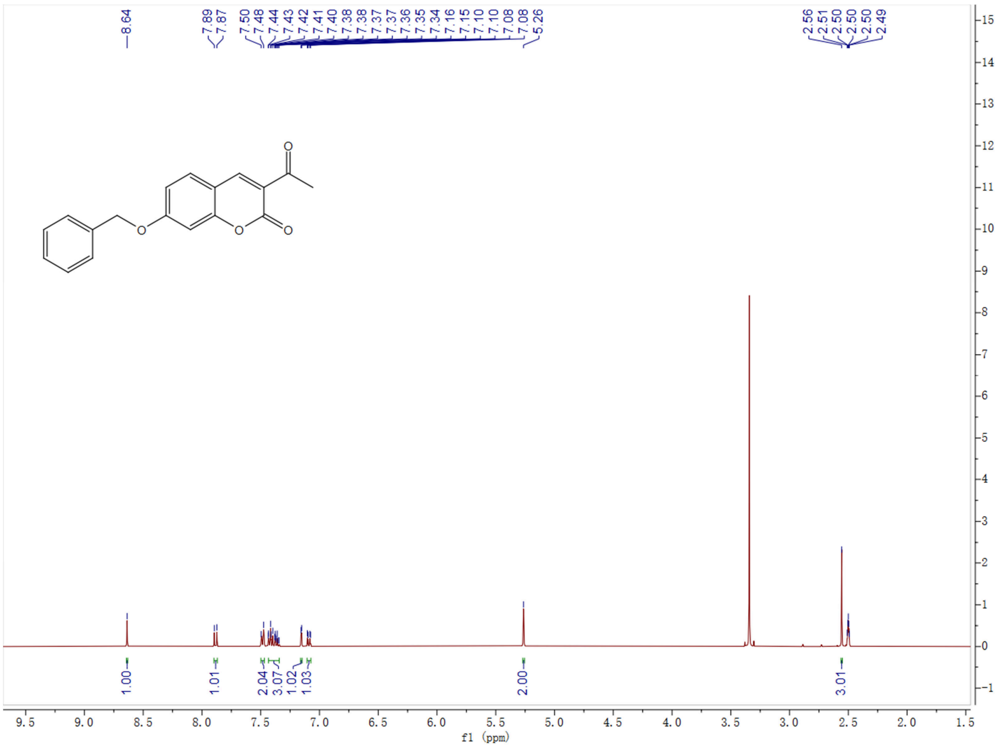

**Figure S23.** $^{1}$H NMR Spectrum (400 MHz, DMSO-$d_6$) of Compound **4e**

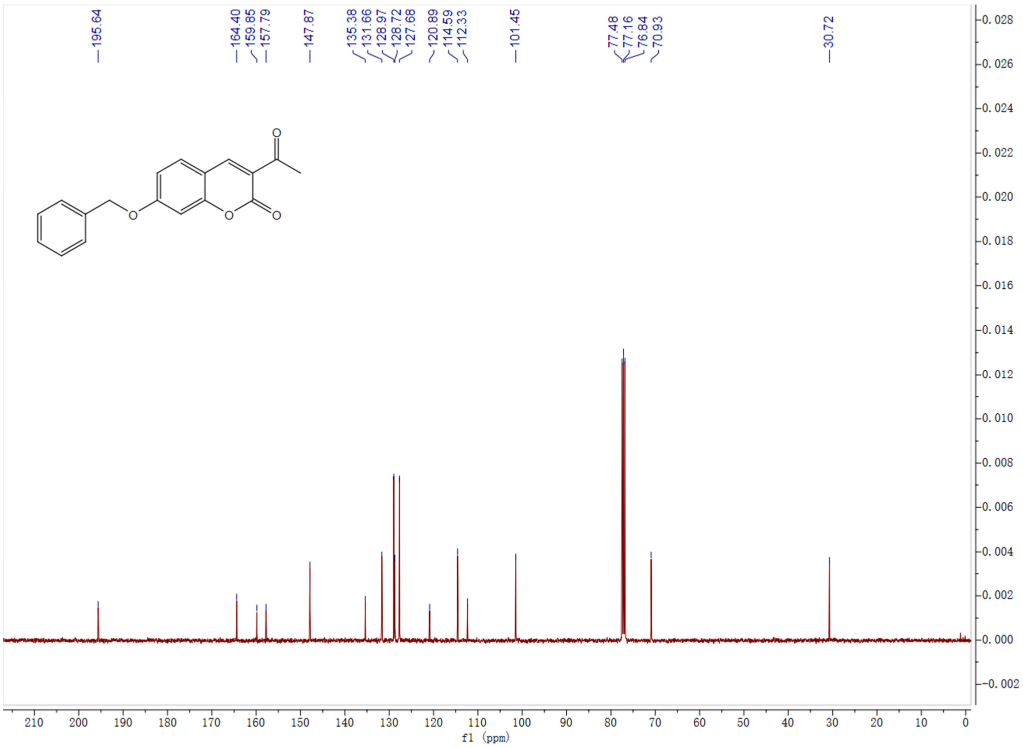

**Figure S24.** $^{13}$C NMR Spectrum (100 MHz, $CDCl_3$) of Compound **4e**

## 2.13 Figure S25-S26. NMR Spectra of Compound 4f

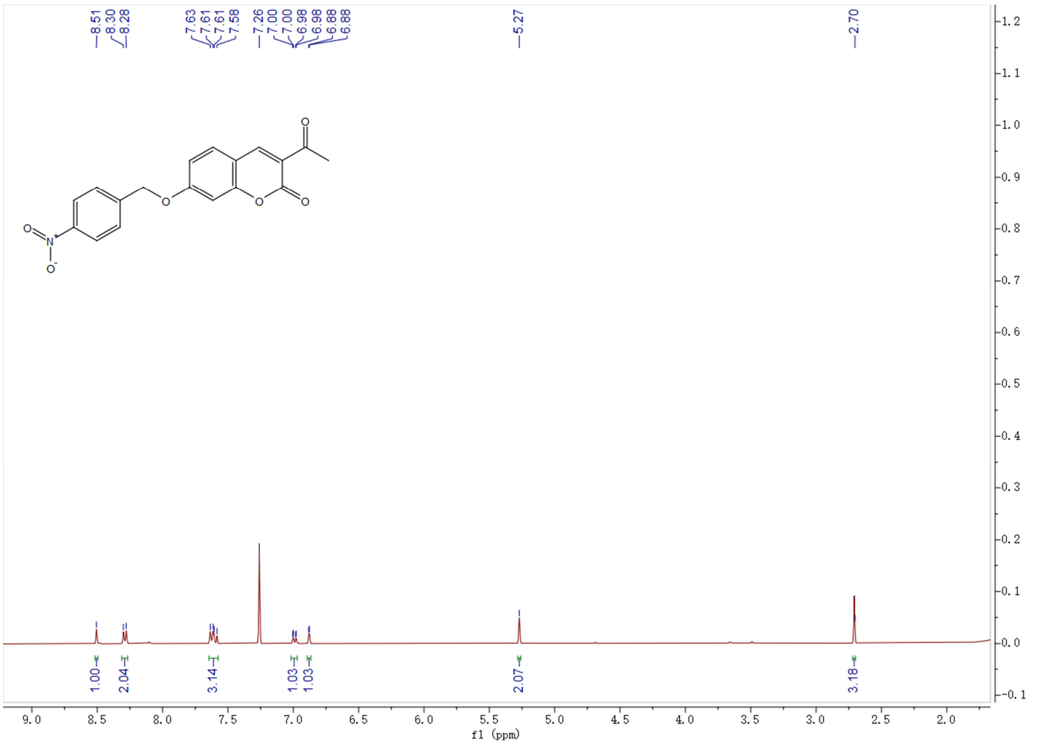

**Figure S25.** $^{1}H$ NMR Spectrum (400 MHz, $CDCl_3$) of Compound **4f**

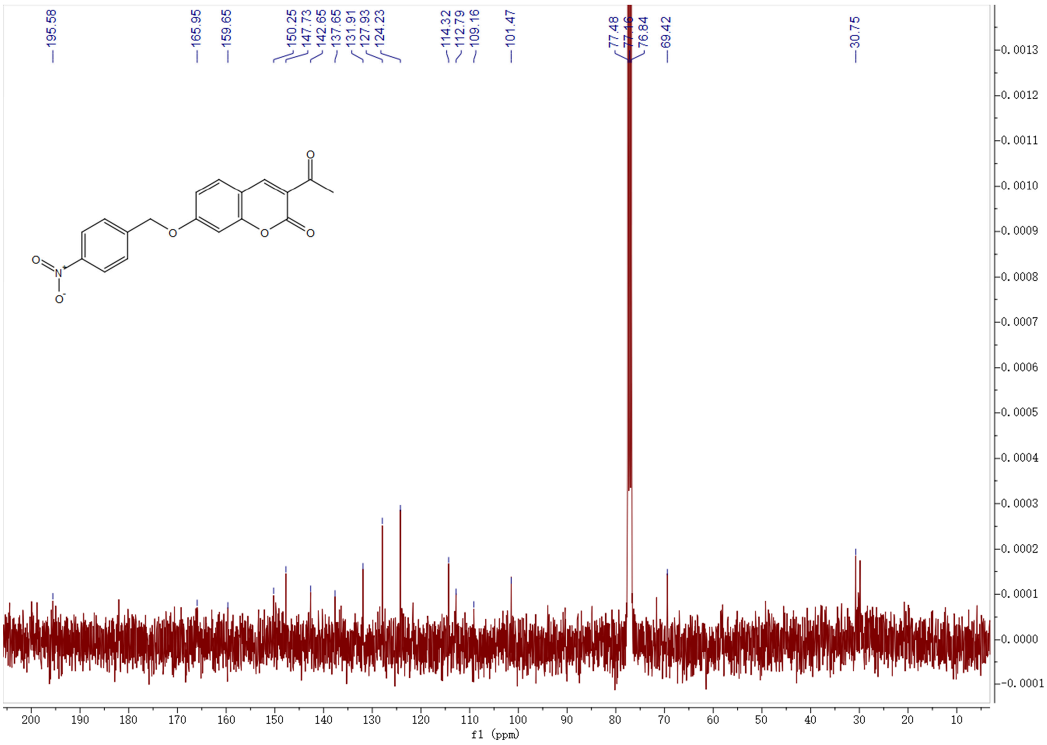

**Figure S26.** $^{13}C$ NMR Spectrum (100 MHz, $CDCl_3$) of Compound **4f**

## 2.14 Figure S27-S28. NMR Spectra of Compound 4g

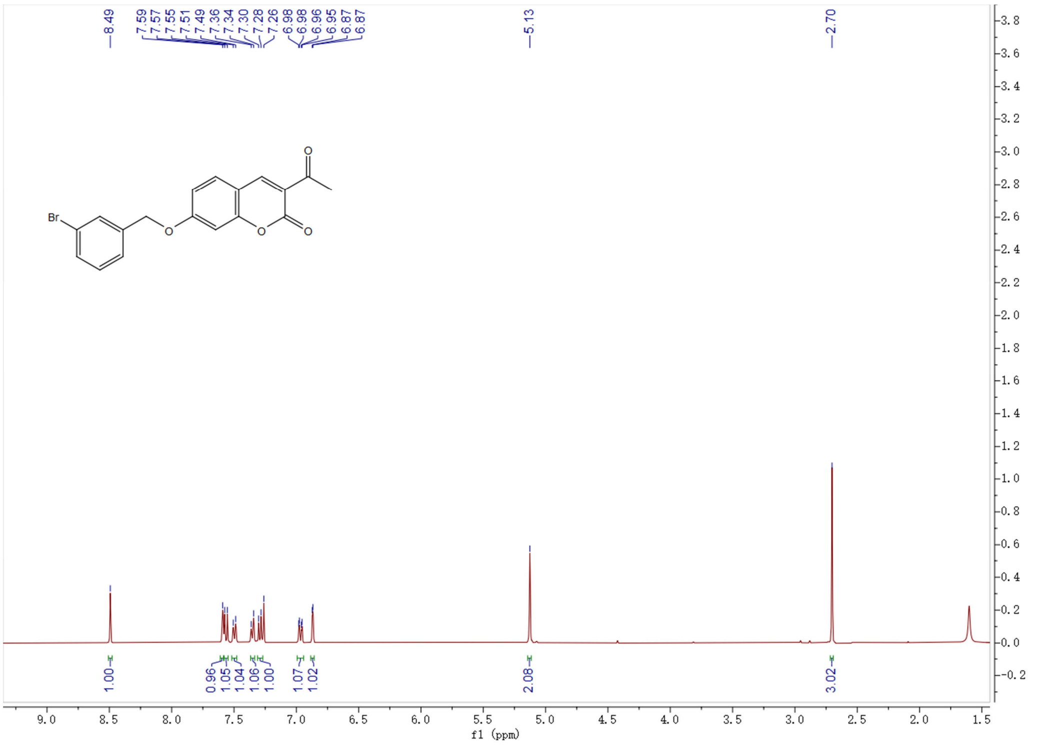

**Figure S27.** $^{1}H$ NMR Spectrum (400 MHz, $CDCl_3$) of Compound **4g**

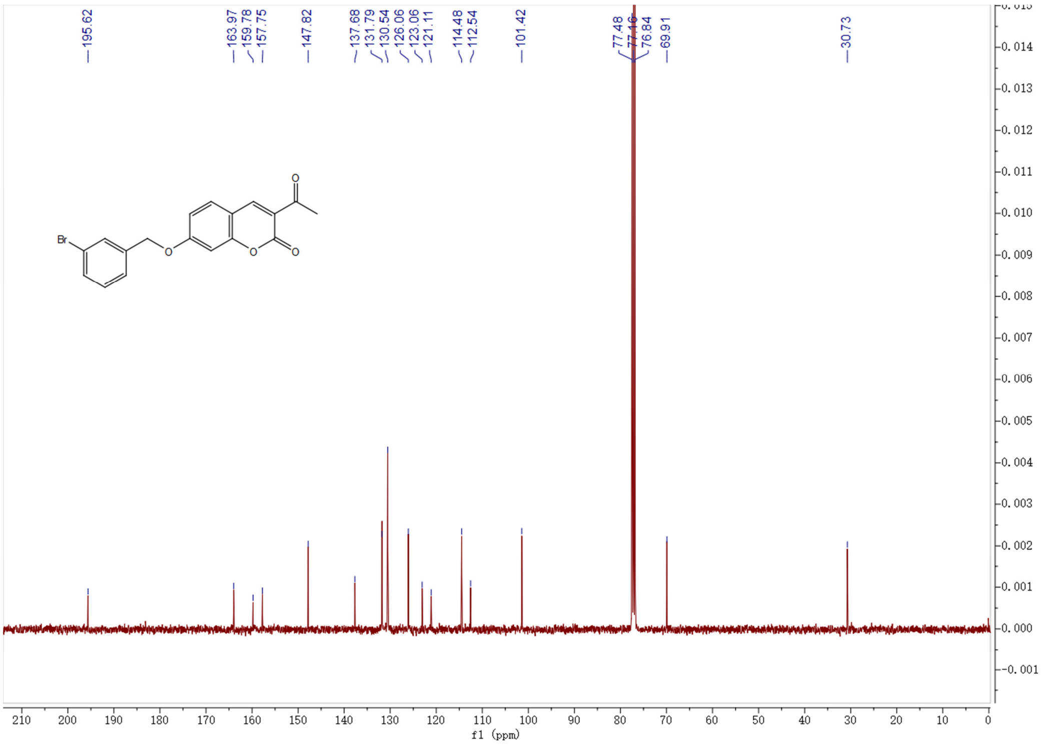

**Figure S28.** $^{13}C$ NMR Spectrum (100 MHz, $CDCl_3$) of Compound **4g**

## 2.15 Figure S29-S30. NMR Spectra of Compound 5a

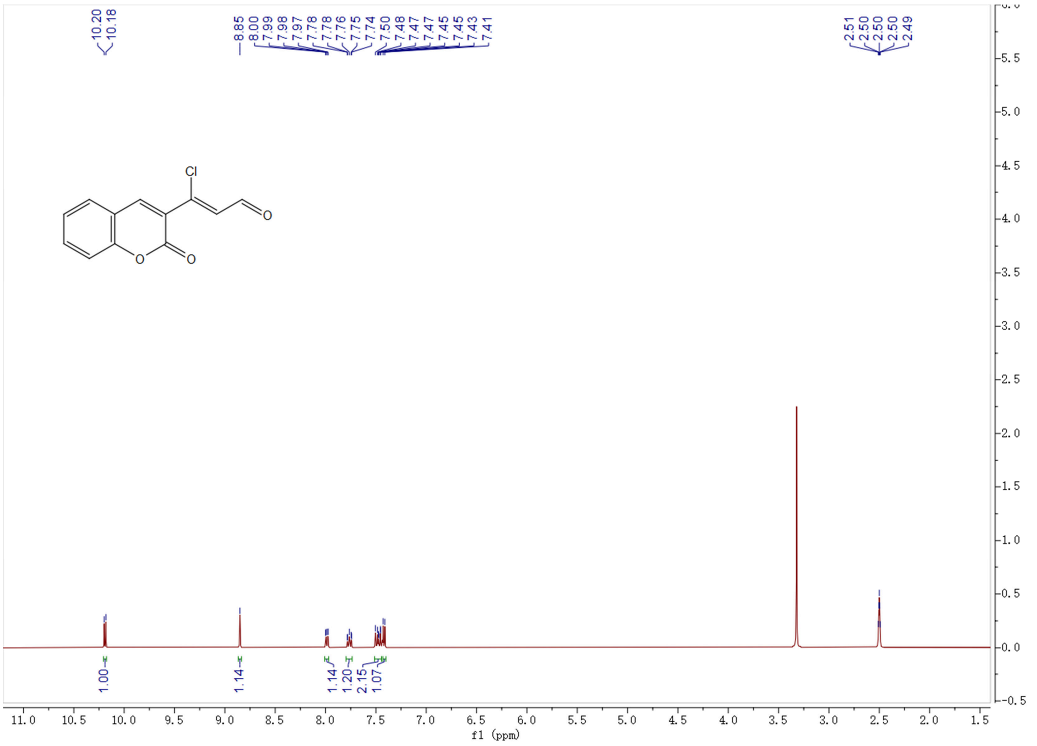

**Figure S29.** $^1$H NMR Spectrum (400 MHz, DMSO-$d_6$) of Compound **5a**

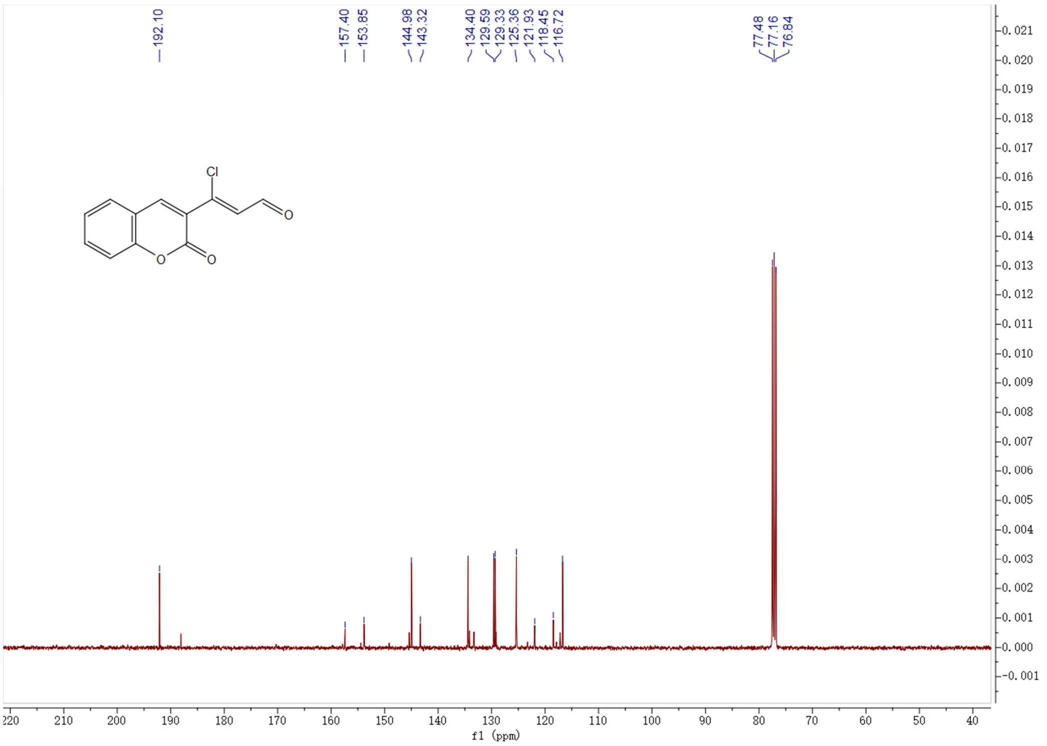

**Figure S30.** $^{13}$C NMR Spectrum (100 MHz, $CDCl_3$) of Compound **5a**

## 2.16 Figure S31-S32. NMR Spectra of Compound 5b

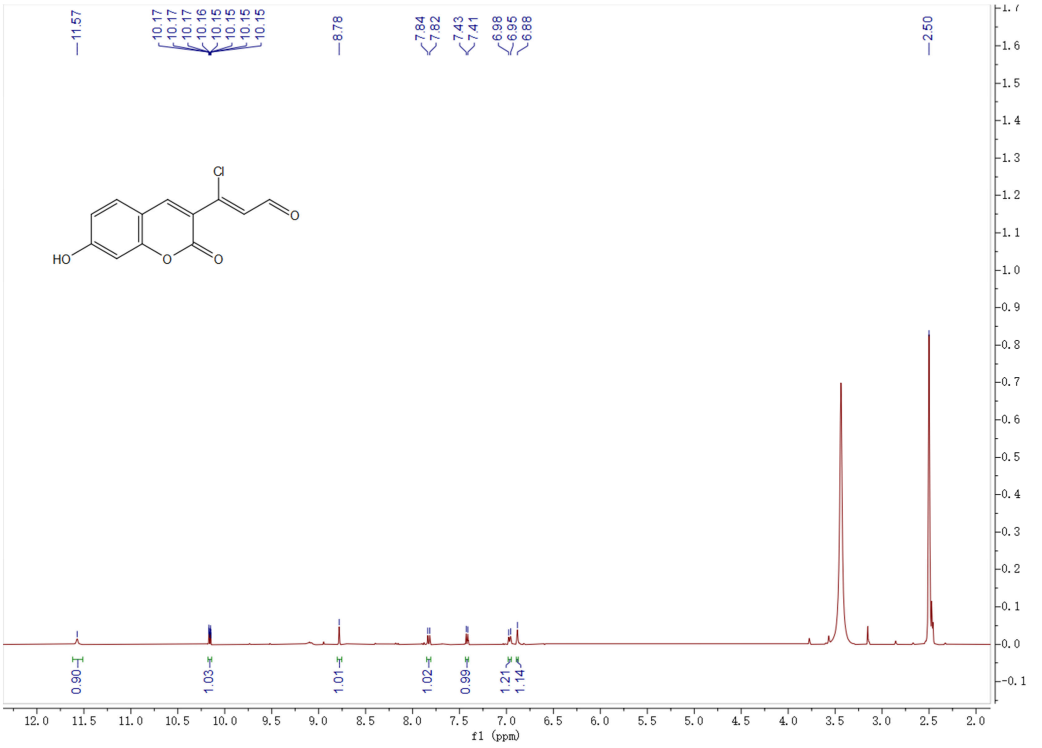

**Figure S31.** $^{1}$H NMR Spectrum (400 MHz, DMSO-$d_6$) of Compound **5b**

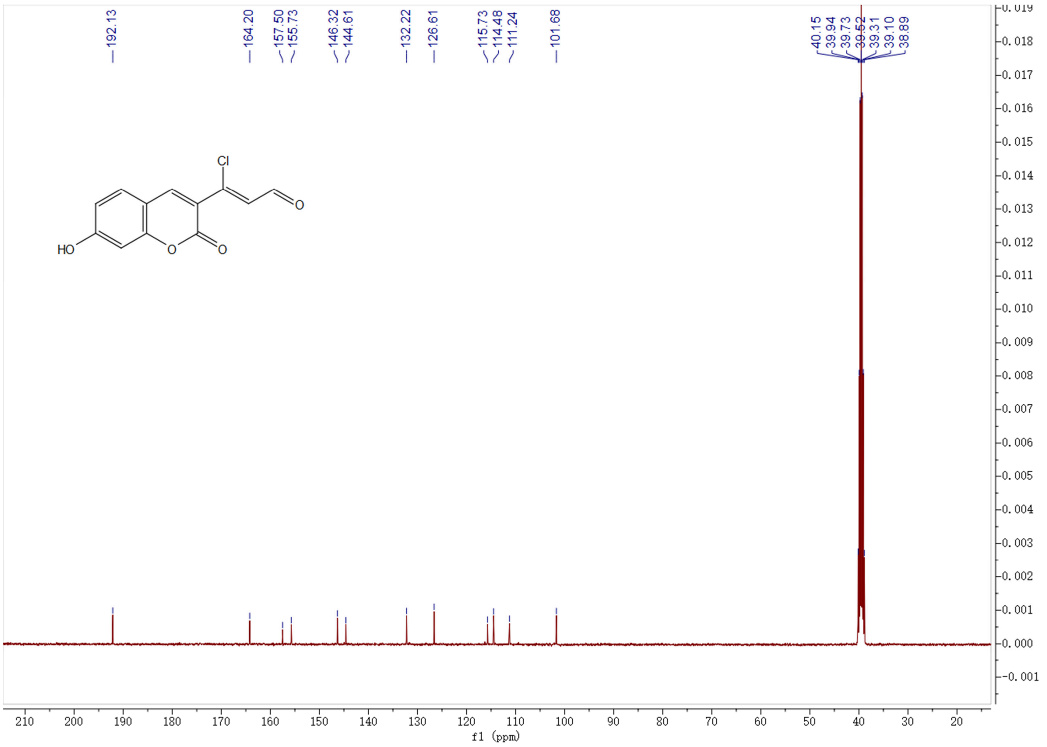

**Figure S32.** $^{13}$C NMR Spectrum (100 MHz, DMSO-$d_6$) of Compound **5b**

## 2.17 Figure S33-S34. NMR Spectra of Compound 5c

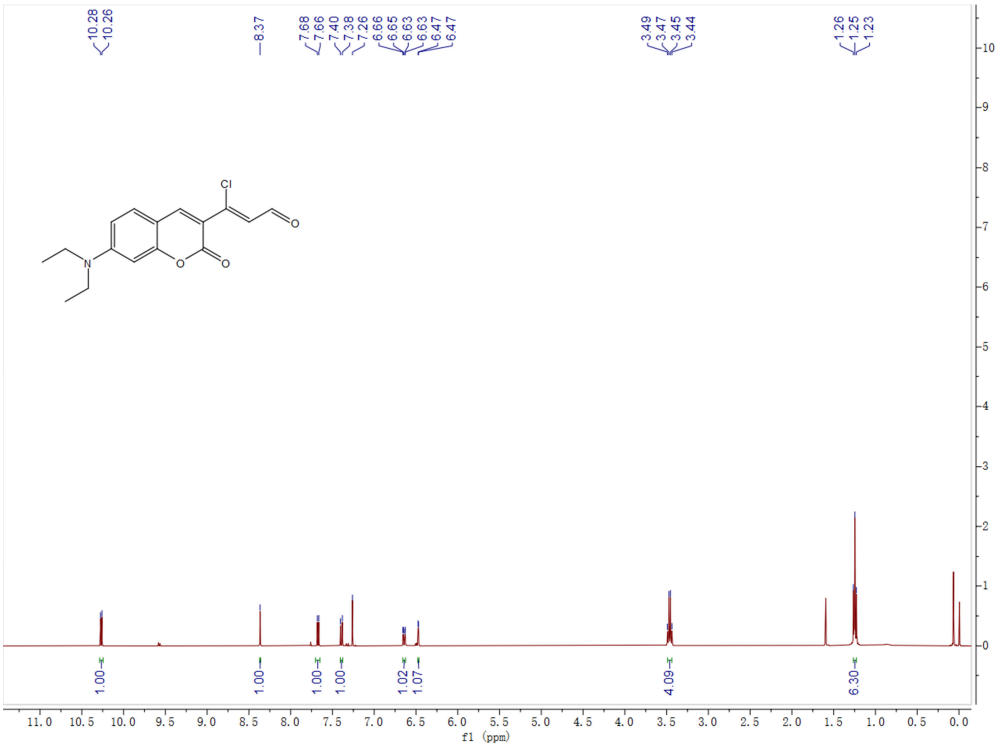

**Figure S33.** $^{1}$H NMR Spectrum (400 MHz, $CDCl_3$) of Compound **5c**

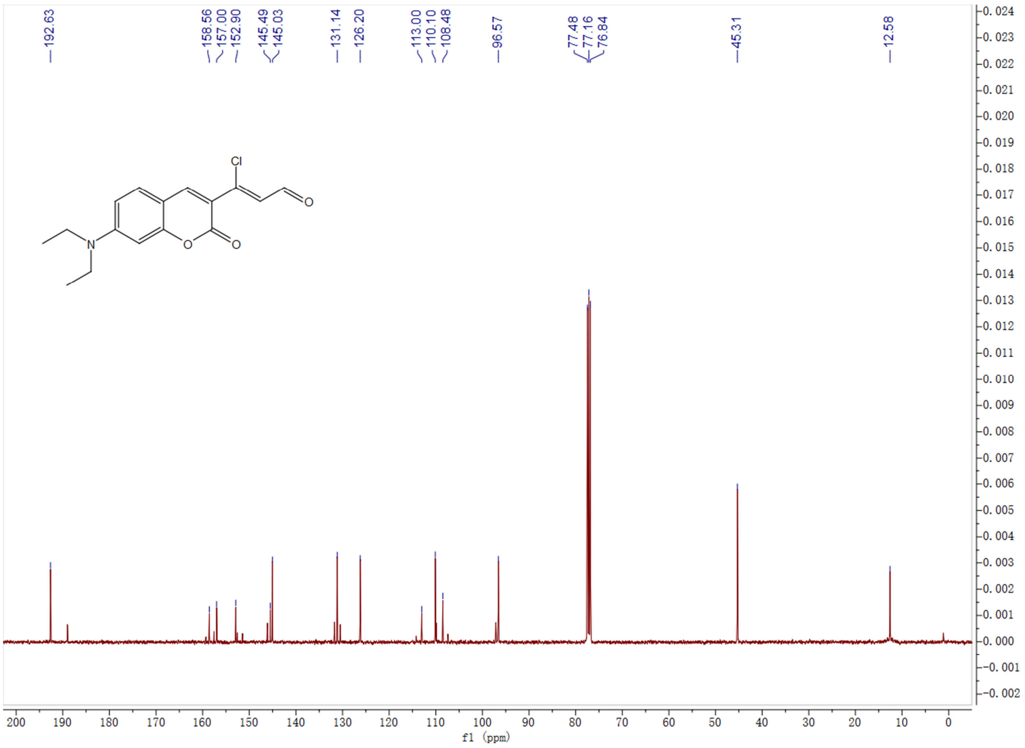

**Figure S34.** $^{13}$C NMR Spectrum (100 MHz, DMSO-$d_6$) of Compound **5c**

## 2.18 Figure S35-S36. NMR Spectra of Compound 5d

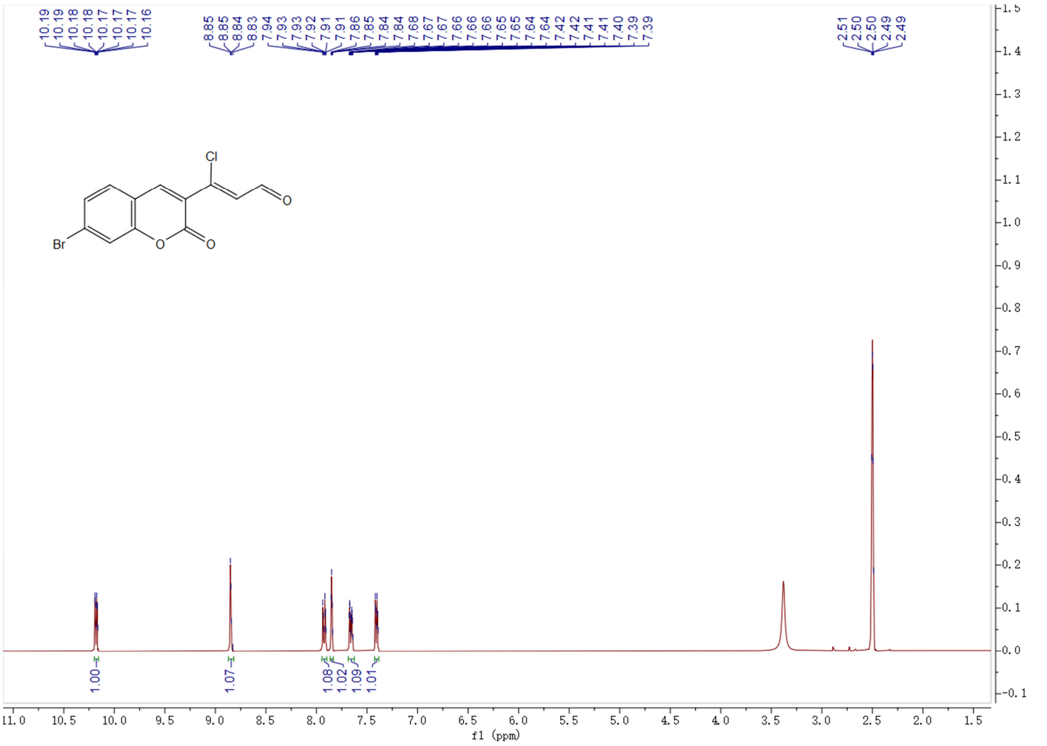

**Figure S35.** $^{1}$H NMR Spectrum (400 MHz, DMSO-$d_6$) of Compound **5d**

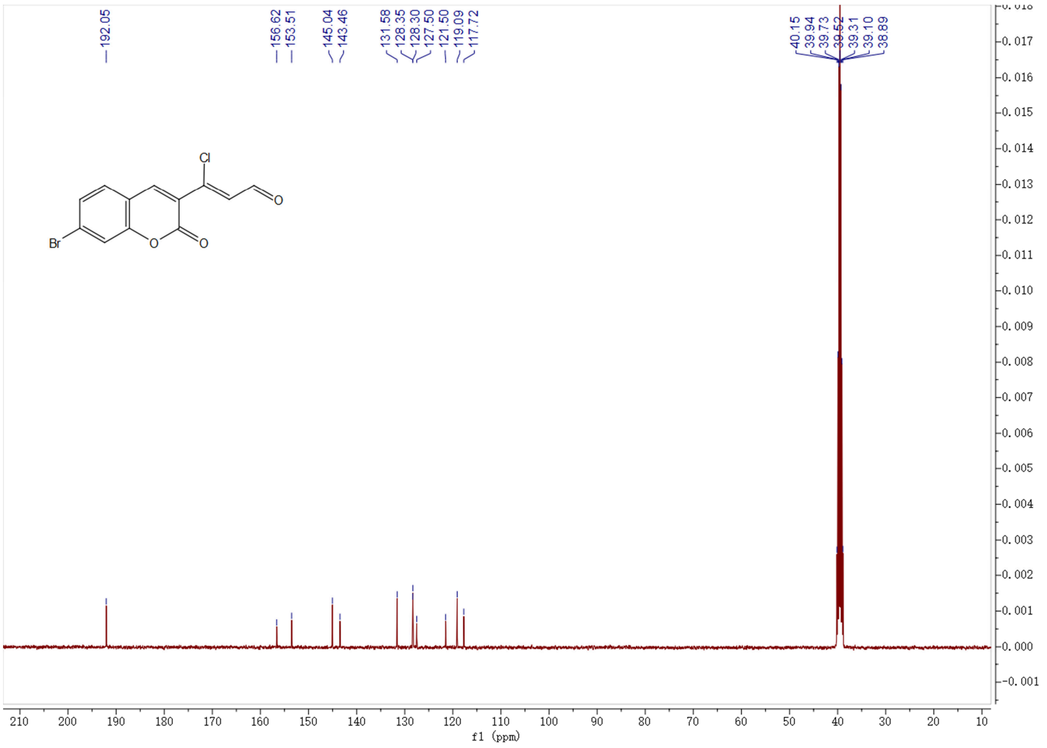

**Figure S36.** $^{13}$C NMR Spectrum (100 MHz, DMSO-$d_6$) of Compound **5d**

## 2.19 Figure S37-S38. NMR Spectra of Compound 5e

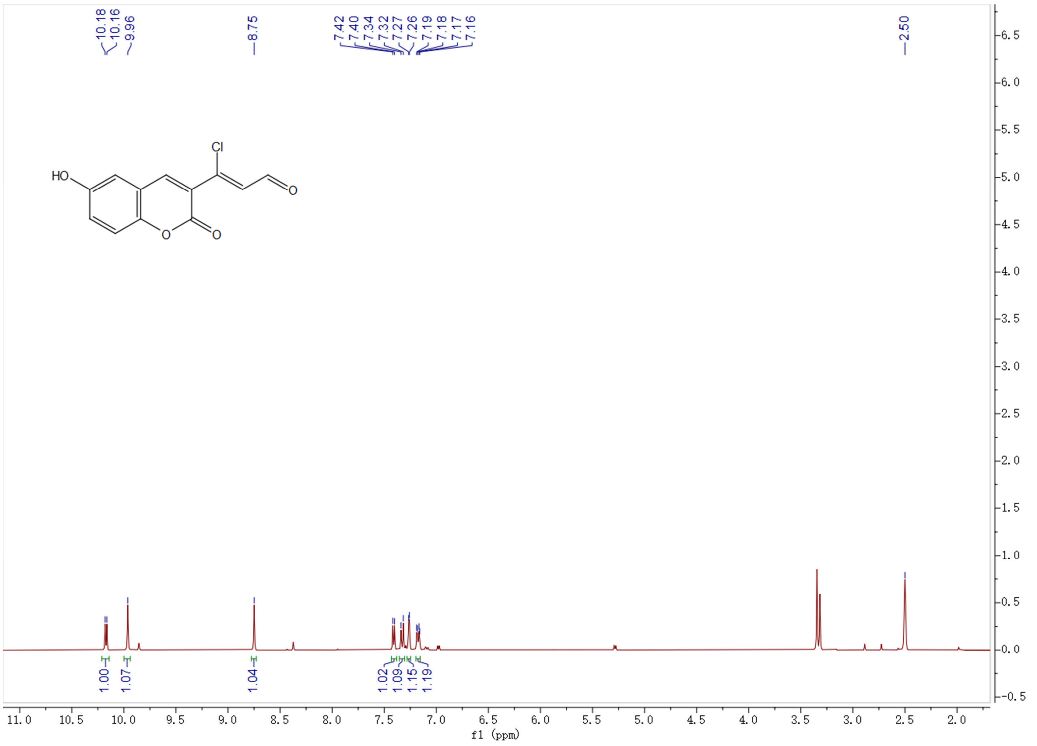


**Figure S37.** $^{1}$H NMR Spectrum (400 MHz, DMSO-$d_6$) of Compound **5e**

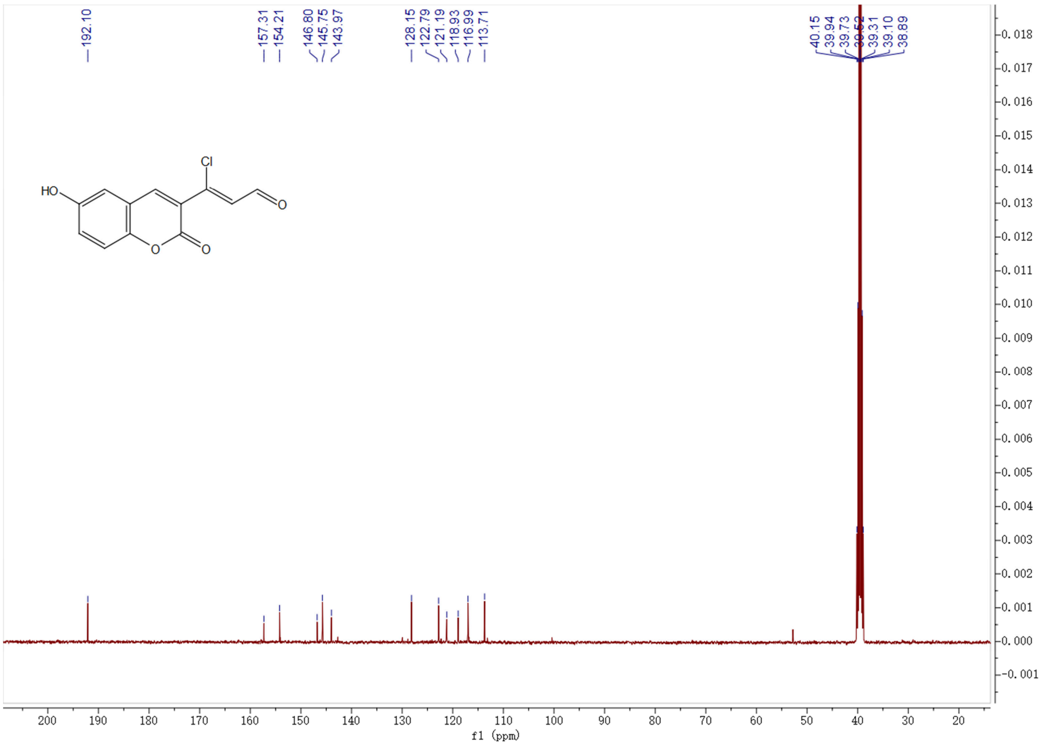


**Figure S38.** $^{13}$C NMR Spectrum (100 MHz, DMSO-$d_6$) of Compound **5e**

## 2.20 Figure S39-S40. NMR Spectra of Compound 5f

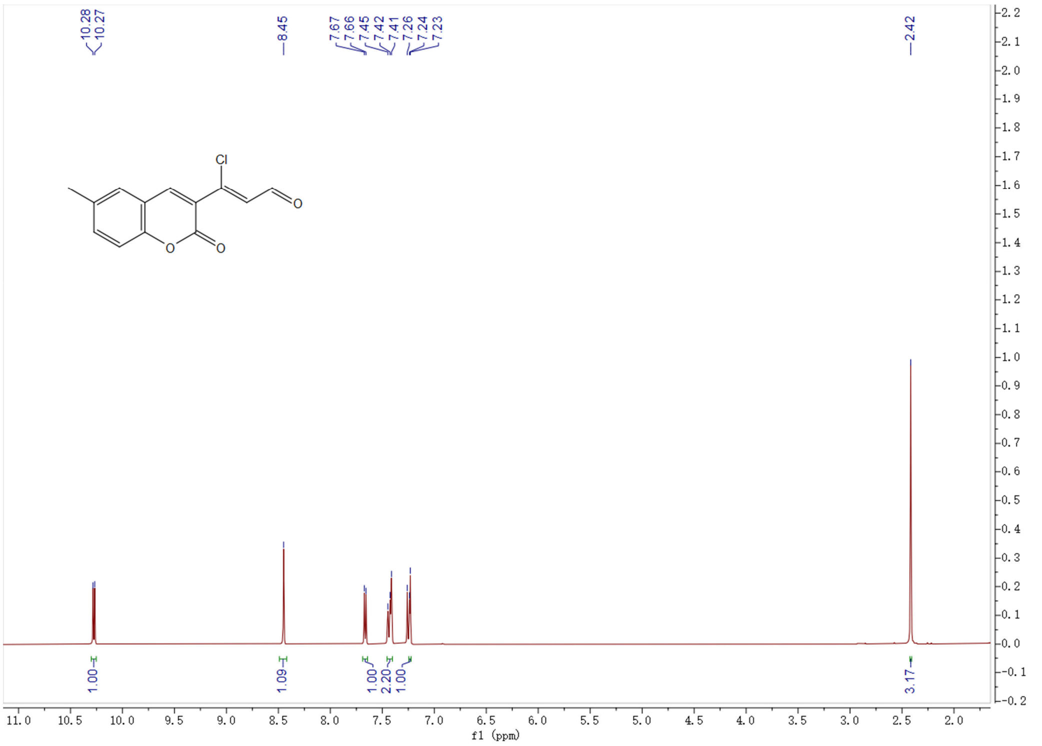


**Figure S39.** $^{1}$H NMR Spectrum (400 MHz, $CDCl_3$) of Compound **5f**

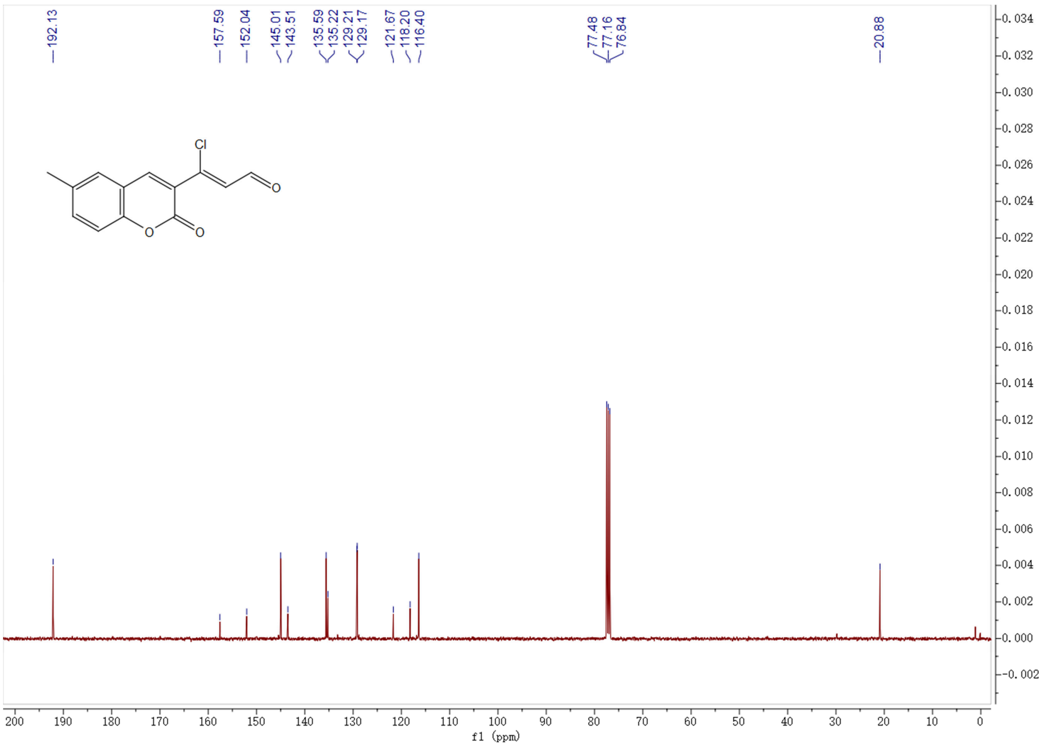


**Figure S40.** $^{13}$C NMR Spectrum (100 MHz, $CDCl_3$) of Compound **5f**

## 2.21 Figure S41-S42. NMR Spectra of Compound 5g

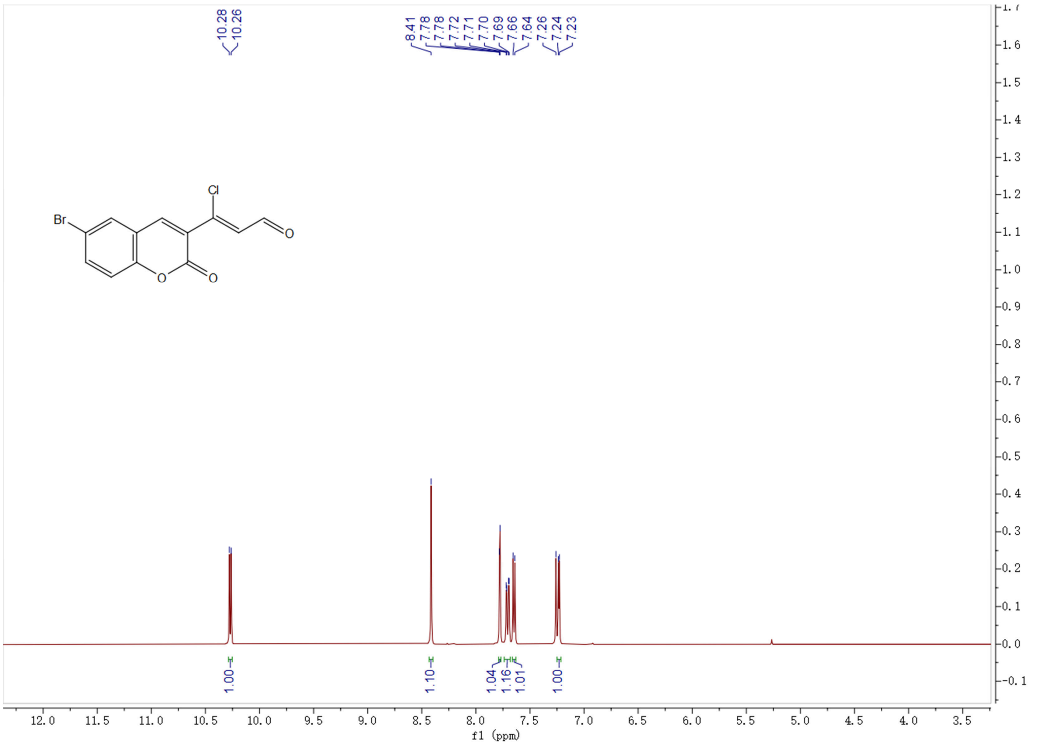


**Figure S41.** $^{1}$H NMR Spectrum (400 MHz, $CDCl_3$) of Compound **5g**

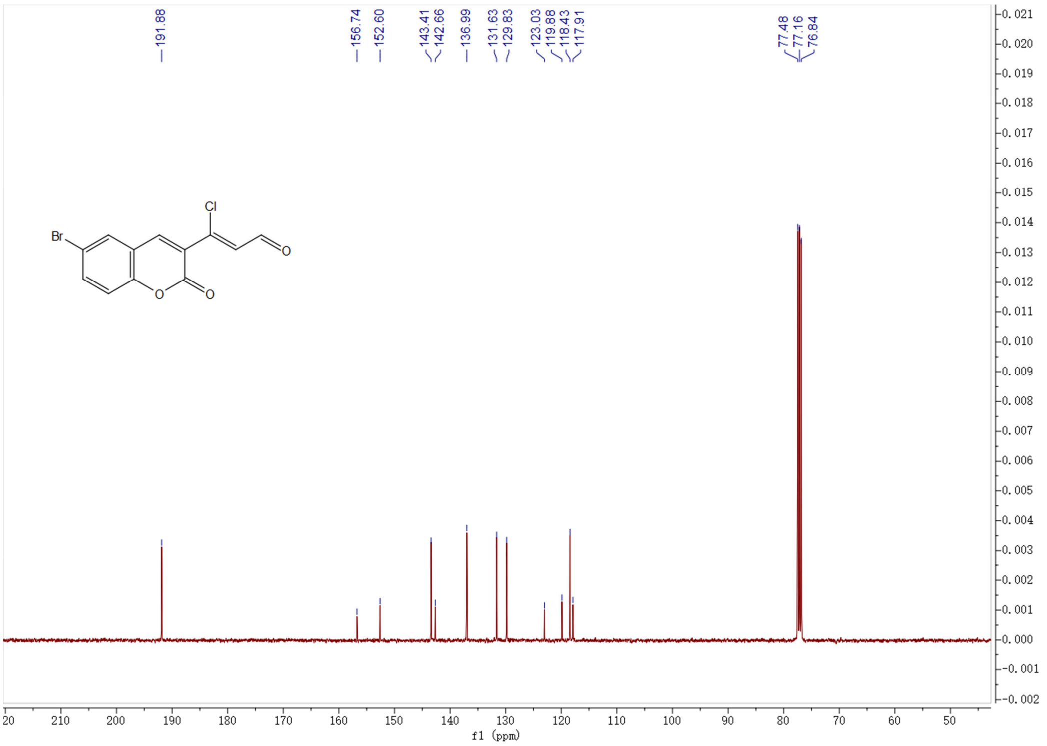


**Figure S42.** $^{13}$C NMR Spectrum (100 MHz, $CDCl_3$) of Compound **5g**

## 2.22 Figure S43-S44. NMR Spectra of Compound 6a

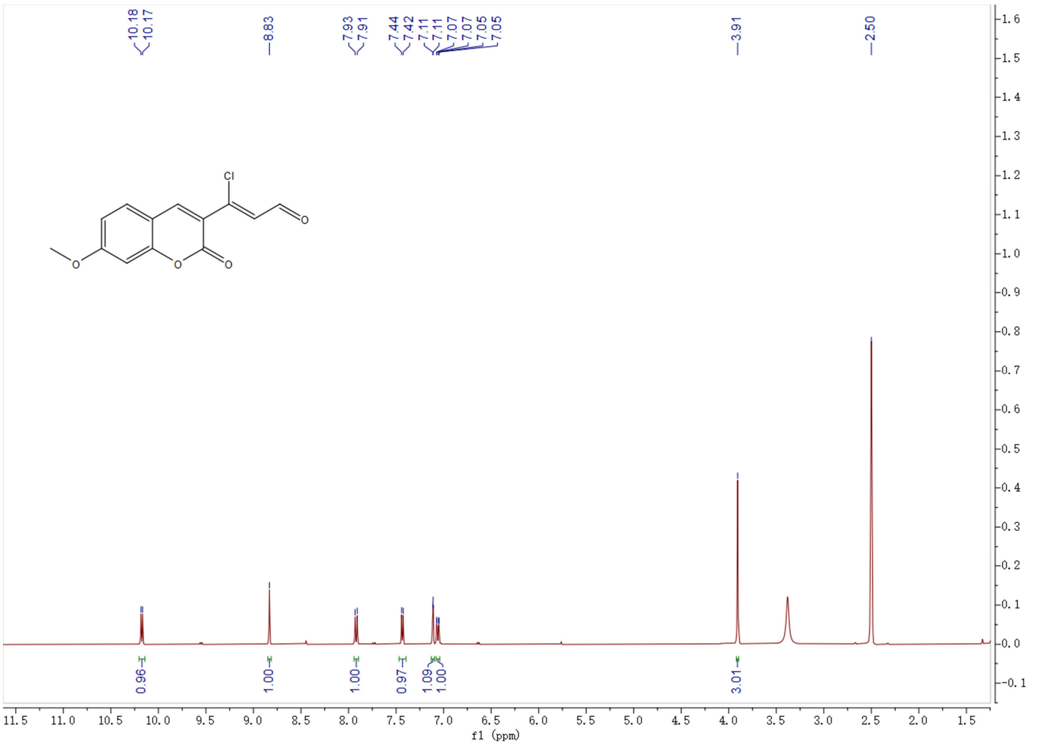


**Figure S43.** $^{1}$H NMR Spectrum (400 MHz, DMSO-$d_6$) of Compound **6a**

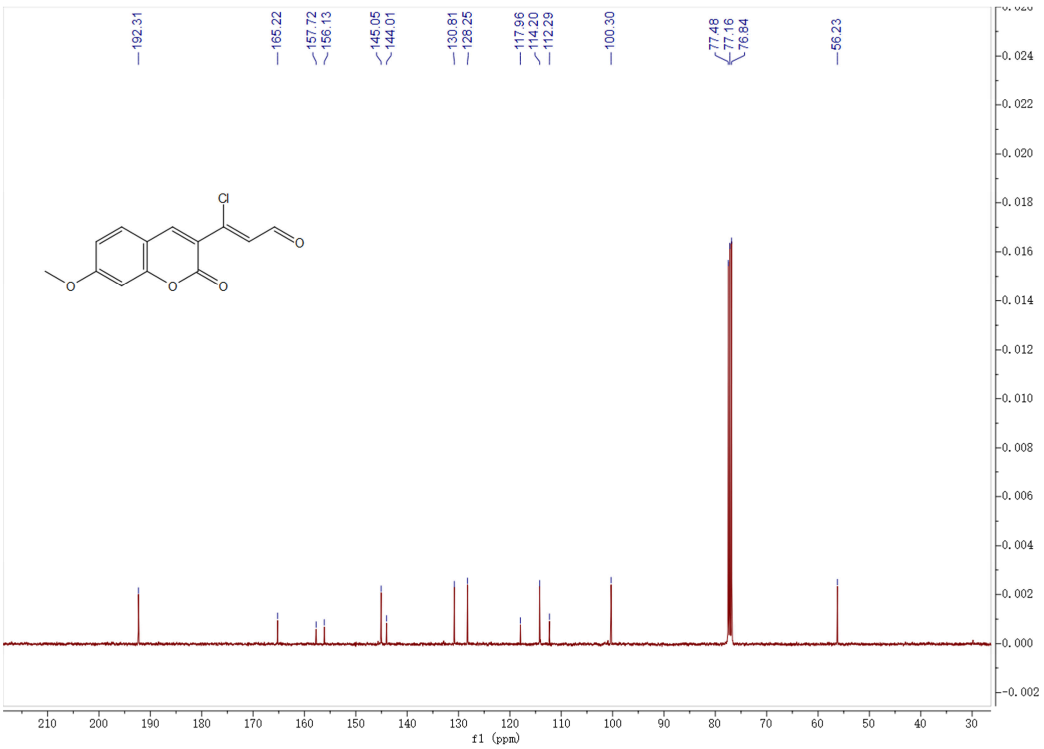


**Figure S44.** $^{13}$C NMR Spectrum (100 MHz, $CDCl_3$) of Compound **6a**

## 2.23 Figure S45-S46. NMR Spectra of Compound 6b

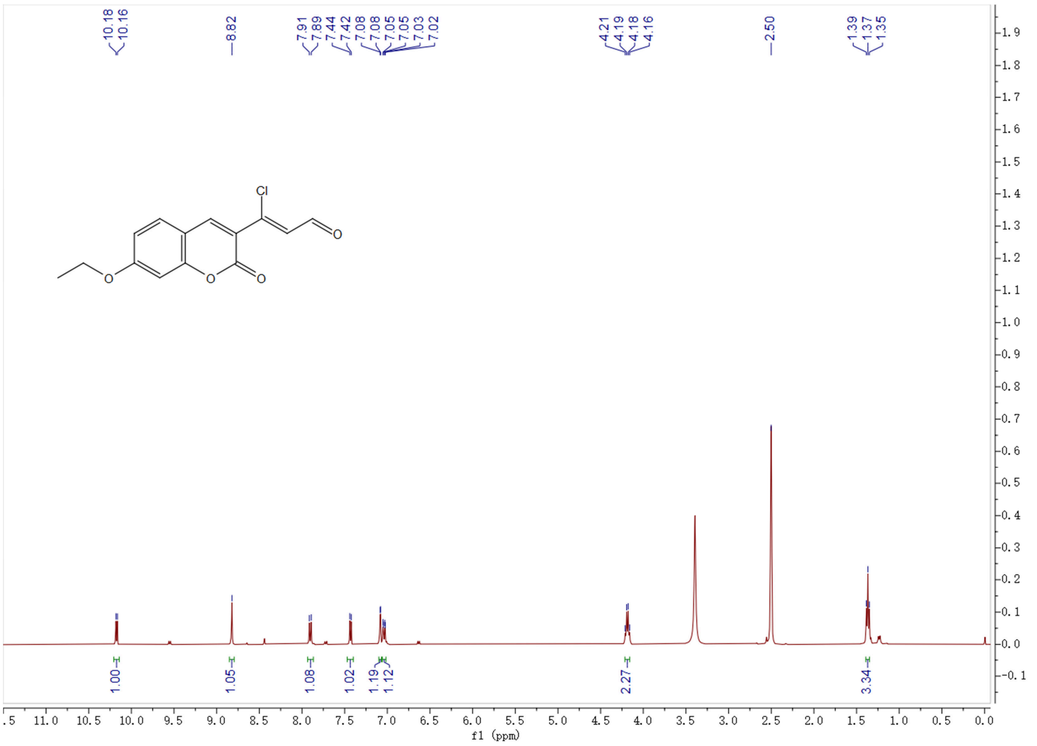

**Figure S45.** $^{1}$H NMR Spectrum (400 MHz, DMSO-$d_6$) of Compound **6b**

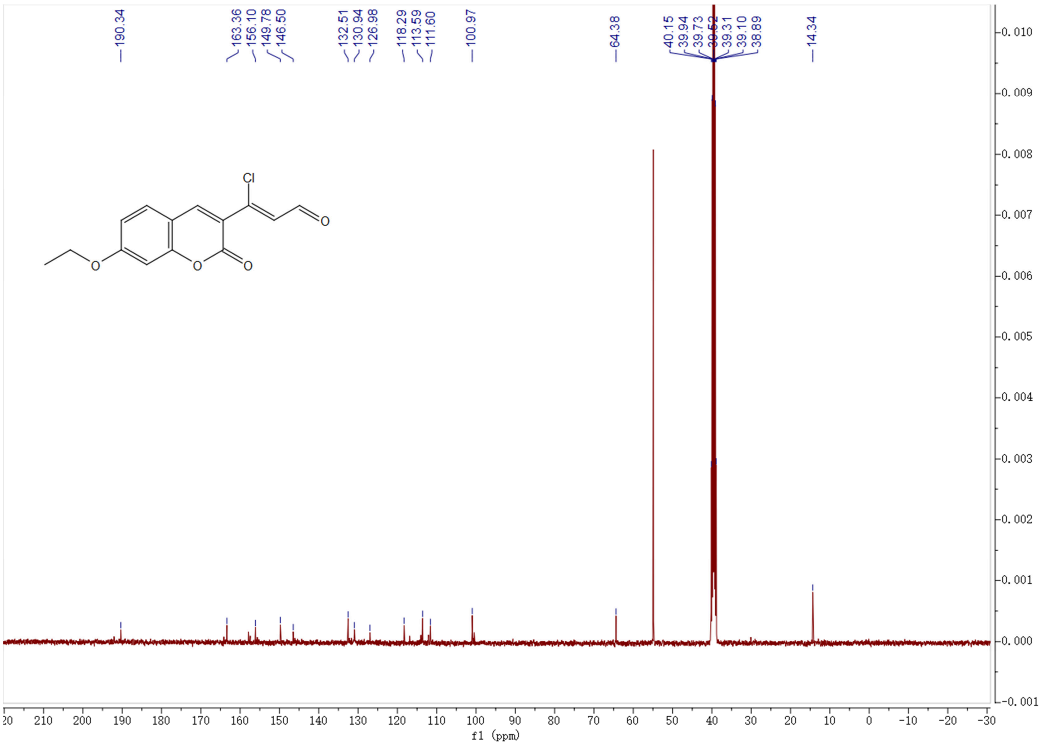

**Figure S46.** $^{13}$C NMR Spectrum (100 MHz, DMSO-$d_6$) of Compound **6b**

## 2.24 Figure S47-S48. NMR Spectra of Compound 6c

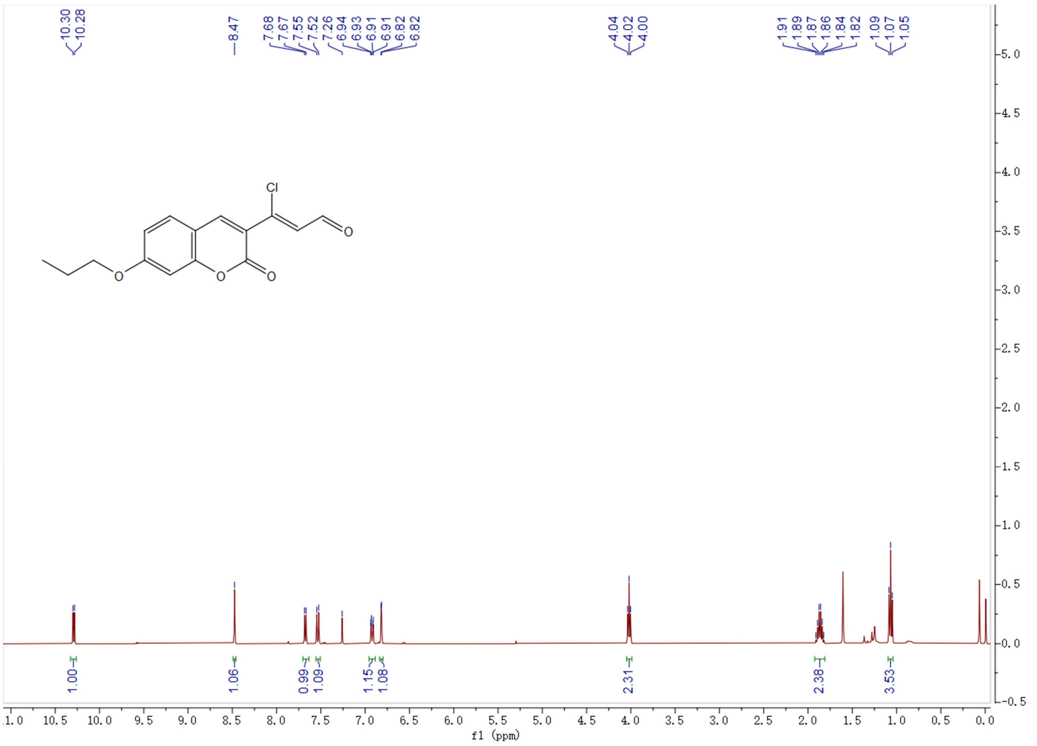

**Figure S47.** $^{1}$H NMR Spectrum (400 MHz, $CDCl_3$) of Compound **6c**

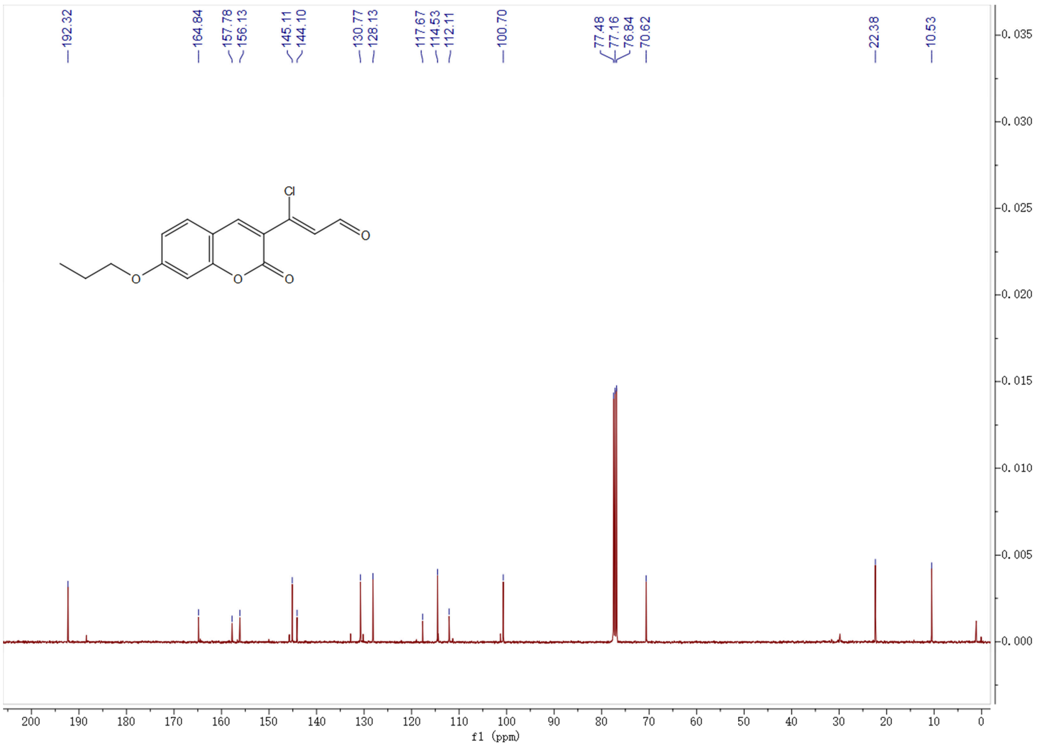

**Figure S48.** $^{13}$C NMR Spectrum (100 MHz, $CDCl_3$) of Compound **6c**

## 2.25 Figure S49-S50. NMR Spectra of Compound 6d

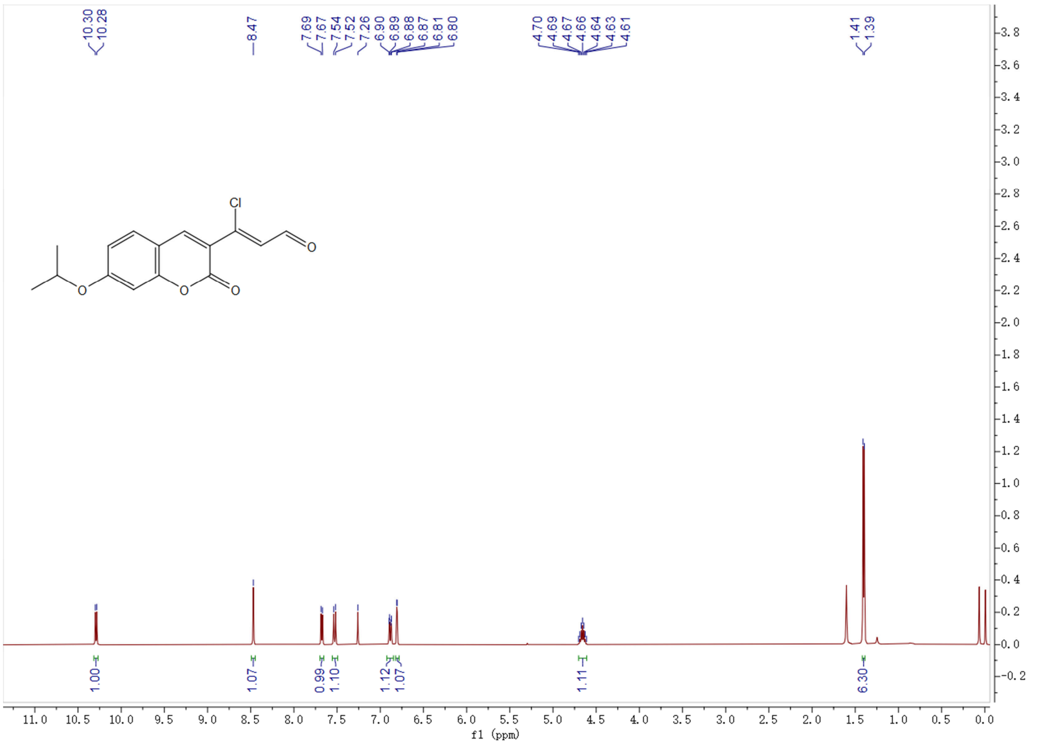


**Figure S49.** $^{1}H$ NMR Spectrum (400 MHz, $CDCl_3$) of Compound **6d**

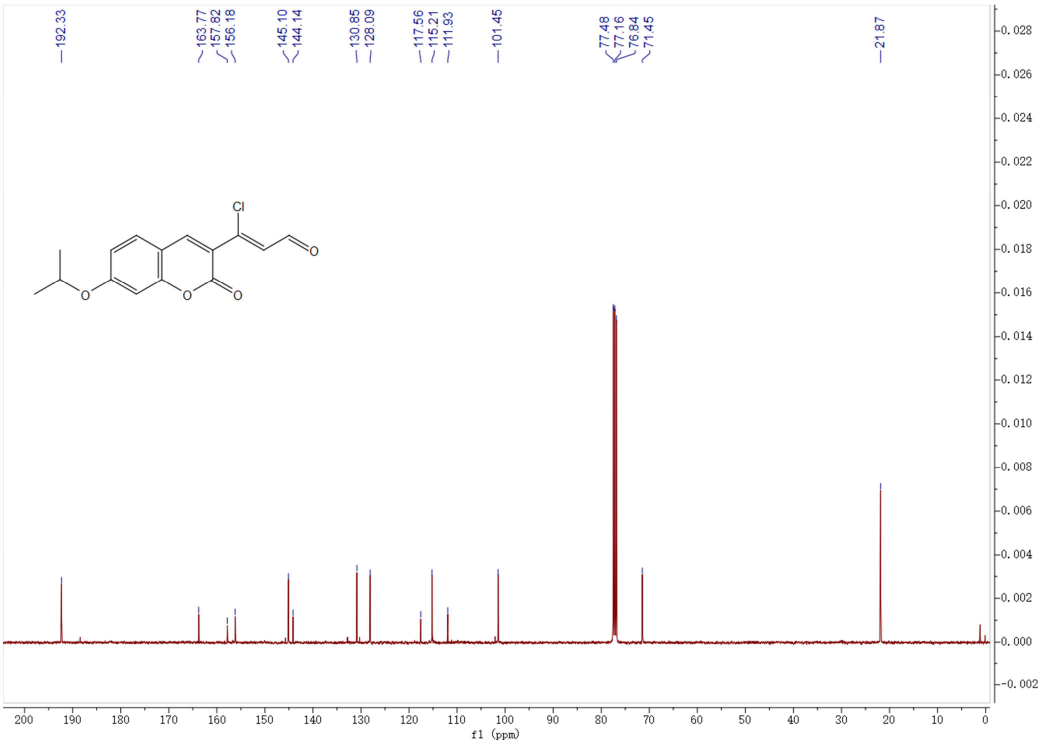


**Figure S50.** $^{13}C$ NMR Spectrum (100 MHz, $CDCl_3$) of Compound **6d**

## 2.26 Figure S51-S52. NMR Spectra of Compound 6e

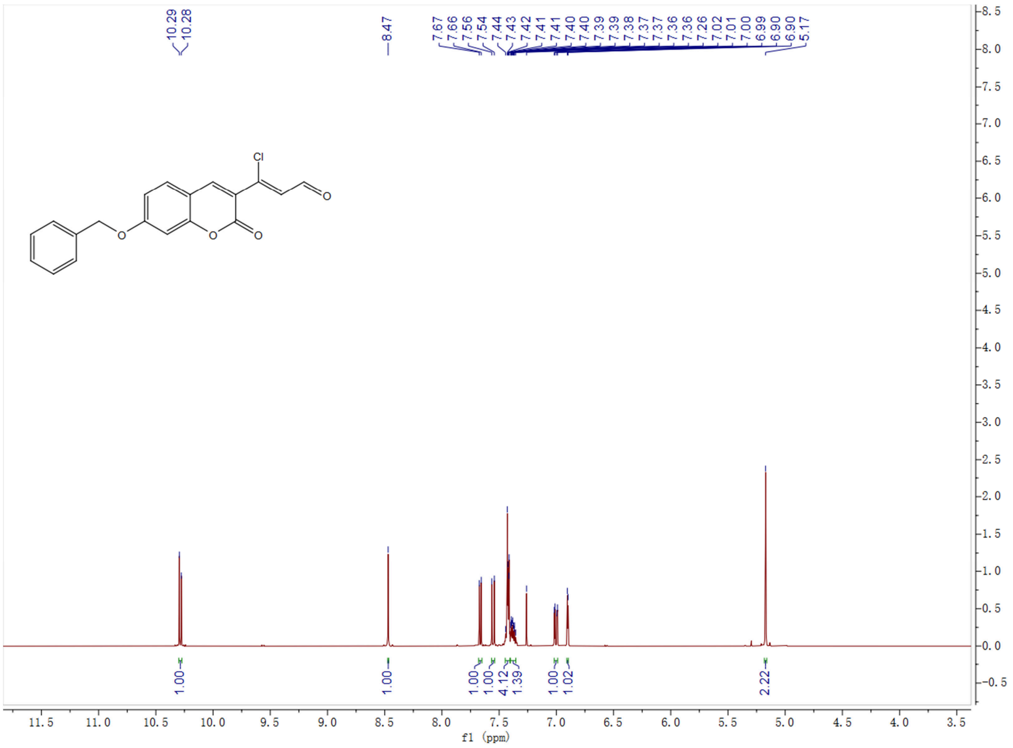

**Figure S51.** $^{1}$H NMR Spectrum (400 MHz, $CDCl_3$) of Compound **6e**

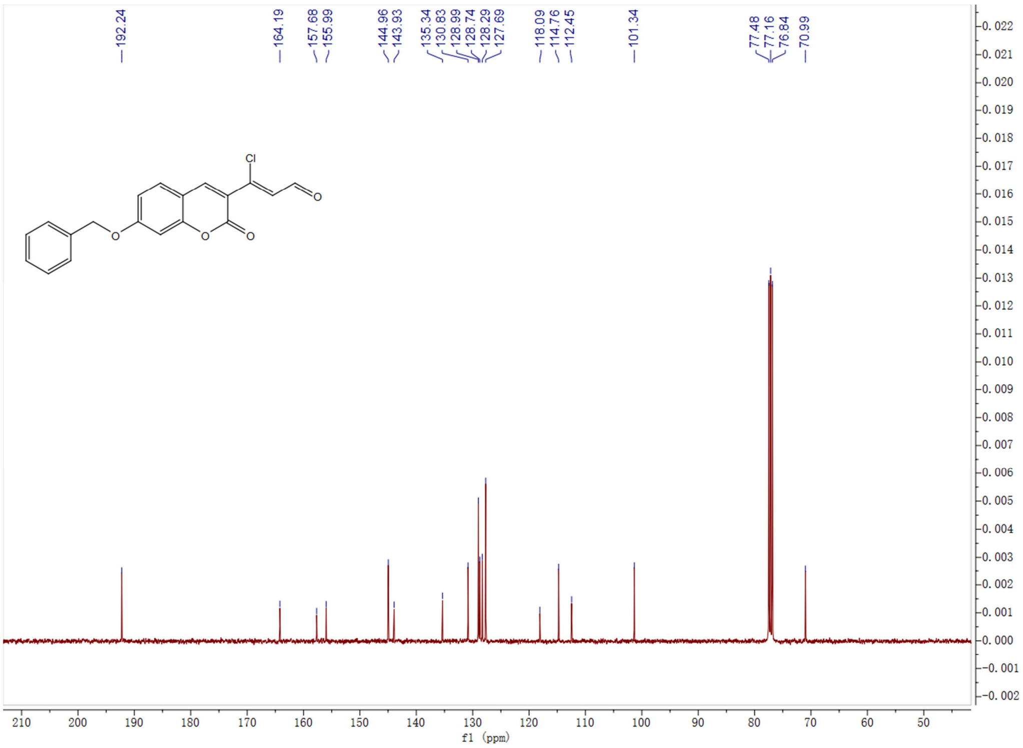

**Figure S52.** $^{13}$C NMR Spectrum (100 MHz, $CDCl_3$) of Compound **6e**

## 2.27 Figure S53-S54. NMR Spectra of Compound 6f

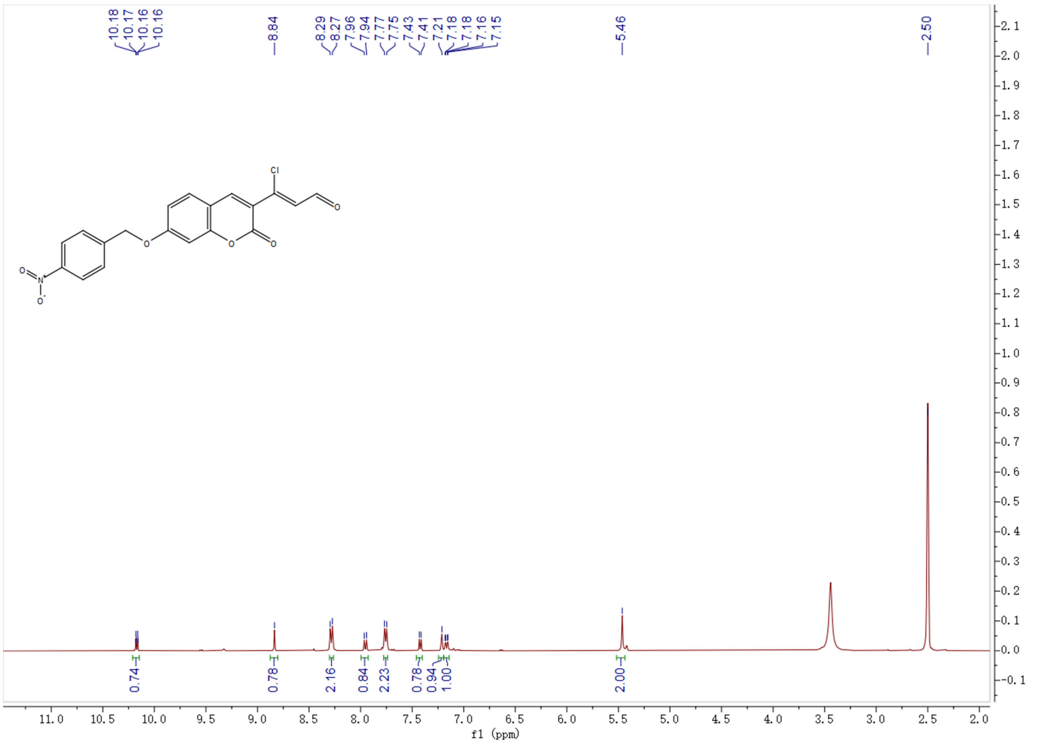

**Figure S53.** $^{1}H$ NMR Spectrum (400 MHz, DMSO-$d_6$) of Compound **6f**

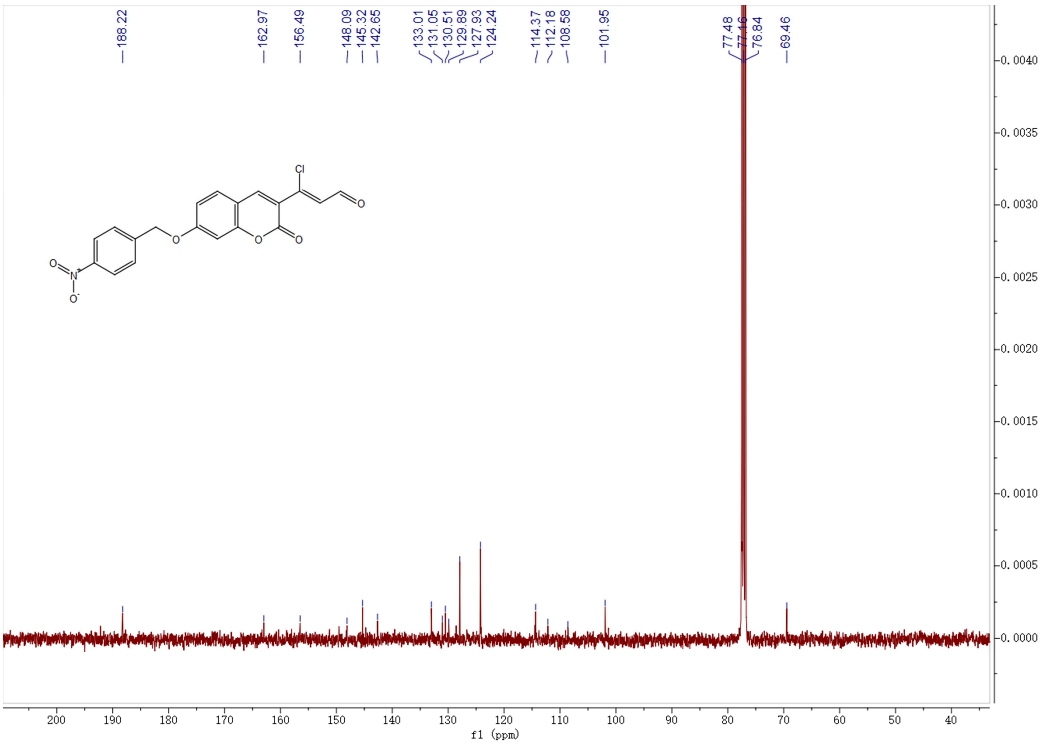

**Figure S54.** $^{13}C$ NMR Spectrum (100 MHz, $CDCl_3$) of Compound **6f**

## 2.28 Figure S55-S56. NMR Spectra of Compound 6g

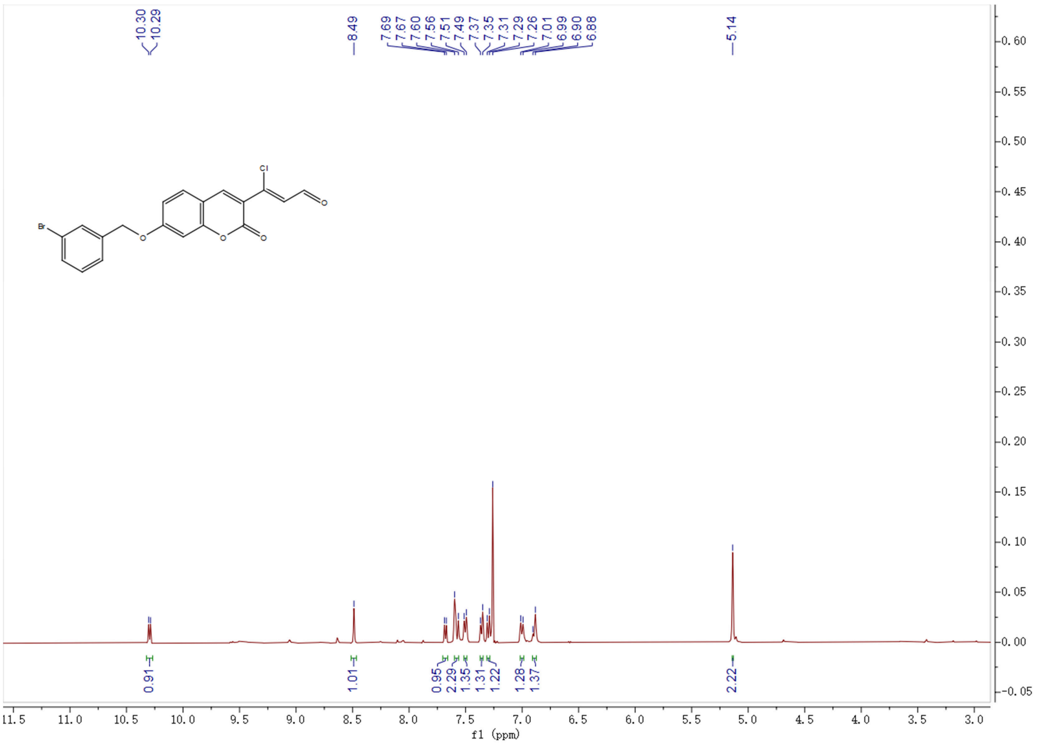


**Figure S55.** $^{1}$H NMR Spectrum (400 MHz, $CDCl_3$) of Compound **6g**

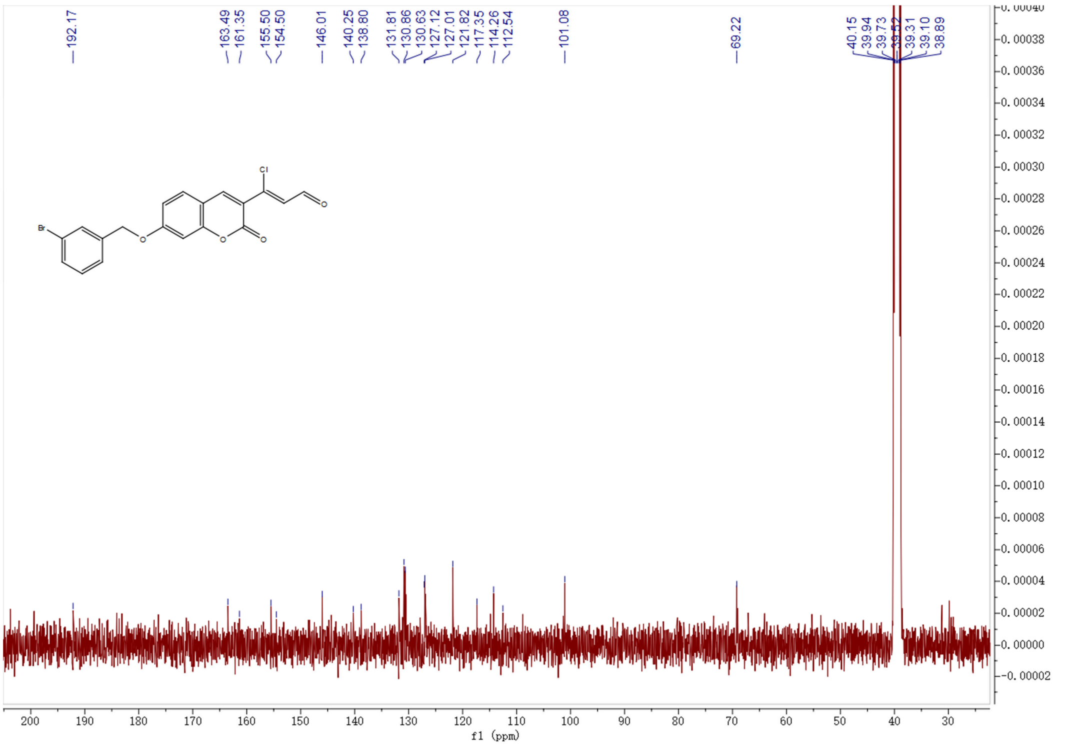


**Figure S56.** $^{13}$C NMR Spectrum (100 MHz, DMSO-$d_6$) of Compound **6g**

## 3 Network Construction and Targets Prediction

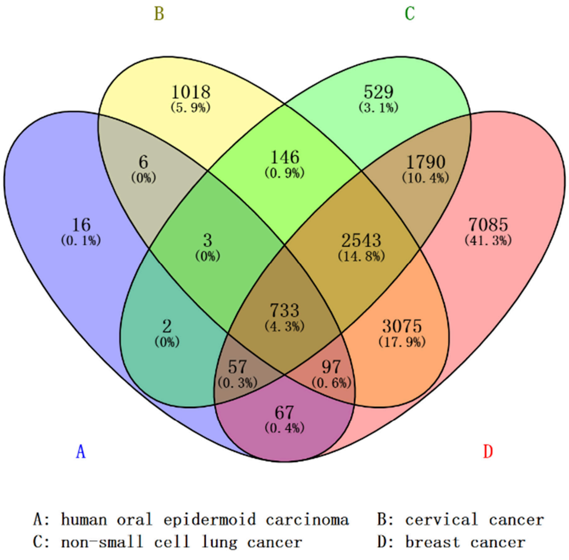


**Figure 1.** Prediction of potential targets of compound **6e** on four types of cancer

**TABLE 1**. The predicted core targets of compound **6e** in the oral epidermoid carcinoma cell line (Top fifteen).

| NO. | Gene Symbol | Degree in PPI Network |
|---|---|---|
| 1 | SRC | 111 |
| 2 | MAPK1 | 101 |
| 3 | PTPN11 | 86 |
| 4 | GRB2 | 84 |
| 5 | EGFR | 82 |
| 6 | HSP90AA1 | 80 |
| 7 | RHOA | 80 |
| 8 | ESR1 | 74 |
| 9 | CASP3 | 71 |
| 10 | MAPK8 | 67 |
| 11 | MAPK14 | 67 |
| 12 | CTNNA1 | 65 |
| 13 | IGF1 | 64 |
| 14 | MET | 61 |
| 15 | STAT1 | 58 |
