# Supplementary figures and images for: Design, synthesis and anticancer activity studies of 3-(coumarin-3-yl)-acrolein derivatives: Evidenced by integrating network pharmacology and vitro assay
Source: Front Pharmacol. 2023 Mar 23;14:1141121. doi: 10.3389/fphar.2023.1141121 (PMC10076643; doi:10.3389/fphar.2023.1141121)

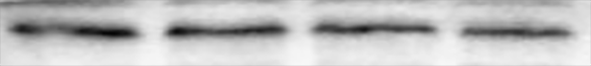

Supplement: Supplementary file 2 [file DataSheet2.ZIP › Frontiers WB Blot/AKT.tif]

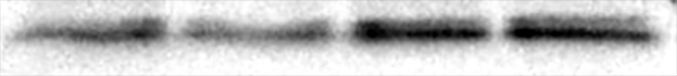

Supplement: Supplementary file 2 [file DataSheet2.ZIP › Frontiers WB Blot/Bax.tif]

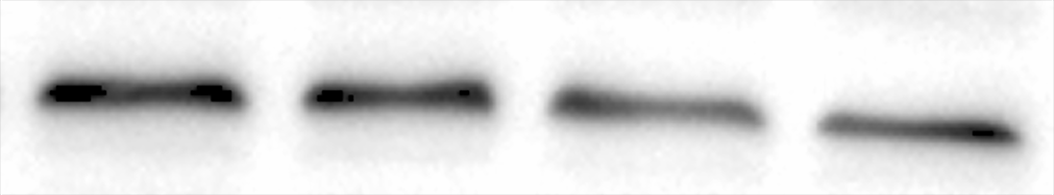

Supplement: Supplementary file 2 [file DataSheet2.ZIP › Frontiers WB Blot/Bcl-2.tif]

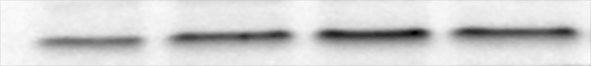

Supplement: Supplementary file 2 [file DataSheet2.ZIP › Frontiers WB Blot/Caspase 3ú¿17 KDú⌐.tif]

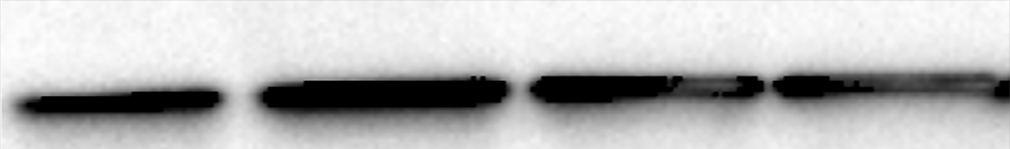

Supplement: Supplementary file 2 [file DataSheet2.ZIP › Frontiers WB Blot/Caspase 3ú¿35 KDú⌐.tif]

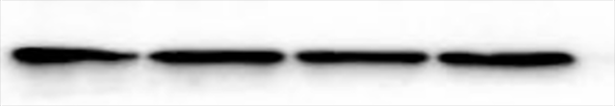

Supplement: Supplementary file 2 [file DataSheet2.ZIP › Frontiers WB Blot/GAPDH.tif]

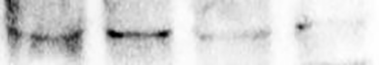

Supplement: Supplementary file 2 [file DataSheet2.ZIP › Frontiers WB Blot/pAKT.tif]

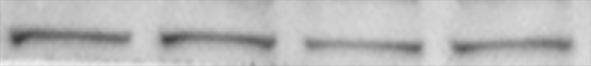

Supplement: Supplementary file 2 [file DataSheet2.ZIP › Frontiers WB Blot/PI3K.tif]

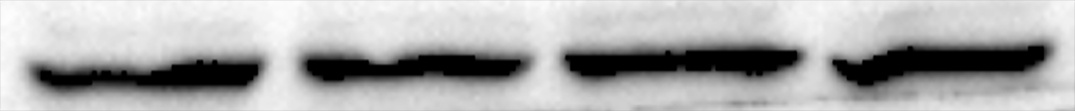

Supplement: Supplementary file 2 [file DataSheet2.ZIP › Frontiers WB Blot/a┬-actin.tif]
